# Tripeptidyl peptidase II coordinates the homeostasis of calcium and lipids in the central nervous system and its depletion causes presenile dementia in female mice through calcium/lipid dyshomeostasis-induced autophagic degradation of CYP19A1

**DOI:** 10.7150/thno.92571

**Published:** 2024-01-27

**Authors:** Jin Zhao, Chengtong He, Xueyu Fan, Lin Wang, Liao Zhao, Hui Liu, Wujun Shen, Sanwei Jiang, Kaixuan Pei, Jingjing Gao, Yawei Qi, Yang Liu, Junqiang Zhao, Ruiling Zhang, Chengbiao Lu, Jia Tong, Jisen Huai

**Affiliations:** 1 The Second Affiliated Hospital of Xinxiang Medical University (Henan Mental Hospital), Xinxiang, 453000, PR China.; 2 Institute of Psychiatry and Neuroscience, Xinxiang Medical University, Xinxiang, 453003, PR China.; 3 Henan International Key Laboratory for Noninvasive Neuromodulation, Department of Physiology & Pathology, Xinxiang Medical University, Xinxiang, PR China.; 4 Senior author for electrophysiological experiments and related analysis.

**Keywords:** Tripeptidyl peptidase II, Ca^2+^, Phosphatidylcholine, Aromatase, Dementia

## Abstract

**Rationale:** Tripeptidyl peptidase II (TPP2) has been proven to be related to human immune and neurological diseases. It is generally considered as a cytosolic protein which forms the largest known protease complex in eukaryotic cells to operate mostly downstream of proteasomes for degradation of longer peptides. However, this canonical function of TPP2 cannot explain its role in a wide variety of biological and pathogenic processes. The mechanistic interrelationships and hierarchical order of these processes have yet to be clarified.

**Methods:** Animals, cells, plasmids, and viruses established and/or used in this study include: TPP2 knockout mouse line, TPP2 conditional knockout mouse lines (different neural cell type oriented), TRE-TPP2 knockin mouse line on the C57BL/6 background; 293T cells with depletion of TPP2, ATF6, IRE1, PERK, SYVN1, UCHL1, ATG5, CEPT1, or CCTα, respectively; 293T cells stably expressing TPP2, TPP2 S449A, TPP2 S449T, or CCTα-KDEL proteins on the TPP2-depleted background; Plasmids for eukaryotic transient expression of rat CYP19A1-Flag, CYP19A1 S118A-Flag, CYP19A1 S118D-Flag, Sac I ML GFP Strand 11 Long, OMMGFP 1-10, G-CEPIA1er, GCAMP2, CEPIA3mt, ACC-GFP, or SERCA1-GFP; AAV2 carrying the expression cassette of mouse CYP19A1-3 X Flag-T2A-ZsGreen. Techniques used in this study include: Flow cytometry, Immunofluorescence (IF) staining, Immunohistochemical (IHC) staining, Luxol fast blue (LFB) staining, β-galactosidase staining, Lipid droplet (LD) staining, Calcium (Ca^2+^) staining, Stimulated emission depletion (STED) imaging, Transmission electron microscopic imaging, Two-photon imaging, Terminal deoxynucleotidyl transferase (TdT) dUTP nick-end Labeling (TUNEL) assay, Bromodeoxyuridine (BrdU) assay, Enzymatic activity assay, Proximity ligation assay (PLA), In vivo electrophysiological recording, Long-term potentiation (LTP) recording, Split-GFP-based mitochondria-associated membrane (MAM) detection, Immunoprecipitation (IP), Cellular fractionation, In situ hybridization, Semi-quantitative RT-PCR, Immunoblot, Mass spectrometry-based lipidomics, metabolomics, proteomics, Primary hippocampal neuron culture and Morris water maze (MWM) test.

**Results:** We found that TPP2, independent of its enzymatic activity, plays a crucial role in maintaining the homeostasis of intracellular Ca^2+^ and phosphatidylcholine (PC) in the central nervous system (CNS) of mice. In consistence with the critical importance of Ca^2+^ and PC in the CNS, TPP2 gene ablation causes presenile dementia in female mice, which is closely associated with Ca^2+^/PC dysregulation-induced endoplasmic reticulum (ER) stress, abnormal autophagic degradation of CYP19A1 (aromatase), and estrogen depletion. This work therefore uncovers a new role of TPP2 in lipogenesis and neurosteroidogenesis which is tightly related to cognitive function of adult female mice.

**Conclusion:** Our study reveals a crucial role of TPP2 in controlling homeostasis of Ca^2+^ and lipids in CNS, and its deficiency causes sexual dimorphism in dementia. Thus, this study is not only of great significance for elucidating the pathogenesis of dementia and its futural treatment, but also for interpreting the role of TPP2 in other systems and their related disorders.

## Introduction

TPP2 is an evolutionally conserved serine peptidase which is ubiquitously expressed with varing levels in the cytoplasm of different eukaryotic cells 1-3. Its main role has been suggested to be protein degradation together with the proteasome 3, namely, TPP2 exerts its exopeptidase activity in extralysosomal peptide degradation 4. Studies in both humans and mice have shown that TPP2 activity is increased in tumour cells and by stress such as during starvation and muscle wasting 3. Whereas, TPP2 deficiency is linked to immunosenescence, lifespan regulation, human **i**ntellectual disability, and sterile brain inflammation mimicking multiple sclerosis (MS) 5-7. In addition to these observations, TPP2 has been reported to be involved in antigen presentation, cell growth, DNA repair, neuropeptide degradation, and cell death 8, 9. Interestingly, TPP2 also plays an important role in adiposity state of the body and its pro-adipogenic action seems to be independent of protease function, as catalytically inactive TPP2 also increases adipogenesis 2, 10.

As known, the brain has the second highest lipid content behind adipose tissue, and brain lipids constitute 50% of the brain dry weight 11. Thus, lipid metabolism/homeostasis is of particular importance for CNS. In fact, lipids and lipid intermediates are not only essential structural building blocks of the brain, but also critical signaling molecules and transmitters which may relay signals from the membrane to intracellular compartments or to other cells 12-14. Furthermore, an essential type of protein-lipid interaction within the hydrophobic plane is involved in targeting proteins to specific organelles, which can be used to modulate the structure, activity, and function of the integral membrane proteins and therefore regulate synaptic signalling in CNS 15, 16. In consistence with these observations, brain lipid dysregulation has been demonstrated to be associated with many neurological disorders, including bipolar disorders, schizophrenia, and neurodegenerative diseases such as Alzheimer's, Parkinson's, Niemann-Pick diseases and cerebral ischemic (stroke) injury 12, 13.

The major site of de novo structural lipid synthesis is the ER, from which the synthesized lipids are actively transported to the membranes of other organelles 17, 18. The lipid composition of different cellular organelles and even separate leaflets of the membrane bilayer differs both quantitatively and qualitatively 17, 19. Among these, PC and phosphatidylethanolamine (PE) are the most abundant glycerophospholipid species in mammalian cells 20. Lipids adopt defined phases dependent on their molecular structure and the physical conditions, and they tailor membrane identities and function as molecular hubs in all cellular processes 16, 19. Upon lipid dyshomeostasis and thereby ER membrane disequilibrium, namely the lipid bilayer stress (LBS), all three branches of unfolded protein response (UPR) (Inositol-requiring enzyme 1 (IRE1), PKR-like ER kinase (PERK) and Activating transcription factor 6 (ATF6)) can be activated, which monitor and buffer the lethal effects of altered ER homeostasis 21, 22. It is believed that IRE1 and PERK sense generalised LBS at the ER, while ATF6 is activated by an increase of specific sphingolipids 23. In addition, ER-associated protein degradation (ERAD) is essential for maintaining lipid homeostasis 24, 25, and it regulates the metabolism of many enzymes involved in lipid synthesis, degradation, and secretion 26, 27. In particular, disrupted PC synthesis causes both LBS-induced UPR and ERAD-associated LD dyshomeostasis 20, 28. However, it is still unclear how the de novo synthesis of PC is regulated, which is crucial for the function of the brain. Furthermore, although PC dysregulation-associated LBS can activate UPR and ERAD to buffer the imbalance and restore its homeostasis, it activates divergent transcriptional and non-transcriptional compensatory programms compared with proteotoxic stress. Even though autophagy was suggested to play an important role in maintaining cellular homeostasis during LBS-induced UPR 23, 28, the underlying mechanism is also unclear.

In this study, during investigation of the role of TPP2 in cognition and its related mechanism using TPP2 knockout cell lines and mice as tools, we found that TPP2 plays a key role in the de novo synthesis of PC in the CNS through regulation of Ca^2+^ homeostasis. Consistent with the critical importance of PC and Ca^2+^ in the CNS, we further found TPP2 depletion causes presenile dementia in female mice, which is closely associated with Ca^2+^ dyshomeostasis, PC deficit, and abnormal autophagic degradation of aromatase. In more details, we revealed that TPP2 is a key maintainer of cellular Ca^2+^ homeostasis via regulation of SERCA1 and IP3R1, and its depletion leads to imbalance of Ca^2+^ level which thereforth disrupts the enzymatic complexes for PC synthesis and causes PC deficit. The latter can in turn aggravate Ca^2+^ dyshomeostasis and further cause ER stress and ER stress-related autophagic degradation of aromatase, which ultimately results in estrogen depletion and dysfunction of adult neurogenesis and synaptogenesis.

## Results

### TPP2 depletion causes an increase of Ca^2+^ concentration inside ER and a decrease in cytosol, and these can be significantly reversed through ectopic expression not only of TPP2 but also of its enzymatic activity deficient mutants

**Figure 1:**
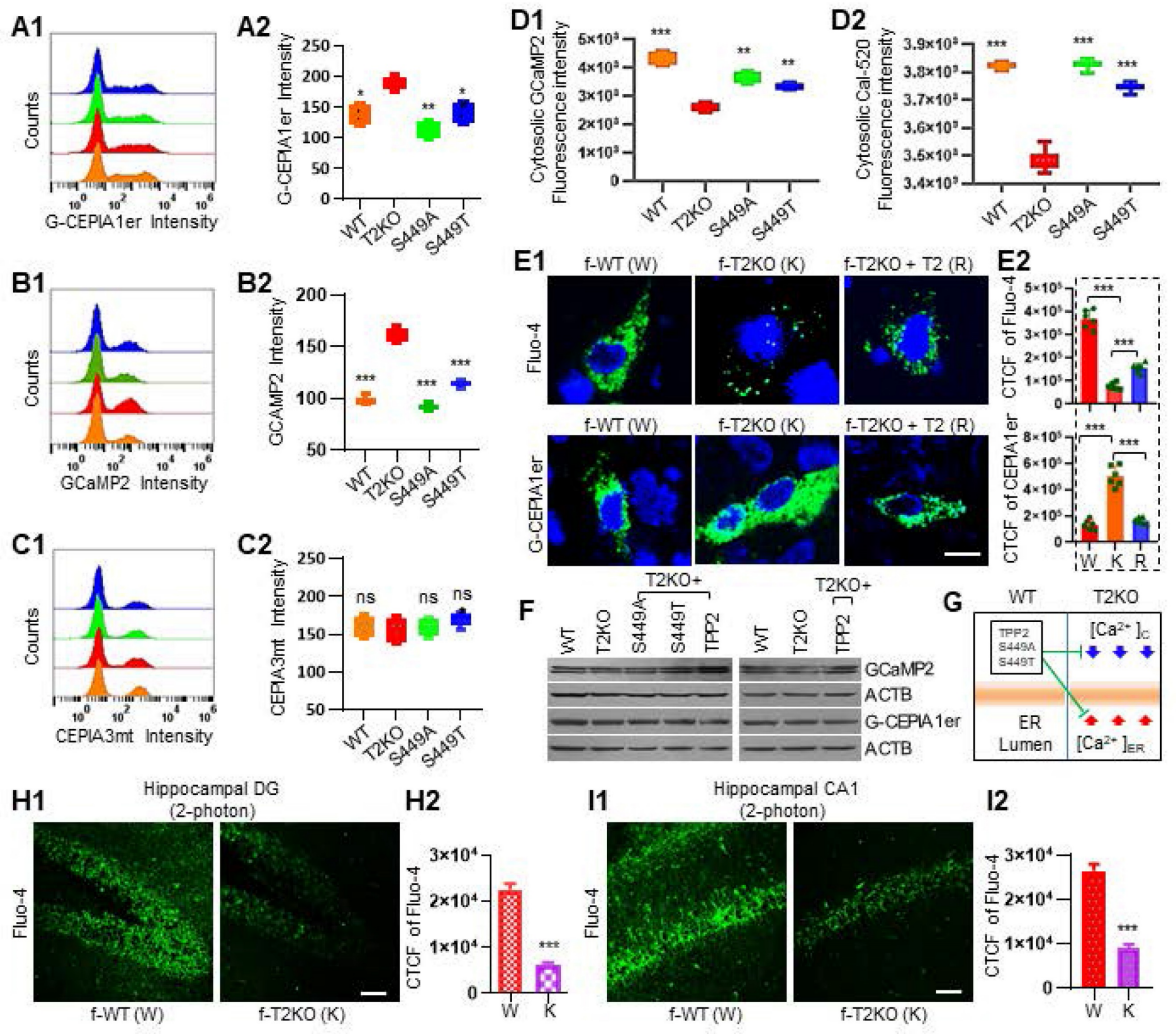
TPP2 depletion results in an increase of Ca^2+^ concentration inside ER along with a decrease in cytoplasm, both of which can be significantly reversed through ectopic expression not only of TPP2 but also of its enzymatic activity deficient mutant TPP2 S449A or S449T. (**A**) Flow cytometry analysis of the fluorescence response of the ER Ca^2+^ indicator G-CEPIA1er in 293T cells of different genetic background. (**A1**) An exemplary histogram plot of the G-CEPIA1er fluorescence intensity distribution in WT (Orange), T2KO (Red) and TPP2 S449A (Green) or S449T (Blue) expressing cells; TPP2 knockout 293T cells displays significantly enhanced fluorescence response of G-CEPIA1er as compared with WT and TPP2 S449A or S449T expressing counterpart cells. (**A2**) Statistical graph of the geometric means of G-CEPIA1er fluorescence intensity in different samples. n = 6, ** p ≤ 0.01, * p ≤ 0.05. WT: wild type; T2KO: TPP2 knockout; S449A: TPP2 S449A; S449T: TPP2 S449T. (**B**) Flow cytometry analysis of the fluorescence response of genetically encoded Ca^2+^ indicator GCaMP2 in 293T cells of different genetic background. (**B1**) An exemplary histogram plot of the GCaMP2 fluorescence intensity distribution in WT (Orange), TPP2 KO (T2KO, Red) and TPP2 S449A (Green) or S449T (Blue) expressing cells. TPP2 knockout cells displays significantly enhanced GCaMP2 fluorescence response as compared with WT and TPP2 S449A or S449T expressing counterpart cells. The enzymatic activity of the site-directed mutant TPP2 S449A or S449T was proved to be lost (**Figure 3C**). (**B2**) Statistical graph of the geometric means of GCaMP2 fluorescence intensity in different samples. n = 6, *** p ≤ 0.001. (**C**) Flow cytometry analysis of the fluorescence response of the mitochondrial Ca^2+^ indicator CEPIA3mt in 293T cells of different genetic background. (**C1**) An exemplary histogram plot of the CEPIA3mt fluorescence intensity distribution in WT (Orange), T2KO (Red) and TPP2 S449A (Green) or S449T (Blue) expressing cells; TPP2 knockout 293T cells displays nonsignificantly decreased fluorescence response of CEPIA3mt as compared with WT cells, TPP2 S449A or S449T expressing cells. (**C2**) Statistical graph of the geometric means of CEPIA3mt fluorescence intensity in different samples. n = 6, ** p ≤ 0.01, ^ns^ p > 0.05. (**D**) Cytosolic Ca^2+^ measurement on 293T cells of different genetic background using a fluorescence plate reader. (**D1**) Statistical graph of GCaMP2 fluorescence intensity in fractionated cytosol of different samples; (**D2**) Statistical graph of Cal-520 fluorescence intensity in fractionated cytosol of different samples. WT 293T (Orange), T2KO (Red) and TPP2 S449A (Green) or S449T (Blue). n = 6, *** p ≤ 0.001, ** p ≤ 0.01. These data showed that the cytosolic fluorescence response of TPP2 knockout cells is significantly decreased with either Ca^2+^ indicator Cal-520 or GCaMP2 as compared with that of WT and TPP2 S449A or S449T expressing counterpart cells. (**E**) Histochemical analysis of cytosolic and ER Ca^2+^ levels in WT and TPP2 knockout DIV10 female mouse hippocampal neurons. (**E1**) The fluorescence response of the cytosolic Ca^2+^ indicator Fluo-4 is significantly decreased, while that of G-CEPIA1er is significantly increased in TPP2 knockout neurons as compared with WT counterpart neurons, both of which can be significantly rescued by ectopic expression of TPP2 or TPP2 S449A/T (Data not shown). For this experiment, WT and TPP2 knockout neurons were prepared from 3 different batches of newborn littermate mice, and for each sample one representative scope from more than 30 confocal microscopic images is shown. (**E2**) Statistical graph of the corrected total cell fluorescence (CTCF). n = 6, *** p ≤ 0.001. Scale bars, 10 μm. f-WT: female WT, f-T2KO: female TPP2 KO. (**F**) Immunoblot for analysis of the ectopic expression of GCaMP2 and G-CEPIA1er in either 293T cells (**Left**) or DIV 10 mouse hippocampal neurons (**Right**). These data showed that the ectopic expression of GCaMP2 and G-CEPIA1er is not affected by TPP2 depletion or S449A/T mutation, suggesting that the observed difference of fluorescence response of GCaMP2 and G-CEPIA1er illustrated in (A)-(E) is solely based on different Ca^2+^ levels. (**G**) Sketch map showing the increase of Ca^2+^ concentration in ER along with its decrease in cytoplasm, which can be restored by TPP2 and S449A/T mutants. (**H**) and (**I**) 2-photon imaging using Fluo-4 indicator showing the decrease of Ca^2+^ concentration in cytosol of TPP2 knockout DG and CA1 neurons. (**H1**) and (**I1**) Representative images of Ca^2+^ in DG or CA1 regions. (**H2**) and (**I2**) Statistical graph of CTCF of the images. n=4, *** p ≤ 0.001. Scale bars, 50 μm. f-WT: female WT, f-T2KO: female TPP2 KO.

Ca^2+^ is one of the most versatile and universal signaling agents in the human body, it regulates many cellular and molecular processes 29. Given that TPP2 also has multiple functions as abovementioned, we first examined its regulatory effect on Ca^2+^. We used small molecular Ca^2+^-sensitive dyes and genetically encoded Ca^2+^ indicators (GECIs) to detect the concentration of Ca^2+^ in the cytosol, ER, and mitochondria of TPP2 knockout 293T cell lines and hippocampal neurons (Figure 1A-E). Based on flow cytometry analysis of the fluorescence response of the GECIs (GCaMP2, G-CEPIA1er, and CEPIA3mt) in 293T cells with different genetic background, we found that the ER-targeted Ca^2+^ probe G-CEPIA1er displays significantly enhanced fluorescence response in TPP2 knockout cells as compared with wild type (WT) and TPP2 S449A or S449T expressing counterpart cells (Figure 1A). By contrast, the mitochondria-targeted probe CEPIA3mt displays a decreased fluorescence response, although not significantly, in TPP2 knockout cells compared with control counterpart cells (Figure 1C). Interestingly, although GCaMP2 displays significantly enhanced fluorescence response in TPP2 knockout cells as compared with control counterpart cells (Figure 1B), its fluorescence intensity in fractionated cytosol is significantly lower than that in control samples (Figure 1D1). In contrast, its fluorescence intensity in fractionated ER is significantly higher than that in control samples (not shown) with the absolute intensity value being much higher than the value in the cytosol. This suggests that GCaMP2 is mainly targeted to ER which plays a central role in Ca^2+^ storage. In consistence with these observations, the Cal-520 fluorescence intensity in fractionated cytosol of TPP2 knockout 293T cells is also significantly lower than that in control samples (Figure 1D2). Furthermore, histochemical analysis of Ca^2+^ levels in DIV10 hippocampal neurons of female WT and TPP2 knockout mice showed that the fluorescence response of the cytosolic Ca^2+^ indicator Fluo-4 is significantly decreased, while the response of ER Ca^2+^ probe G-CEPIA1er is significantly increased in TPP2 knockout neurons as compared with WT counterpart neurons (Figure 1E1 Left and middle columns). In particular, both reduced response to Fluo-4 and increased response to G-CEPIA1er in TPP2 knockout neurons can be saved by ectopic expression of TPP2 (Figure 1E1 Right column) or TPP2 S449A/T (Data not shown). The same experiment has shown that there is no significant difference in hippocampal neurons between WT and TPP2 knockout male mice (Data not shown). To rule out the possibility that the differential responses to these GECIs in TPP2 knockout cells or neurons from female mice may be attributed to different expression levels of these GECIs in cells or neurons with different genetic backgrounds, Western blotting was performed and showed that their expression did not significantly change under TPP2 depletion as compared with WT genotype (Figure 1F). In addition, two-photon imaging of neurons in both hippocampal DG and CA1 regions has shown that the in situ Fluo-4 labelled Ca^2+^ levels in hippocampal neurons of 6 month-old TPP2 knockout female mice are significantly lower than WT controls (Figure 1H-I). Thus, TPP2 plays a key role in maintaining intracellular Ca^2+^ homeostasis, especially the Ca^2+^ levels in Cytosol and inside ER, which are involved in many biological processes. Crucially, the enzymatic activity deficient mutants of TPP2 retain the function of maintaining Ca^2+^ balance (Figure 1E and G).

### TPP2 interacts with IP3R1, VDAC2, SERCA1, ATF6, IRE1 and PERK, and TPP2 depletion disrupts the integrity of MAMs and the inhibitory interaction between SERCA1 and phospholamban (PLN) and between IP3R1 and calreticulin (CALR), which can be reversed by ectopic expression of TPP2 and its enzymatic activity deficient mutants

**Figure 2:**
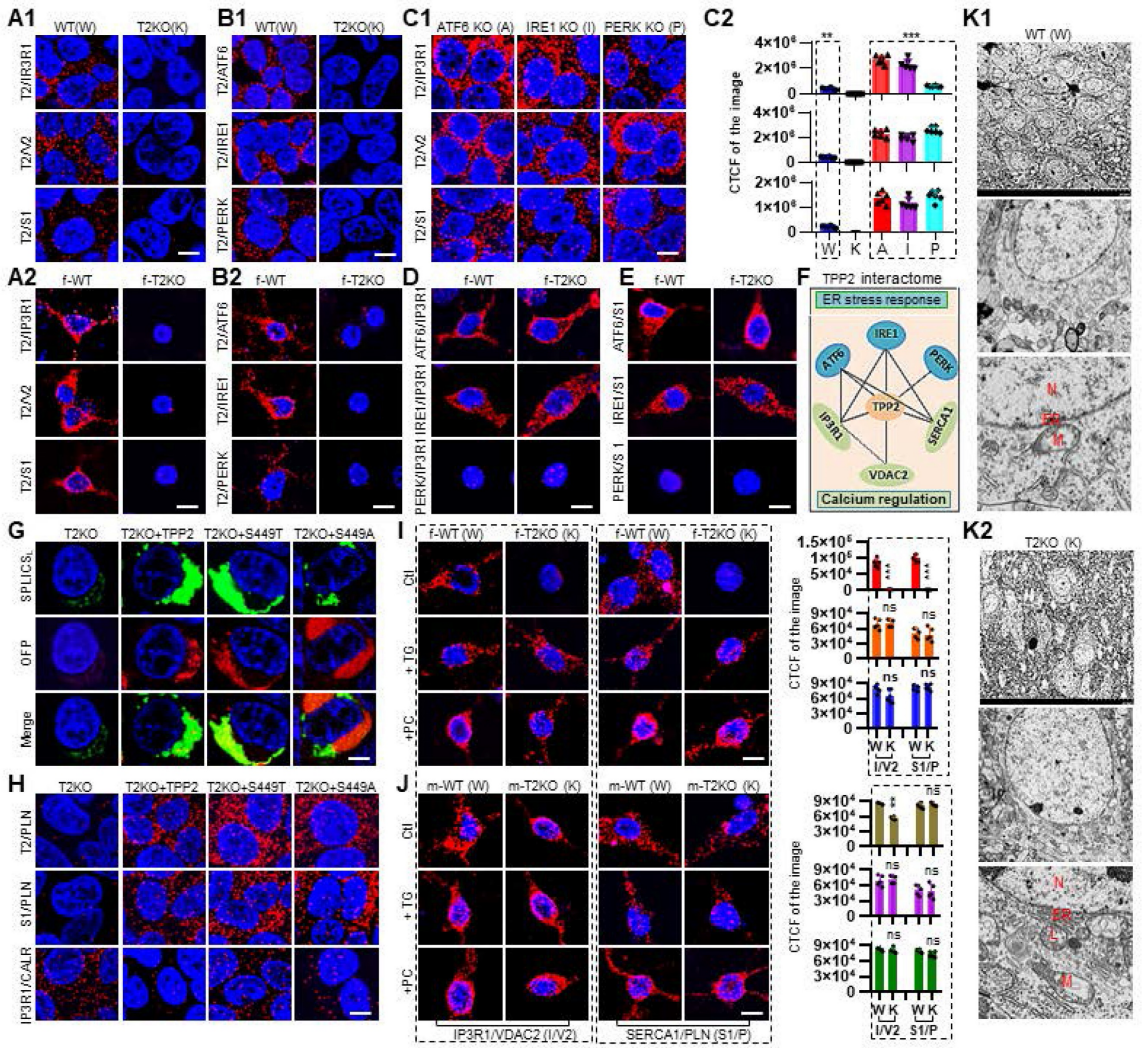
TPP2 interacts with IP3R1, VDAC2, SERCA1, ATF6, IRE1 and PERK, and TPP2 depletion disrupts the integrity of MAMs and the inhibitory interaction between SERCA1 and PLN, these can be reversed by ectopic expression of TPP2 and its enzymatic activity deficient mutants. (**A**) In WT 293T cells (**A1,** left column) and neurons (**A2**, left column), PLA showing the physical interactions between TPP2 and IP3R1, VDAC2 and SERCA1. By contrast, in TPP2 knockout 293T cells (**A1**, right column) and neurons (**A2**, right column) the PLA fluorescence signals are nearly completely abolished. For each pair of PPI between TPP2 and IP3R1, VDAC2 and SERCA1, one representative scope from more than 30 confocal microscopic images is shown. This experiment was repeated for 3 times, WT and TPP2 knockout neurons were prepared from 3 different batches of newborn littermate mice. DIV 10 neurons from male (m) and female (f) mice showed similar results. Data from female mice are shown here. Scale bars, 10 μm. f-WT: female WT, f-T2KO: female TPP2 KO. (**B**) In WT 293T cells (**B1**, left column) or neurons (**B2**, left column), PLA showing the physical interaction between TPP2 and ATF6, IRE1 and PERK. By contrast, in TPP2 knockout 293T cells (**B1**, right column) and neurons (**B2**, right column) the PLA fluorescence signals are nearly completely abolished. For each pair of PPI between TPP2 and ATF6, IRE1 and PERK, one representative scope from more than 30 confocal microscopic images is shown. This experiment was repeated for 3 times, WT and TPP2 knockout neurons were prepared from 3 different batches of newborn littermate mice. DIV 10 neurons from male and female mice showed similar results. Data from female mice are shown here. Scale bars, 10 μm. f-WT: female WT, f-T2KO: female TPP2 KO. (**C**) (**C1**) The PPIs between TPP2 and IP3R1, VDAC2 and SERCA1 are significantly enhanced in ATF6, IRE1 or PERK knockout cells as compared with that in WT cells (shown in A1, left column). For each pair of PPI between TPP2 and IP3R1, VDAC2 and SERCA1, one representative scope from more than 20 confocal microscopic images is shown. Scale bars, 10 μm. (**C2**) Statitical graph of the CTCF value of all pairs of PPI between TPP2 and IP3R1, VDAC2 and SERCA1 in WT, TPP2 knockout (A1 right column), ER stress sensor knockout cell lines. n =6, *** p ≤ 0.001, ** p ≤ 0.01. (**D**) In both WT and TPP2 knockout neurons, IP3R1 interacts with ATF6 and IRE1 while no interaction between IP3R1 and PERK. For this experiment, WT and TPP2 knockout neurons were prepared from 3 different batches of neonatal littermate mice, and for each pair of PPI between IP3R1 and ATF6, IRE1 and PERK, one representative scope from more than 20 confocal microscopic images is shown. Neurons from male and female mice showed similar results. Data from female mice are shown. Scale bars, 10 μm. f-WT: female WT, f-T2KO: female TPP2 KO. (**E**) In both WT and TPP2 knockout neurons, SERCA1 interacts with ATF6 and IRE1 while no interaction between SERCA1 and PERK. For this experiment, WT and TPP2 knockout neurons were prepared from 3 different batches of neonatal littermate mice, and for each pair of PPI between SERCA1 and ATF6, IRE1 and PERK, one representative scope from more than 20 confocal microscopic images is shown. Neurons from male and female mice showed similar results. Data from female mice are shown. Scale bars, 10 μm. f-WT: female WT, f-T2KO: female TPP2 KO. (**F**) Schematic diagram of the TPP2 interaction partners and their connection network showing that TPP2 organize a network which is composed of components responsible for Ca^2+^ regulation and ER stress response. (**G**) Measurement of the MAMs by split-GFP-based contact site sensors (SPLICS) in 293T cells under different conditions. Representative images of TPP2 knockout cells expressing the SPLICS for wide heterotypic organelle juxtaposition (SPLICS_L_) with or without co-expression of TPP2, TPP2 S449T or TPP2 S449A fused to an orange fluorescent protein (OFP) reporter. TPP2 depletion causes disruption of MAMs structure which can be restored by ectopic expression of TPP2 and TPP2 S449T or S449T. This experiment was repeated for 3 times. Each of the presented scope is from more than 20 confocal microscopic images. Scale bars, 5 μm. These data showed that TPP2 is crucial for maintenance of MAMs integrity in 293T cells. (**H**) At the absence of TPP2, both the inhibitory interactions of SERCA1 (S1)/Phospholamban (PLN) and IP3R1/Calreticulin (CALR) are disrupted and this can be reversed by ectopic expression of TPP2 and its enzymatic activity deficient mutants (TPP2 S449A or T). In paricular, TPP2 interacts with both SERCA1 and PLN, and TPP2 depletion abolishes the interaction between SERCA1 and PLN, indicating that TPP2 plays a critical role in maintaining cytosolic Ca^2+^ homeostasis by regulation of ER Ca^2+^ refilling via SERCA1. This experiment was repeated for 3 times. Each of the presented scope is from more than 30 confocal microscopic images. Scale bars, 10 μm. (**I**) (**Left**) IP3R1 interacts with VDAC2 in WT neurons, whereas this interaction is lost in TPP2 knockout neurons. Similarly, in TPP2 knockout neurons the inhibitory interaction of SERCA1/PLN is lost too. In addition, the lost interactions between IP3R1 and VDAC2 and between SERCA1 and PLN in DIV 10 TPP2 knockout neurons can be significantly reversed by transient treatment with TG and PC (TG treatment: 5 μM for 5 h at 37°C, PC treatment: 5 μM for 24 h at 37°C). For this experiment, WT and TPP2 knockout neurons were prepared from 3 different batches of newborn littermate mice, and for each pair of PPI under different conditions one representative scope from more than 30 confocal microscopic images is shown. Scale bars, 10 μm. (**Right**) Statistical graph of the CTCF values of selected scopes of IP3R1/VDAC2 or SERCA1/PLN from independent experiments. n =6, *** p ≤ 0.001, **^ns^** p > 0.05. f-WT: female WT, f-T2KO: female TPP2 KO. (**J**) (**Left**) Under TPP2 depletion, although the interaction between IP3R1 and VDAC2 is reduced in neurons of male mice, it is greatly inferior to that in neurons of female mice, meanwhile the interaction between SERCA1 and PLN is nearly unaffected. For this experiment, WT and TPP2 knockout neurons were prepared from 3 different batches of newborn littermate mice, and for each pair of PPI under different conditions one representative scope from more than 30 confocal microscopic images is shown. Scale bars, 10 μm. (**Right**) Statistical graph of the CTCF values of selected scopes of IP3R1/VDAC2 or SERCA1/PLN from independent experiments. n =6, *** p ≤ 0.001, **^ns^** p > 0.05. m-WT: male WT, m-T2KO: male TPP2 KO. (**K**) TPP2 depletion destroys the micromorphology and subcellular organs of hippocampal neurons in 6 month-old TPP2 knockout female mice. The representative images from 3 samples in each group are shown. (**K1**) In WT group, most hippocampal neurons exhibit normal morphology with intact organelles. (**K2**) In TPP2 knockout group, many hippocampal neurons show abnormal or damaged morphology (upper). The neurons that still have identifiable morphology show nuclear enlargement (middle), increased volume of endoplasmic reticulum, deformed mitochondria, deformed MAM, and lysosome proliferation (lower). N: Nuclear; ER: Endoplasmic Reticulum; M: Mitochondria; L: Lysosome.

In view of the regulatory effect of TPP2 on the level of Ca^2+^ in the cytosol, ER, and mitochondria, we have systematically screened the interacting relationship between TPP2 and Ca^2+^ regulation-related molecules, especially those ones which are located in ER and mitochondrial membranes, through PLA 30-37. Fortuitously, we found that TPP2 can interact in situ with several Ca^2+^ regulators, including inositol 1,4,5-triphosphate receptor (IP3R1), voltage dependent anion-selective channel 2 (VDAC2), sarcoendoplasmic reticulum Ca^2+^ ATPase (SERCA1), ATF6, IRE1, and PERK (Figure 2A-B). Among them, IP3R1, VDAC2, and SERCA1 are classical Ca^2+^ regulator 30-34, while the other three proteins are classical ER stress sensors, but also participate in Ca^2+^ regulation 35-37. We found that the protein-protein interactions (PPIs) between TPP2 and IP3R1, VDAC2 or SERCA1 are significantly enhanced in ER stress sensor knockout cells as compared with WT cells (Figure 2A1, C1-C2). These results indicate that there is competition for TPP2 binding between ER stress sensors and IP3R1, VDAC2, and SERCA1. In order to determine whether ATF6, IRE1, and PERK are involved in TPP2 depletion-induced Ca^2+^ dyshomeostasis, we prepared TPP2 and ER stress sensor double knockout cell lines. Interestingly, depletion of any of the three ER stress sensors neither aggravates nor alleviates TPP2 knockout-induced Ca^2+^ dyshomeostasis (Data not shown). Furthermore, under TPP2 depletion, the association among these ER-localized TPP2 interacting partners (ATF6, IRE1, IP3R1, and SERCA1) has no change as compared with WT controls (Figure 2D-E), suggesting that the interactions between these molecules are independent of TPP2, although all of them can interact with TPP2. However, for unknown reason, we didn't find the interaction between PERK and IP3R1 or SERCA1 (Figure 2D-E), although all of them are TPP2 interaction partners as abovementioned (Figure 2B1-B2).

Given that IP3R1 releases Ca^2+^ from ER 30, SERCA1 transports Ca^2+^ from the cytoplasm into ER 34, while VDAC2 mediates mitochondrial Ca^2+^ uptake 38, and all of them have been found to be enriched in MAMs 39-42, we next performed measurement of the MAMs in TPP2 knockout 293T cells using split-GFP-based contact site sensors (SPLICS). As expected, TPP2 depletion causes disruption of MAMs structure and this can be restored by ectopic expression of TPP2 and TPP2 S449T or S449T (Figure 2G). Consistent with this finding, the interaction of IP3R1 and VDAC2 is disrupted in TPP2 knockout neurons (Figure 2I). These results may explain why the mitochondrial Ca^2+^ content becomes less, although not significantly, under TPP2 depletion. To identify the roles of IP3R1 and SERCA1 in TPP2 depletion-induced cytosolic Ca^2+^ decrease and ER Ca^2+^ increase, we examined the switch status of IP3R1 and SERCA1 through their gatekeepers namely CALR and PLN, respectively. In agreement with the fact that CALR inhibits the ER Ca^2+^ release through IP3R1 35, 43-45 and that PLN inhibits the ER Ca^2+^ refilling 46, 47, we revealed that the interaction of SERCA1 and PLN is almost completely abolished, whereas the interaction between IP3R1 and CALR is enhanced in TPP2 knockout 293T cells and hippocampal neurons of female mice, and both of which can be prevented by ectopic expression of TPP2 and its enzymatic activity deficient mutants (Figure 2H-I). These results suggest that TPP2 depletion-induced Ca^2+^ dyshomeostasis are caused by combined effect of uncontrolled ER Ca^2+^ refilling through SERCA1 and aberrantly inhibited ER Ca^2+^ release through IP3R1. By contrast, under TPP2 depletion, although the interaction between IP3R1 and VDAC2 is reduced in neurons of male mice, it is greatly inferior to that in female mice, meanwhile the interaction between SERCA1 and PLN is nearly unaffected (Figure 2J). In order to explore how TPP2 physiologically regulate Ca^2+^ homeostasis, we treated DIV 10 hippocampal neurons with BAPTA to downregulate cytosolic Ca^2+^ level mimicking physiological conditions. Our data showed that TPP2 directly mediates SERCA1/PLN and prevents IP3R1/CALR interactions at presence of BAPTA to counteract cytosolic Ca^2+^ decrease, while TPP2 depletion completely abolished these effects (Figure S1A ). In consistence with above findings, our electron microscopic data showed that TPP2 depletion destroys the micromorphology and subcellular organs of hippocampal neurons in 6 month-old TPP2 knockout female mice. In TPP2 knockout group, many hippocampal neurons show abnormal or damaged morphology (Figure 2K1). The neurons that still have identifiable morphology show nuclear enlargement, increased volume of endoplasmic reticulum, deformed mitochondria, deformed MAM, and lysosome proliferation (Figure 2K2). Altogether, these observations showed that TPP2 depletion causes dimorphism in Ca^2+^ dyshomeostasis in hippocampal neurons of male and female mice.

### Co-IP proves that TPP2 interacts with IP3R1, VDAC2, SERCA1, ATF6, IRE1, and PERK, and that they are attributed to the conserved C tail rather than peptidase domain of TPP2

**Figure 3:**
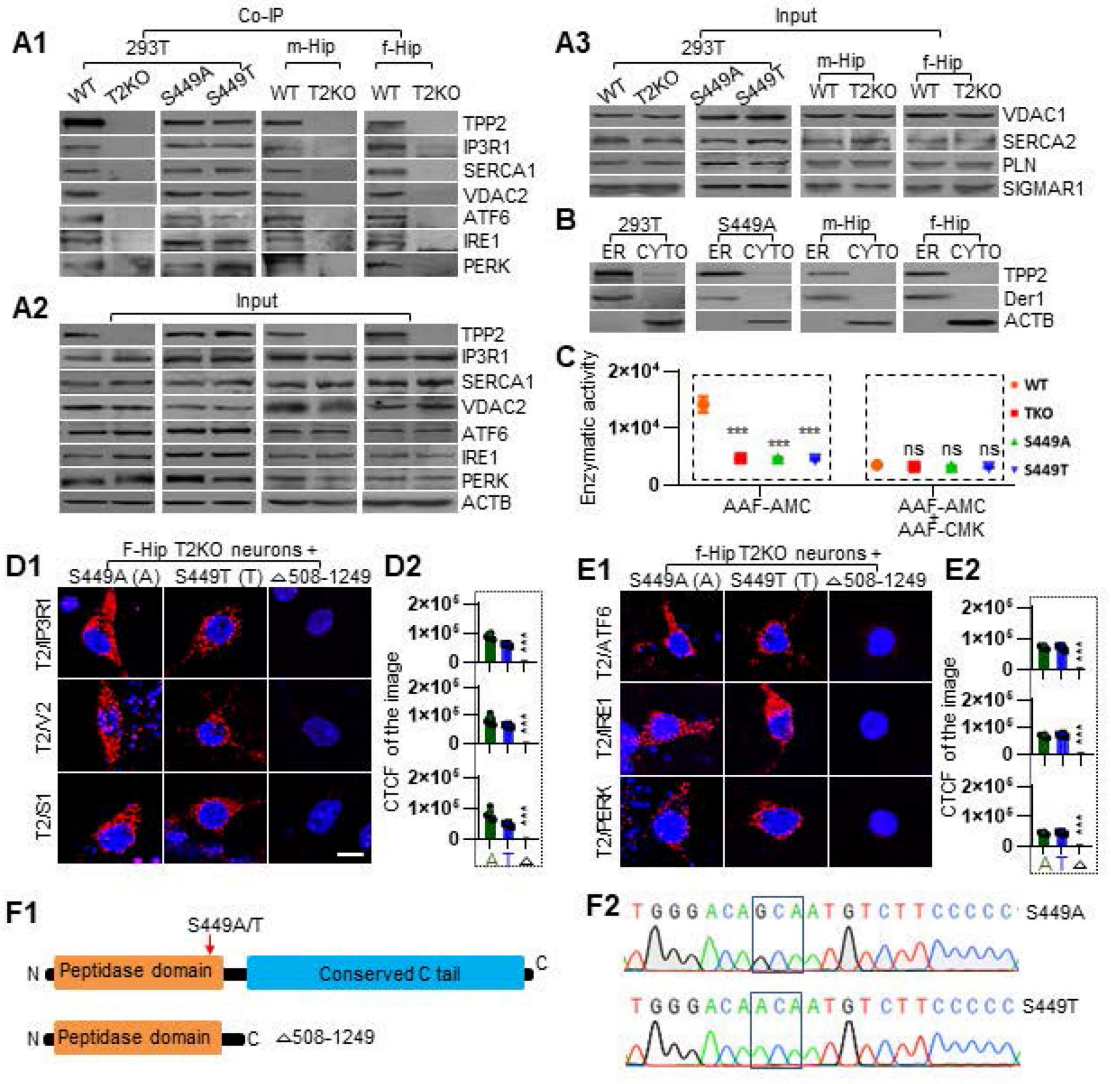
Co-IP proves that TPP2 interacts with IP3R1, VDAC2, SERCA1, ATF6, IRE1, and PERK, and that they are attributed to the conserved C tail rather than peptidase domain of TPP2. (**A**) Co-IP combined with immunoblot proved that (**A1**) TPP2 and its S449A/T mutants physically interact with IP3R1, VDAC2, SERCA1, ATF6, IRE1, and PERK in both 293T cells and mouse hippocampus (P30), while (**A2**) there is no significant change in the expression levels of these interaction partners at the absence of TPP2. (**A3**) In addition, the expression levels of other related proteins, including SERCA2, VDAC1, SIGMAR, and PLN, did not show significant change in the cellular lysates or hippocampal homogenates. The representative result of three repeated experiments is shown. (**B**) Subcellular fractionation assay for analysis of TPP2 distribution in ER membrane. After fractionation, western blot results showed that TPP2 mainly appears in ER component together with Derlin-1 (Der1) rather than in cytoplasm of 293T cells and cytoplasm of mouse hippocampal neural cells, suggesting that TPP2 is enriched in the ER compartment. One representative result of three repeated experiments is shown. (**C**) Enzymatic activity assay of cellular lysates prepared from 293T cells expressing wild type TPP2 or its S449A/T mutants using TPP2 knockout 293T cell lysate as negative control. The enzymatic activity was measured on cellular lysates containing 5 μg total proteins for each sample at presence of 100 μM AAF-AMC as substrate with or w/o 50 μM AAF-CMK as inhibitor in 200 μL reaction volume. The data shows that the enzymatic activities of TPP2 S449A and S449T mutants decrease to the level comparable to that under TPP2 depletion, and the enzymatic activity of TPP2 on AAF-AMC can be completely inhibited by AAF-CMK. The representative result of 6 repeated experiments is shown. (**D**) PLA for analysis of the PPI between TPP2 site-directed mutants and IP3R1, VDAC2 or SERCA1 in TPP2 knockout mouse hippocampal neurons ectopically expressing TPP2 S449A or S449T mutant. (**D1**) Both TPP2 S449A and S449T interact with IP3R1, VDAC2 and SERCA1, whereas the C terminal domain-deleted mutant is deficient in interaction with these proteins. For each pair of PPI, one representative scope from more than 30 confocal microscopic images is shown. Scale bars, 10 μm. (**D2**) Statistical graph of the CTCF values of all the selected scopes of PPI between TPP2 mutants and IP3R1, VDAC2 or SERCA1 shown in D1. For each sample 6 representative scopes are selected for statistics. (**E**) PLA to detect the PPI between TPP2 site-directed mutants and ATF6, IRE1 and PERK in TPP2 knockout mouse hippocampal neurons ectopically expressing TPP2 S449A or S449T mutant. (**E1**) TPP2 S449A and S449T interacts with ATF6, IRE1 and PERK, whereas the C terminal domain-deleted mutant is deficient in interaction with these proteins. For each pair of PPI, one representative scope from more than 30 confocal microscopic images is shown. Scale bars, 10 μm. (**E2**) Statistical graph of the CTCF values of all of the selected scopes of PPI between TPP2 mutants and ATF6, IRE1 and PERK shown in E1. For each sample 6 representative scopes are selected for statistics. (**F1**) Illustration of the TPP2 molecular domains and the constructs established for using in this study. (**F2**) Sequencing results of the established site-directed mutations of TPP2 gene.

To verify the interactions between TPP2 and its interaction partners as abovementioned, we performed further Co-IP. The results demonstrated that TPP2 and its S449A or S449T mutant have physical interactions with all the 6 interaction partners determined by PLA in 293T cells and mouse hippocampus, while there is no significant change in the expression levels of these interaction partners at the absence of TPP2 (Figure 3A1-A2). In addition, the expression levels of other related proteins, including SERCA2, VDAC1, SIGMAR, and PLN, did not show significant change in TPP2 knockout 293T cells and hippocampi (Figure 3A3). Furthermore, we performed subcellular fractionation assay to determine TPP2 distribution within cells. Our results showed that TPP2 mainly exists in ER component characterized by derlin-1 (Der1) rather than in cytosol of 293T cells and mouse hippocampal neural cells (Figure 3B). This is in agreement with the fact that, except VDAC2, which is located in the outer mitochondrial membrane (OMM) of MAMs, all the other interaction partners of TPP2 are located in ER membrane. Considering that the site-directed mutations of TPP2 (S449A and S449T) don't interfere with its interaction with the interaction partners (Figure 3A1), we prepared truncated TPP2 mutant with deletion of its conserved C domain (AA 508-1249) (Figure 3F1) and found that the truncated form of TPP2 completely loses its interaction with all the 6 interaction partners of WT TPP2 (Figure 3D-E). Moreover, although it is known that S449 is involved in the peptidase activity of TPP2, it is unclear to which extent the site mutations of S449A and S449T will interfere the catalytic activity of TPP2. Thus, we conducted enzymatic activity assay of cellular lysates prepared from 293T cells expressing WT TPP2, S449A, or S449T mutant, using TPP2 knockout 293 T cell lysate as negative control. The data showed that the enzymatic activities of TPP2 S449A and S449T mutants decrease to the level comparable to that under TPP2 depletion, and the enzymatic activity of TPP2 on AAF-AMC can be completely inhibited by AAF-CMK (Figure 3C). To verify the site mutations, the sequencing results of the constructs for expressing TPP2 S449A and S449T are displayed (Figure 3F2).

### TPP2 depletion prevents the assembly of catalytic enzyme complex for PC synthesis on the ER membrane, and this can be reversed by increasing the concentration of cytosolic Ca^2+^

**Figure 4:**
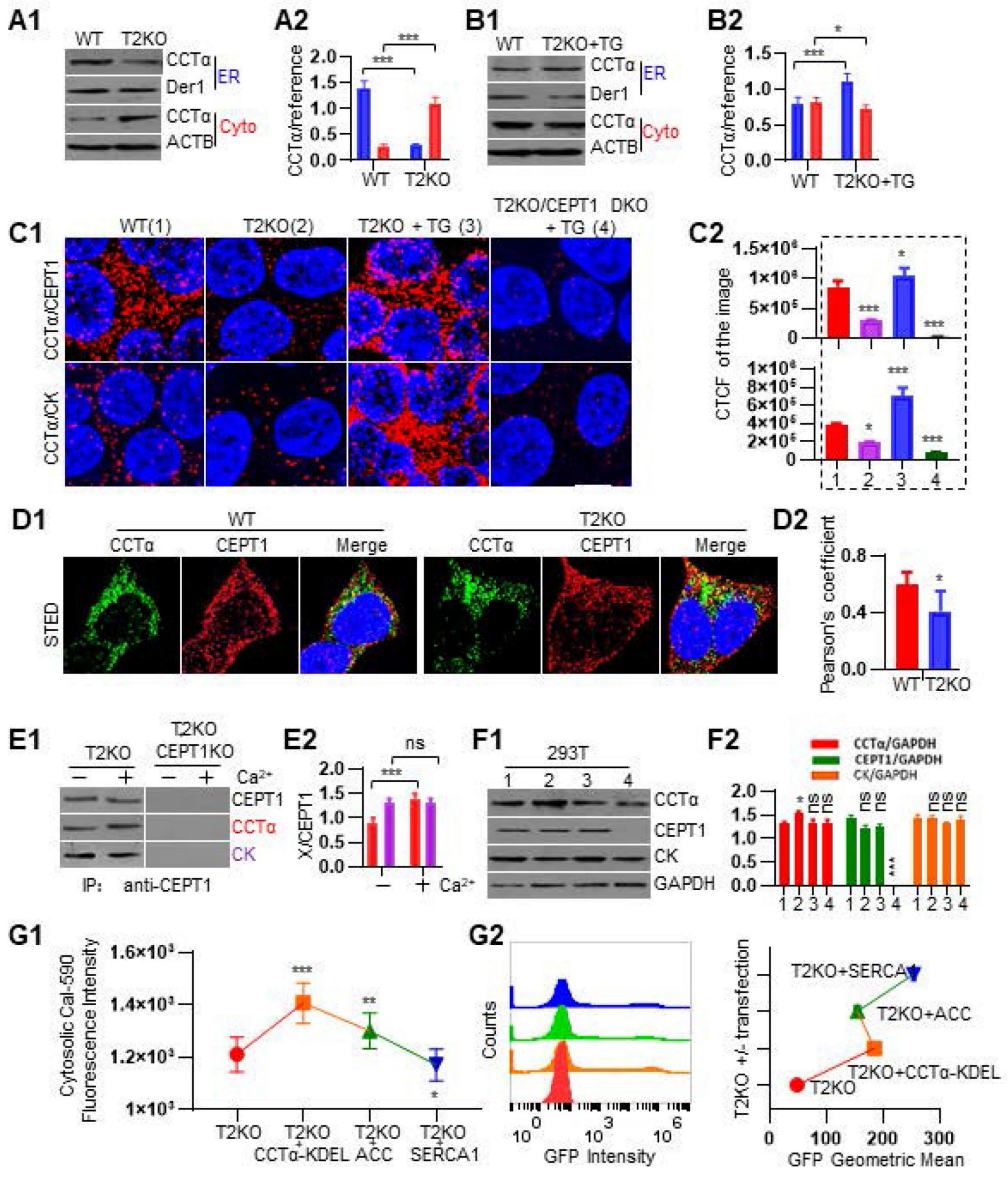
TPP2 depletion prevents the assembly of catalytic enzyme complex for PC synthesis on the ER membrane, and this can be reversed by increasing the concentration of cytoplasmic Ca^2+^. (**A**) Subcellular fractionation assay to examine CCTα distribution in 293T cells without TG treatment. (**A1**) One representative western blot result of CCTα in ER membrane and cytoplasm of 293T cells. (**A2**) Quantitative analysis of western blot results from 3 repeated experiments showed that CCTα mainly appears in cytoplasm of 293T cells without TG treatment. (**B**) Subcellular fractionation assay to determine CCTα distribution in 293T cells with transient TG treatment. (**B1**) One representative western blot result of CCTα in ER membrane and cytoplasm of 293T cells with transient TG treatment. (**B2**) Quantitative analysis of western blot results from 3 times repeats showed that CCTα mainly appears in ER membrane after TG treatment. (**C**) PLA to detect the coupling of CCTα with CEPT1 and CK. (**C1**) CCTα is coupled with CEPT1 and CK in WT 293T cells, and at the absence of TPP2 the interactions between CCTα and CEPT1, as well as CCTα and CK, becomes significantly reduced, which can recover or even increase to a higher level than that in WT cells by upregulating the level of cytosolic Ca^2+^ through transient treatment with TG. In addition, the interaction of CCTα with CK becomes reduced under CEPT1 depletion. For each pair of PPI, one representative scope from more than 30 confocal microscopic images is shown. Scale bars, 10 μm. TG treatment: 5 μM for 5 h at 37°C. (**C2**) Statistical graph of the CTCF values of all the selected scopes of PPI pairs shown in C1. For each sample 6 representative scopes are selected for statistics. (**D**) Representative STED images of CCTα and CEPT1 double fluorescent staining (**D1**) and statistical graph of colocalization analysis (**D2**), showing that the coupling of CCTα with CEPT1 is significantly decreased under TPP2 depletion. (**E**) Co-IP combined with immunoblot for analysis of the interaction between CEPT1, CCTα and CK. (**E1**) CCTα and CK are co-immunoprecipitated with CEPT1. However, in the presence of Ca^2+^, CCTα is more coprecipitated than in the absence of Ca^2+^, whereas the amount of coprecipitated CK remains unchanged with or without addition of Ca^2+^. By contrast, CEPT1 ablation completely abolishes the Co-IP. (**E2**) Quantitative analysis of western blot results from 3 times repeats showed that the interaction between CEPT1 and CCTα becomes significantly increased at presence of 1 mM Ca^2+^, whereas the interaction between CEPT1 and CK remains unchanged. (**F**) Immunoblot for analysis of the expression levels of CCTα, CEPT1 and CK in 293T cells. (**F1**) One representative western blot result of the expression levels of CCTα, CK and CEPT1 in cells with different genetic backgrounds and/or under different conditions. 1: WT; 2: TPP2 KO; 3: TPP2 KO + TG; 4: TPP2/CEPT1 DKO + TG. (**F2**) Quantitative analysis of western blot results from 3 repeated experiments showed that the expression levels of CK and CEPT1 are not significantly altered, while CCTα in TPP2 knockout cells becomes clearly higher than control. (**G**) Cal-590 staining assay to determine cytosolic Ca^2+^ in TPP2 knockout 293T cells with and w/o ectopic expression of CCTα-KDEL, ACC, and SERCA1 fused to EGFP. (**G1**) Ectopic expression of CCTα-KDEL or ACC increases cytosolic Ca^2+^ level. By contrast, ectopic expression of SERCA1 decreases cytosolic Ca^2+^ level. (**G2**) Flow cytometry assay for analysis of the ectopic expression of CCTα-KDEL, ACC, and SERCA1 fused to EGFP.

Ca^2+^ and lipid metabolism mutually regulate each other 48, 49. For example, incubation of rat heptocytes with millimolar scale of Ca^2+^ in the medium can stimulate CTP:phosphocholine cytidylyltransferase (CCT) to translocate from cytosol to microsomes 50. There are two mammalian CCT enzymes, namely ubiquitously expressed CCTα and tissue restricted CCTß 51, 52. The two CCTs have highly similar catalytic and membrane-binding (M) domains but differ at their N and C-terminal disordered regions 53. It is known that the translocation of CCTα from cytosol to membrane can activate its enzymatic activity for CDP-choline synthesis 54-56. ER is the central compartment for PC biogenesis, while CCTα is the dominant form of CCT for PC biosynthesis and LD biogenesis 17, 56, 57. Additionally, CCTα shuttles between nucleus and cytoplasm and CDP-choline is synthesized primarily by extranuclear CCTα following its translocation to the ER 51, 58. However, it is unclear how CCT activity is regulated by endogenous Ca^2+^. Given that TPP2 depletion causes a decrease of Ca^2+^ concentration in cytosol and an increase inside ER, we first examined the ER membrane-bound CCTα in TPP2 knockout cells and WT control cells. We performed subcellular fractionation assay and found that the amount of CCTα in ER component of TPP2 knockout cells is significantly reduced as compared with WT cells, whereas the amount of CCTα in cytosol of TPP2 knockout cells is exactly the opposite of ER components as compared with WT cells (Figure 4A). In particular, transient treatment of TPP2 knockout cells with Thapsigargin (TG), a SERCA inhibitor to prevent ER Ca^2+^ refilling 59, can strongly promote translocation of CCTα from cytosol to ER membrane and invert its distribution in cytosol and ER component of TPP2 knockout cells (Figure 4B).

PC is synthesized almost exclusively through the CDP-choline pathway in essentially all mammalian cells 51, 60. In the CDP-choline pathway, choline kinase (CK) is the first rate-limiting enzyme, which catalyzes choline phosphorylation to yield phosphocholine and is usually located in cytosol 61-63. CCTα is the second rate-limiting enzyme, which catalyzes the synthesis of CDP-choline and resides in the cytosol, nucleus, and on ER membrane 52, 64, 65. While choline-ethanolamine phosphotransferase 1 (CEPT1) and choline phosphotransferase 1 (CPT1) catalyze the last step of PC biosynthesis and reside in different cell organelles 60, 66-68. By comparison, CEPT1 is an ER transmembrane protein and is responsible for catalyzing biosynthesis of most PC and PE 57, 66, while CPT1 resides in the trans-Golgi network (TGN) and is more inclined to synthesize PC with polyunsaturated fatty acid (PUFA) chains, which has been proved to be required for autophagosome membrane formation and maintenance during autophagy 66, 69, 70. As such, to further validate CCTα less distribution on ER membrane of TPP2 knockout cells and uncover whether the enzymatic complexes for PC biosynthesis are also less assembled under TPP2 depletion, we next compared the interactions between CK, CCTα, and CEPT1 using PLA. Our results showed that CCTα is more coupled with CEPT1 and CK in WT 293T cells, and at the absence of TPP2 the interaction between CCTα and CEPT1, as well as interaction between CCTα and CK, becomes significantly reduced (Figure 4C-D). Also, transient treatment with TG can reverse or even upgrade their interactions to a higher level as compared with WT cells, while CEPT1 depletion almost completely eliminates the TG effect (Figure 4C). Based on these results, we further investigated the assembly of enzymatic complexes by Co-IP. In consistence with PLA results, CCTα and CK are co-immunoprecipited with CEPT1 (Figure 4E). Interestingly, in the presence of Ca^2+^ CCTα is more precipitated than without Ca^2+^, whereas the amount of co-immunoprecipitated CK has no difference with or without addition of Ca^2+^. By contrast, CEPT1 ablation completely abolishes the Co-IP (Figure 4E). These observations indicate that Ca^2+^-regulated CCTα recruitment to CEPT1 may play a key role in the assembly of the enzymatic complexes at ER membrane, and that the interaction between CEPT1 and CK is stable and not regulated by Ca^2+^. To rule out the possibility that the above results are caused by reduction in protein expression, immunoblot was conducted and showed that the expression levels of CK and CEPT1 are not significantly changed under TPP2 depletion, wheras the level of CCTα becomes even significantly higher than controls (Figure 4F). In addition, in order to reveal whether CCTα recruitment to ER membrane would in turn change cytosolic Ca^2+^ level or not, we prepared TPP2 knockout cells which transiently express CCTα tagged with ER targeting KDEL sequence and fused to EGFP. Then we analyzed the cytosolic Ca^2+^ levels after Cal-590 staining. Our results showed that ectopic expression of ER-targeted CCTα significantly increases cytosolic Ca^2+^ level (Figure 4G1). As controls, we also analyzed the cytosolic Ca^2+^ levels of TPP2 knockout cells which transiently express acetyl-conA carboxylase (ACC), the rate-limiting enzyme in the de novo fatty acid synthesis pathway, and SERCA1 for ER Ca^2+^ refilling, both are also fused to EGFP. As expected, ectopic expression of ACC increases cytosolic Ca^2+^ level, while ectopic expression of SERCA1 decreases cytosolic Ca^2+^ level (Figure 4G1). The expression of CCTα-KDEL, ACC and SERCA1 fused to EGFP were verified by flow cytometry assay (Figure 4G2). Thus, our data suggest that TPP2 depletion-induced cytosolic Ca^2+^ decrease can cause insufficient biosynthesis of PC due to inefficient assembly of the CK-CCTα-CEPT1 complexes on ER membrane, and the PC shortage can in turn exacerbate Ca^2+^ dyshomeostasis.

### TPP2 depletion induces lipid dyshomeostasis of female mouse brain, which is characterized by a significant decrease in a signature cluster of PC species with saturated fatty acid chains

**Figure 5:**
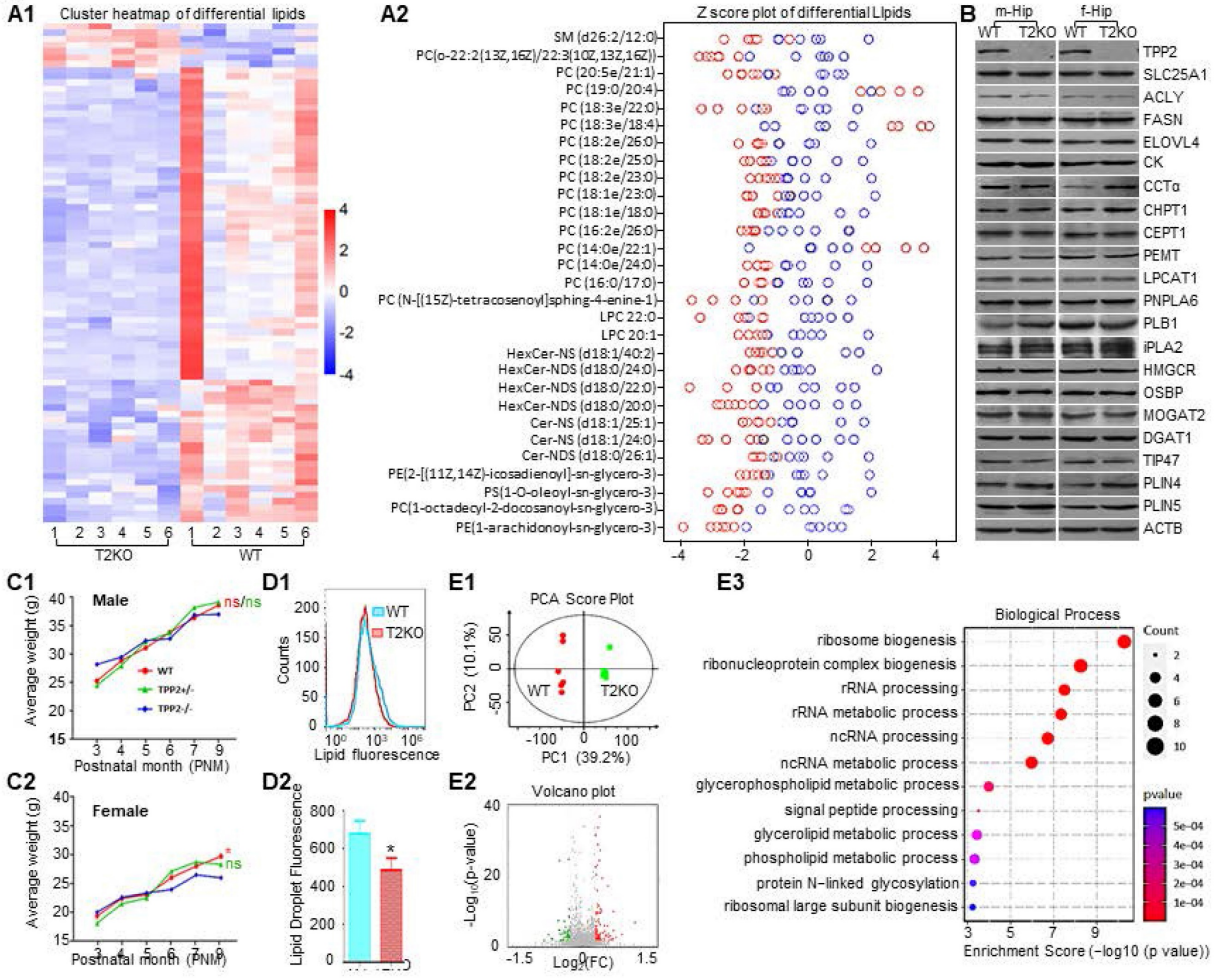
TPP2 depletion induces lipid dyshomeostasis in female mouse brain which is characterized with significant reduction of PC species with saturated fatty acid chains. (**A**) Lipidomics assay to identify lipidome difference between brains of WT and TPP2 knockout mouse at p180. (**A1**) Cluster heatmap showing the overall distribution and relative values of the differential lipids in WT and TPP2 knockout samples. The horizontal axis represents the experimental mice from which the brain samples were segregated. The vertical axis represents the differential lipids. Grid of colored squares depicts values of the differential lipids. The values are illustrated by a color, with larger values associated with darker colorings. The red and blue colors indicate levels of differential lipids with an increase and a decrease, respectively. (**A2**) The Z-score plot showing the level of lipid compounds on the same horizontal plane. The horizontal axis represents the Z-score values of differential lipids, and the vertical axis lists the top differential lipids which are sorted from top to bottom by p-value from smallest (0.001) to largest (0.05). Lipids with Z-score exceeding 4 or -4 are not displayed. Both the two-way clustering analysis and Z-score plot are based on the LC-MS/MS data which were obtained from 6 WT and TPP2 knockout mice of the same age, respectively. These data showed that the lipidome difference between WT and TPP2 knockout mouse brains is mainly PC. Except for the increase in the level of three species of PC with longer and unsaturated fatty acid chains, the level of all the other species of PC with saturated fatty acid chains decrease (Figure 5A). In addition, other types of differential lipids in the list also significantly decrease in TPP2 knockout samples. (**B**) Immunoblot analysis of the lipid metabolic enzymes and transporters which are closely related to PC homeostasis in WT and TPP2 knockout mouse brains. Except for CCTα and PLIN4, which clearly become more in TPP2 knockout female mouse brain sample probably due to functional compensation, there is no significant change for other proteins in both male and female samples under TPP2 depletion. SLC25A1: solute carrier family 25 member 1; ACLY: ATP-citrate lyase; FASN: fatty acid synthase; ELOVL4: elongation of very long chain fatty acids like 4; CK: choline kinase; CCTα: CTP:phosphocholine cytidylyltransferase alpha; CHPT1: choline phosphotransferase 1; CEPT1: choline-ethanolamine phosphotransferase 1; PEMT: phosphatidylethanolamine n-methyltransferase; LPCAT1: lysophosphatidylcholine acyltransferase 1; PNPLA6: patatin like phospholipase domain containing 6; PLB1: phospholipase B1; iPLA2: Ca^2+^-independent phospholipase A2; HMGCR: 3-hydroxy-3-methylglutaryl coenzyme-A; OSBP: oxysterol-binding protein; MOGAT2: monoacylglycerol o-acyltransferase 2; DGAT1: diacylglycerol acyltransferase 1; TIP47: tail interacting protein 47; PLIN4: perilipin 4; PLIN5: perilipin 5. (**C**) Statistical analysis of body weight in mice of different genotypes and age groups. Both male and female TPP2 knockout mice have slightly higher body weight than WT and heterozygous mice before the age of 5 months. However, after 5 months, the body weight of TPP2 knockout mice is lower than WT and heterozygous mice. In particular, the body weight of female TPP2 gene knockout mice was significantly lower than that of WT and heterozygous mice. The numbers of WT and T2KO groups for statistics are up to 24 for both male and female mice, while the numbers of heterozygous group numbers are up to 31 and 16 for male and female group, respectively. (**D**) LD quantitation of freshly prepared hippocampal neural cells. (**D1**) Flow cytometry histogram overlay of WT and TPP2 knockout neural cells after LD fluorescence staining. (**D2**) Statistical graph of the geometric means of fluorescence intensity in WT and TPP2 knockout samples showing that TPP2 depletion significantly decreases LD staining. n = 6, * p ≤ 0.05. (**E**) iTRAQ protein quantification assay for identifying proteome difference between WT and TPP2 knockout 293T cells. (**E1**) PCA analysis showing the overall distribution trend between WT and TPP2 knockout samples. The horizontal axis PC1 and vertical axis PC2 represent the scores of the first and second ranked principal components, respectively. Scatters of different colors represent samples from different experimental groups, and ellipse represents a 95% confidence interval. (**E2**) Volcano plot displaying the overall distribution of differential proteins. The horizontal axis represents the fold changes of protein in different groups (log2 (Fold Change)), and the vertical axis represents the significance level of differences (- log10 (p-value)). Each point in the volcano plot represents a protein, with significantly upregulated proteins represented by red dots, significantly downregulated proteins represented by green dots. (**E3**) Dot plot displaying the top 12 enriched GO biological processes (BPs) of downregulated proteins (Figure S1B). The horizontal axis represents the enrichment score values of differential BPs, and the vertical axis lists the top differential BPs which are sorted from top to bottom by p-value from smallest (0.001) to largest (0.05). Circle size shows the modulated genes per BP.

Given that TPP2 depletion causes dimorphism in Ca^2+^ dyshomeostasis in hippocampal neurons of male and female mice and that TPP2 depletion-induced cytosolic Ca^2+^ reduction may cause insufficient PC biosynthesis, we performed lipidomics assay to determine the lipidome difference in the brains of 6 month-old WT and TPP2 knockout female mice. As expected, the two-way clustering analysis combined with Z-score calculation of obtained LC-MS/MS data revealed that the levels of 13 PC species with saturated fatty acid chains decrease under TPP2 depletion (Figure 5A). However, the levels of 3 PC species with longer and unsaturated fatty acid chains increase under TPP2 depletion (Figure 5A). Considering that TGN-localized CPT1 is more inclined to use PUFA to synthesize PC than ER-localized CEPT1 as abovementioned, we subsequently investigated whether the increased levels of PCs with longer and unsaturated fatty acid chains are attributed to more recruitment of CCTα to CPT1 in contrast to less recruitment of CCTα to CEPT1 under TPP2 depletion. Indeed, our PLA results displayed more interaction between CCTα and CPT1 in TPP2 knockout 293T cells (Figure S1B). This observation is in agreement with the canonical notion that the lipid compositions of TGN and ER membrane are different and the biophysical properties of the membrane surfaces contribute to the membrane binding of CCTα 19, 71-73. Of note, besides most PCs, the levels of all the other types of lipids displayed in the Z-score plot of differential lipids also significantly decrease in TPP2 knockout samples (Figure 5A). Among them, lysophosphatidylcholine (LPC), PE, and PC can transform to each other by the Lands' cycles 20, 74, while the sphigolipids including sphingomyelin (SM), ceramide, and ceramide derivatives interrelate with PC too 75. To rule out the possibility that dysregulation of lipid metabolic enzymes and transporters may contribute to PC lessening in female brains, immunoblotting analysis was performed. Interestingly, among the examined proteins in all samples, only the expression level of CCTα in the brains of female TPP2 knockout mice is significantly changed, but being upregulated rather than downregulated, (Figure 5B), confirming that the PC lessening in female brains is not caused by downregulation of CK, CCTα, CEPT1 and other investigated facors.

Furthermore, we also examined the body weights of WT and TPP2 knockout mice. Although both male and female TPP2 knockout mice have lower body weight than WT and heterozygous counterpart mice after 5 months of the age, only the weight loss of female TPP2 knockout mice is statistically significant as compared with WT and heterozygous mice (Figure 5C). Additionally, in consistence with inefficient assembly of the CK-CCTα-CEPT1 complexes on ER membrane and their roles in LD biogenesis 17, 56, 57, TPP2 depletion causes significant decline in LD staining of freshly isolated hippocampal neurons of newborn female baby mice (Figure 5D), whereas WT and TPP2 knockout male mice don't present significant difference in the same staining (Data not shown). Taken together, TPP2 depletion leads to accelerated weight loss especially in female mice, which may be attributed to insufficient biosynthesis of many species PC with saturated fatty acid chains in the brains and other tissues. Given that dysregulation of Ca^2+^ and PC can lead to ER stress and UPR 21-23, 76, we conducted iTRAQ protein quantification assay to identify proteome difference between WT and TPP2 knockout 293T cells. We found that the levels of 114 proteins increase while the levels of other 61 proteins decrease in TPP2 knockout cells as compared with WT controls (Figure 5E1-E2). The following GO enrichment analysis on the biological processes (BPs) of these dysregulated proteins revealed that the top 10 enriched BPs of the upregulated proteins are related to glycolysis and NADH/NAD metabolism (Figure S1C), while the top 10 enriched BPs of the downregulated proteins are related to lipid metabolism and ribosome biogenesis (Figure 5E3). Remarkably, glycolysis, energy production, lipid metabolism, and ribosome biogenesis are closely associated with ER stress 20, 77-80.

### TPP2 depletion causes ER stress in both 293T cells and primary hippocampal neurons of female mice, which can be reversed by adding TG, PC or ectopic expression of CCTα-KDEL

**Figure 6:**
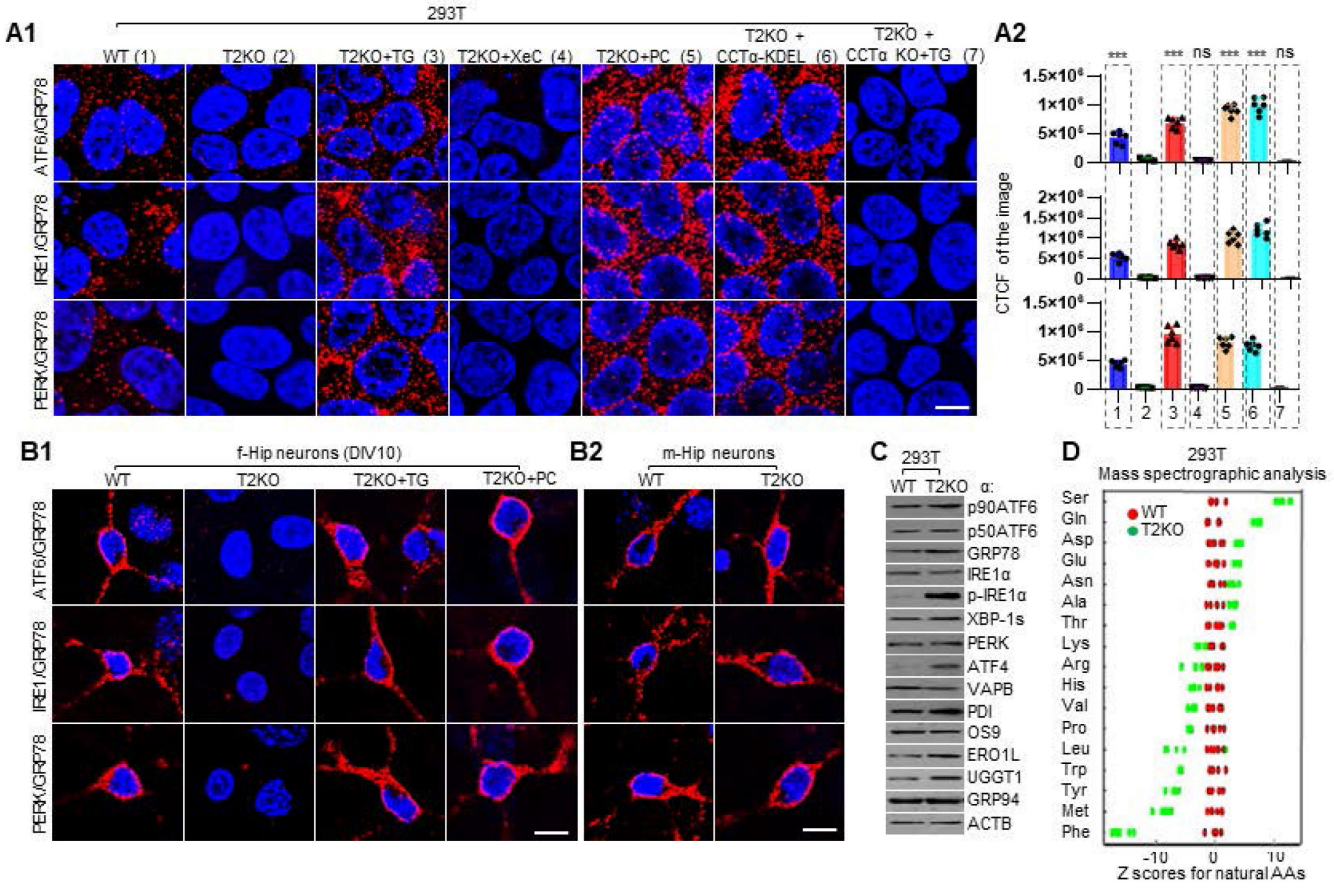
TPP2 depletion causes ER stress in both 293T cells and primary hippocampal neurons of female mice, which can be compromised by adding TG, PC or ectopic expression of CCTα-KDEL. (**A**) PLA for analysis of the interaction between GRP78 and ATF6, IRE1 and PERK in 293T cells with different genetic background and treatment under different conditions. (**A1**) TPP2 depletion completely eliminates the interaction between GRP78 and ATF6, IRE1, and PERK, which is reversed or even enhanced by transient treatment with TG, PC or ectopic expression of CCTα-KDEL, instead of XeC. In particular, when TPP2 and CCTα are doubly deleted, transient treatment with TG fails to restore the interaction between GRP78 and ATF6, IRE1 or PERK. For each pair of PPI, one representative scope from more than 20 confocal microscopic images is shown. Scale bars, 10 μm. (**A2**) Statistical graph of the CTCF values of the selected scopes of PPI pairs from independent experiments. n = 6, *** p ≤ 0.001, ^ns^ p > 0.05. 1: WT, 2: T2KO, 3: T2KO + TG, 4: T2KO + XeC, 5: T2KO + PC, 6: T2KO + CCTα-KDEL, 7: T2KO/CCTα DKO + TG. TG treatment: 5 μM for 5 h at 37°C, PC treatment: 5 μM for 24 h at 37°C. (**B**) PLA for analysis of the interaction between GRP78 and ATF6, IRE1 and PERK in DIV10 mouse primary hippocampal neurons. (**B1**) TPP2 depletion completely eliminates the interaction between GRP78 and ATF6, IRE1, and PERK in female mouse hippocampal neurons, which is reversed or even enhanced by transient treatment with TG or PC. For each pair of PPI, one representative scope from more than 20 confocal microscopic images is shown. Scale bars, 10 μm. (**B2**) TPP2 depletion hardly affects the interaction between GRP78 and ATF6, IRE1, and PERK in male mouse hippocampal neurons. For each pair of PPI, one representative scope from more than 20 confocal microscopic images is shown. Scale bars, 10 μm. (**C**) Immunoblot for analysis of the expression levels of ER stress-related proteins in WT and TPP2 knockout 293T cells. The three classic branches of ER stress markers including p90ATF6/p50ATF6, IRE1α/p-IRE1α/XBP-1s, and PERK/ATF4 together with indicators of UPR activation which is closely related to ER stress were tested. The data clearly showed that TPP2 depletion results in ER stress characterized by cleavage of p90ATF6, autophosphorylation of IRE1, ATF4 overexpression, and upregulation of GRP78, PDI, ERO1L, and UGGT1. (**D**) Mass spectromic analysis of the levels of free amino acids in WT and TPP2 knockout 293T cells. This data clearly showed that TPP2 knockout cells are highly enriched with ATF4-dependent UPR amino acid signatures which are preferentially enriched in secreted and extracellular matrix proteins during ER stress. Despite that, UPR usually leads to inhibition of ER targeting proteins.

ER is a membranous and dynamic organelle that participates in and regulates multiple biological processes, including Ca^2+^ storage, lipid synthesis, protein synthesis, modification and folding 79, 81, 82. As such, ER homeostasis is crucial for maintenance of the fine balance between health and disease, and different exogenous or endogenous factors which cause disturbance of ER homeostasis can lead to ER stress 82, 83. There are three highly conserved ER stress sensors, namely ATF6, IRE1, and PERK, which can probe various stressors and be activated through different mechanisms to regain ER homeostasis 21-23, 76. For example, all the three stress sensors are activated by accumulation of unfolded proteins within ER and by LBS 23. Importantly, there exists a glucose-regulated protein 78 (GRP78/BiP/HSPA5) binding-release mechanism, which is involved in the activation of all three branches of UPR 84, 85. Under non-stressed conditions, GRP78 binds to and inactivates all three ER stress transducers, while under ER stress GRP78 is released from them, leading to the activation of the UPR pathways 86. As previously described, dysregulation of Ca^2+^ and PC can lead to ER stress and UPR 21-23, 76, and TPP2 depletion causes dyshomeostasis of both Ca^2+^ and PC, therefore, we first tested the activation status of these three ER sensors by detecting their interactions with GRP78 in TPP2 knockout 293T model cells and their control cells. We found that TPP2 depletion leads to complete dissociation of GRP78 from ATF6, IRE1, or PERK, whereas in WT cells their interactions are clearly presented (Figure 6A). Importantly, this phenomenon can be restored or even excessively reversed by transient treatment with TG, PC or transient expression of CCTα-KDEL, but not XeC, which is an IP3R antagonist (Figure 6A). In particular, when TPP2 and CCTα are doubly deleted, transient treatment with TG fails to restore the interaction between GRP78 and ATF6, IRE1 or PERK (Figure 6A), suggesting that transient treatment with TG reverses the interaction between GRP78 and ER stress sensors by promoting PC synthesis. Subsequently, we carried out the same experiment on TPP2 knockout hippocampal neurons of female mice and their WT controls. We found that TPP2 depletion completely eliminates the interaction between GRP78 and ATF6, IRE1, or PERK in hippocampal neurons, whereas in WT neurons their interactions are clearly presented (Figure 6B1). Similarly, this phenomenon can be restored by transient treatment with TG or PC (Figure 6B1). By contrast, TPP2 depletion has nearly no effect on the interaction between GRP78 and ATF6, IRE1, or PERK in hippocampal neurons of male mice (Figure 6B2).

ER stress-activated three branches of signaling pathways regulate transcription, translation, or posttranslational modification of the three ER stress sensors themselves and hundreds of other proteins to alleviate ER stress and restore ER homeostasis 87, 88. Therefore, we examined the levels of p90ATF6/p50ATF6 89, IRE1α/p-IRE1α/XBP-1s 90, 91, PERK/ATF4 92, and some other indicators of ER stress 77, 93, 94 in WT and TPP2 knockout cells. The data clearly showed that TPP2 depletion elicits ER stress and results in activation of all the three branches of signaling pathway, which is characterized by cleavage of p90ATF6, autophosphorylation of IRE1, overexpression of ATF4, and upregulation of GRP78, protein disulfide isomerase (PDI), ER oxidoreductin-1-like (ERO1L), and UDP-glucose glycoprotein glucosyltransferase 1 (UGGT1) (Figure 6C). Remarkably, ATF4 is one of the secondary effectors of PERK and its translation is selectively induced by phosphorylation of eukaryotic translation initiation 2α (eIF2α), which routinely inhibits global translation initiation during ER stress 88, 95. In particular, ATF4 is known to induce the amino acid biosynthesis coupled to protein expression demand during ER stress 96-99. It was reported that biosynthesis of a specific subset of amino acids, including serine (Ser), cysteine (Cys), glycine (Gly), alanine (Ala), asparagine (Asn), aspartic acid (Asp), glutamic acid (Glu) and proline (Pro), is induced together with upregulation of their corresponding tRNA synthetases in the late ER stress response 99, 100. We performed mass spectromic analysis of the levels of amino acids in WT and TPP2 knockout 293T cells. Our data clearly showed that TPP2 knockout cells are highly enriched with ATF4-dependent UPR amino acid signatures such as Ser, Gln, Asp, Glu, Asn and Ala (Figure 6D), which are preferentially enriched in secreted and extracellular matrix proteins during ER stress, despite that UPR usually leads to repression of ER target proteins 99, 101.

### TPP2 depletion causes ER stress-associated neuronal apoptosis and autophagy in adult female mice, which are correlated with estrogen diminution in the brains of these mice

**Figure 7:**
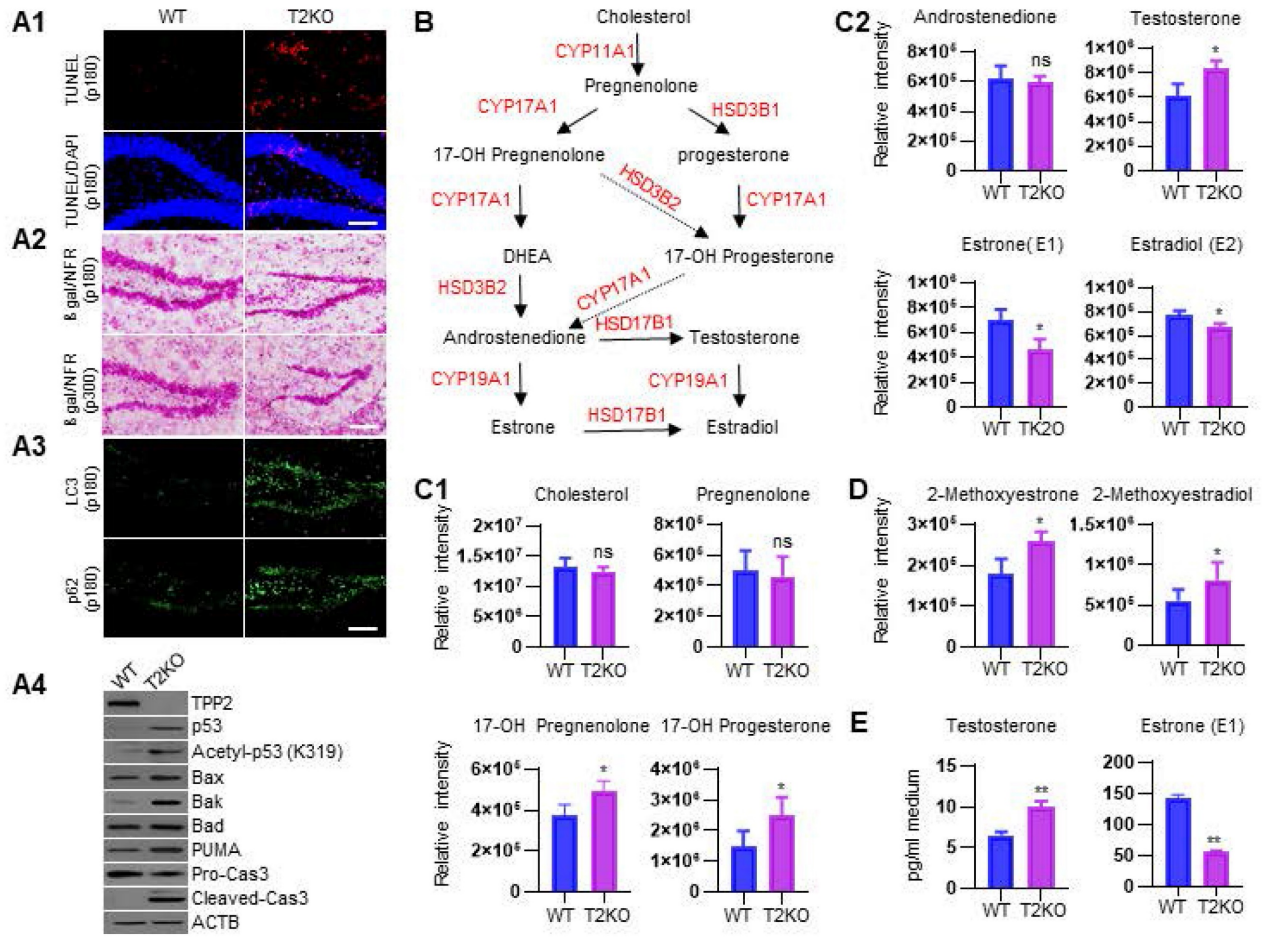
TPP2 depletion causes apoptosis and autophagy of hippocampal neurons, which are correlated with testosterone accumulation and estrogen diminution in the brains of female mice. (**A**) TPP2 depletion causes neuronal apoptosis and autophagy in hippocampi of female mice. (**A1**) TUNEL staining showed a significant increase of apoptosis in GCL of hippocampal DG region of 6 month-old TPP2 knockout female mice as compared with control animals. (**A2**) β-galactosidase staining combined with NFR staining showed no premature senescence in GCL of hippocampal DG region of both 6 month-old and 10 month-old TPP2 knockout female mice as compared with control animals. (**A3**) IHC staining of two autophagy markers showed a significant increase of autophagic flux in hippocampi of 6 month-old TPP2 knockout female mice as compared with control animals. (**A4**) Immunoblot showed activation of the intrinsic apoptotic pathway in TPP2 knockout cells. (**B**) Outline of the neurosteroidogenesis pathway showing estrogen biosynthesis in brain through metabolism of precursors. Estrogen and its precursors are shown in Black and the neurosteroidogenic enzymes are shown in Red. (**C**) Mass spectromic analysis of the levels of estrogen and its precursors in the brains of WT and TPP2 knockout female mice. This data showed that estrogen precursors including 17-OH Pregnenolone, 17-OH Progesterone (**C1**), and testosterone (**C2**) accumulate while estrogen (**C2**) decreases under TPP2 depletion. (**D**) Mass spectromic analysis of the levels of the endogenous metabolites of estrogen in the brains of WT and TPP2 knockout female mice. This data showed that both estrone metabolite (2-Methoxyestrone) and estradiol metabolite (2-Methoxyestradiol) are significantly increased under TPP2 depletion. (**E**) Immunoassay for analysis of testosterone and estrogen in the culture medium of DIV 10 hippocampal neurons of female mice. In consistence with the MS data shown in (C), the content of testosterone increases while estrogen decreases in the culture medium of TPP2 knockout hippocampal neurons.

As mentioned above, ER stress can activate three branches of signaling pathways to alleviate ER stress and restore ER homeostasis. However, under high-level or chronic ER stress, these adaptive changes may eventually be overshadowed by a terminal UPR program that commit cells to degeneration or death, thus being implicated in the pathogenesis of a growing list of human diseases 102, 103. Given that TPP2 depletion causes Ca^2+^ dyshomeostasis, PC ratio imbalance, and thereforth chronic ER stress, we tested whether these would lead to neuronal death or premature senescence like other cells 3, 5. We performed TUNEL assay and β-galactosidase staining on hippocampal slices and found that 6 month-old TPP2 knockout female mice showed significantly increased apoptotic staining in granule cell layer (GCL) of hippocampal DG region as compared to control animals (Figure 7A1), whereas neither 6 month-old nor 10 month-old TPP2 knockout female mice showed clear premature senescent staining in the same region as compared to control animals (Figure 7A2). We also conducted IHC staining of two autophagy markers on hippocampal slices and immunoblot of some members on the intrinsic apoptotic pathway. Interestingly, both microtubule-associated protein 1A/1B-light chain 3 (LC3) and p62/SQSTM1 are increased in hippocampi of TPP2 knockout female mice (Figure 7A3), while p53, acetylated p53, Bcl-2 associated X protein (Bax), BCL2 antagonist/killer 1 (Bak), and p53 upregulated modulator of apoptosis (PUMA) are increased too, together with cleaved caspase 3 under TPP2 depletion (Figure 7A4). Clearly, TPP2 depletion causes neuronal apoptosis and autophagy simultaneously. However, the causal relationship between apoptosis and autophagy in this case is unclear. It has been reported that apoptosis induces autophagy, and autophagy in turn blocks apoptosis, they two together determine the fates of cells 104, 105. To identify the detailed mechanism of TPP2 depletion-induced dimorphism on cell death and autophagy, we then conducted LC-MS/MS-based metabolomics analysis of sex hormones in the brains of TPP2 knockout female mice and WT control animals. Sexual hormones, especially estrogen, have been shown to have very important protective effects on the nervous system 106-108. Our data showed that estrogen precursors including 17-OH Pregnenolone, 17-OH Progesterone, and testosterone increase, whereas estrogen decreases in the brain of 6 month-old TPP2 knockout female mice as compared to WT controls (Figure 7C1-C2). In addition, both estrone metabolite (2-Methoxyestrone) and estradiol metabolite (2-Methoxyestradiol) are significantly increased under TPP2 depletion (Figure 7D). In consistence with this MS data, immunoassay analysis of sex hormones in the culture medium of female TPP2 knockout mouse hippocampal neurons showed that the content of testosterone increases while estrogen decreases as compared to controls (Figure 7E).

### Time-dependent diminution of aromatase in hippocampus of TPP2 knockout female mouse is faster than in WT hippocampus, and intravenous injection of AAV expressing aromatase significantly rescue the learning and memory impairment of female knockout mice

**Figure 8:**
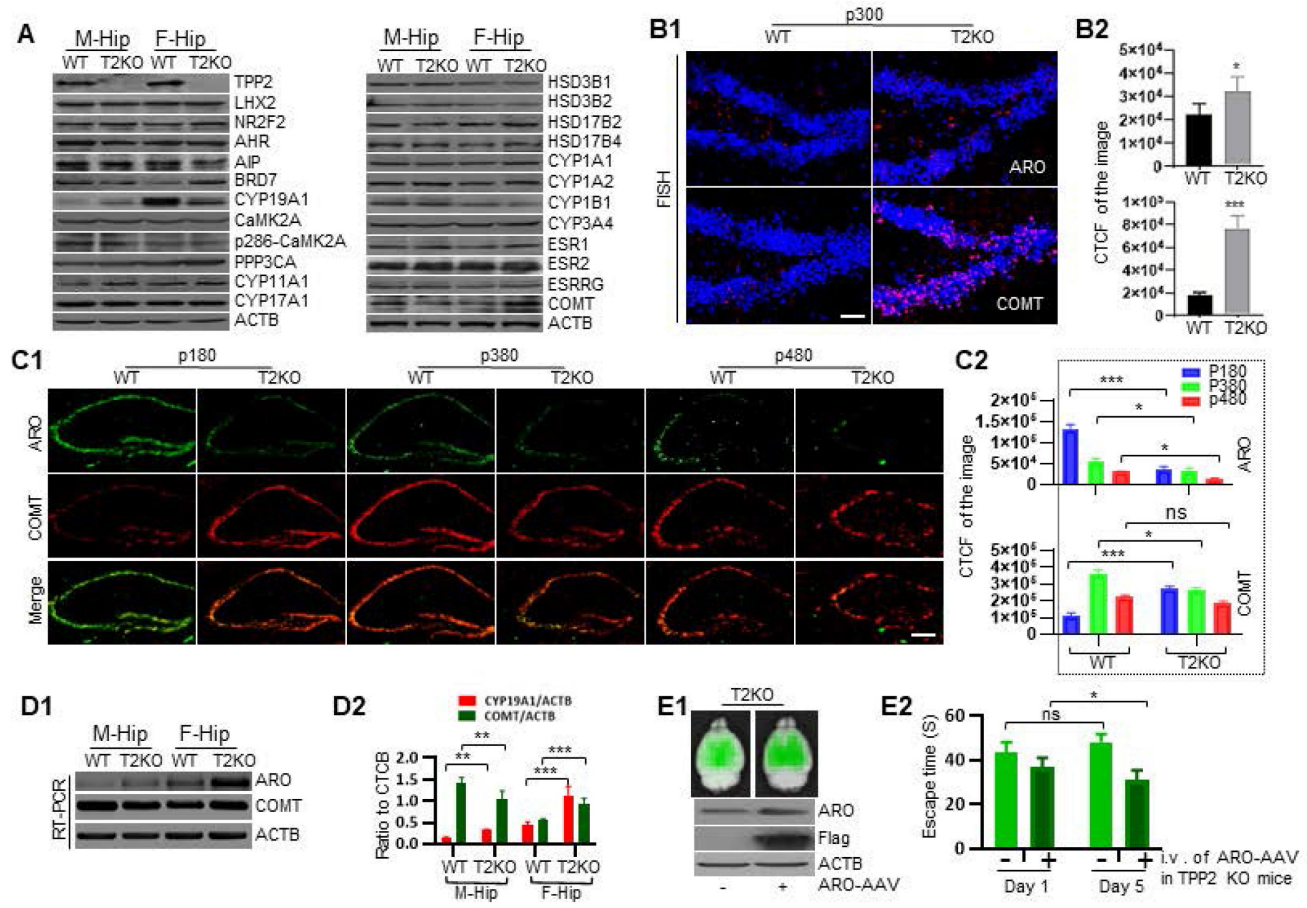
Time-dependent diminution of aromatase in hippocampus of TPP2 knockout female mouse is faster than in WT hippocampus, and intravenous injection of AAV expressing aromatase significantly rescue the learning and memory of female knockout mice. (**A**) Immunoblotting analysis of aromatase, proteins involved in aromatase and estrogen metabolism, and estrogen receptors in WT and TPP2 knockout mouse hippocampi at p180. The data showed that the aromatase in the hippocampus of TPP2 knockout female mouse is significantly lower than that in the WT controls, whereas it is exactly the opposite for males, though the overall level in males is significantly lower than those in females. By comparison, the levels of COMT in the hippocampus of TPP2 knockout female mouse is significantly higher than that in WT control, while in males it is exactly the opposite. Besides, the levels of BRD7 and PPP3CA (calcineurin) in the hippocampus of TPP2 knockout female mouse are significantly higher than that in WT controls. No significant changes were found in other proteins. (**B**) Fluorescence in Situ Hybridization (FISH) for analysis of mRNAs of aromatase and COMT in hippocampi of WT and TPP2 knockout female mice at p180. (**B1**) FISH images showed that the mRNA levels of aromatase and COMT in the hippocampus of TPP2 knockout female mouse are higher than in WT controls. (**B2**) Statistical graph of the CTCF values of the selected scopes from 6 independent experiments. Brain slice from the same brain area of 6 different mice at the same age were used for staining in each case. Scale bars, 50 μm. n =6, *** p ≤ 0.001, * p ≤ 0.05. (**C**) IHC analysis of aromatase and COMT in hippocampi of WT and TPP2 knockout female mice in different age periods. (**C1**) At p180, p380, and p480, aromatase is significantly reduced in hippocampus of TPP2 knockout female mouse as compared with WT control. By contrast, COMT is significantly increased at p180 and decreased at p380 and p480 in the hippocampi of TPP2 knockout female mouse as compared with WT ones. One representative holographic scanning of the hippocampus is shown. Brain slice from the same brain area of 6 different mice at the same age were used for staining in each case. Scale bars, 300 μm. (**C2**) Statistical graph of the CTCF values of the selected scopes from 6 independent experiments. n = 6, *** p ≤ 0.001, * p ≤ 0.05, **^ns^** p > 0.05. (**D**) Semi-quantitative RT-PCR to analyse the mRNAs of aromatase and COMT in hippocampi of WT and TPP2 knockout mice at p180. (**D1**) Agarose gel images showing that the mRNA levels of aromatase in hippocampi of both male and female TPP2 knockout mice are higher than those in the WT counterparts, though the overall expression level in females is significantly higher than in males. By contrast, the mRNA level of COMT in the hippocampus of TPP2 knockout female mouse is higher as compared with WT control. However, in males, it is lower as compared with WT control. (**D2**) Statistical graph of the ratio of specific band to reference from independent experiments. n = 6, *** p ≤ 0.001, ** p ≤ 0.01. (**E**) Gene therapy for TPP2 depletion-induced learning and memory impairment using AAV vectors. (**E1**) Ex-vivo imaging and immunoblot to detect the expression of aromatase in the brains from mice injected with HBAAV2/BBB-hSyn-mCyp19a1-T2A-ZsGreen or HBAAV2/BBB-hSyn-ZsGreen virus particles. These data showed that the AAVs are capapable of widespread expression of aromatase and ZsGreen after intravenous delivery. (**E2**) MWM test to assess spatial learning and memory of TPP2 knockout female mice at p300 with or without intravenous delivery of AAV. This data showed that intravenous delivery of HBAAV2/BBB-hSyn-mCyp19a1-T2A-ZsGreen significantly rescue TPP2 depletion-caused learning and memory impairment. n = 6, * p ≤ 0.05, **^ns^** p > 0.05.

In the light of above data showing that estrogen decreases while its precursors and metabolites increase, we speculated that these abnormalities may be attributed to the expression level changes of enzymes which catalyze estrogen biosynthesis and decomposition. Thus, we first checked a series of enzymes involved in estrogen production, estrogen degradation, and estrogen receptors in 6 month-old mouse hippocampi through immunoblot. As expected, among the examined proteins, the level of aromatase in the hippocampus of TPP2 knockout female mouse is significantly lower than in WT control, whereas it is exactly the opposite for males, though the overall level in males is significantly lower than in females. By comparison, the level of Catechol-O-methyltransferase (COMT) in the hippocampus of TPP2 knockout female mouse is significantly higher than in WT control, while in males it is exactly the opposite (Figure 8A). Besides, the levels of bromodomain-containing protein 7 (BRD7) and protein phosphatase 3 catalytic subunit alpha (PPP3CA/calcineurin) in the hippocampus of TPP2 knockout female mouse are significantly higher than in WT controls. No significant changes were found in other proteins (Figure 8A). Of note, BRD7 is a co-factor of p53 and Breast cancer gene 1 (BRCA1), both of which suppress the transcription of aromatase 109-112, while PPP3CA modulates the activity and stability of aromatase 113-115. As such, we examined the mRNA levels of aromatase and COMT in 6 month-old mouse hippocampus using Fluorescence in situ hybridization (FISH) and semi-quantitative RT-PCR. Astonishingly, our data showed that the mRNA levels of aromatase and COMT in the hippocampus of TPP2 knockout female mouse is significantly higher than in WT control (Figure 8B and D). However, in the hippocampus of male mice, the mRNA level of aromatase increases while that of COMT decreases, though the overall level of aromatase mRNA in males is significantly lower than those in females (Figure 8D). These observations indicate that the transcription of aromatase gene in hippocampus of TPP2 knockout female mouse may be regulated by other factors instead of p53 and BRD7, both of which are increased under TPP2 depletion and they are expected to suppress the transcription of aromatase gene according to abovementioned references.

Given that the level of aromatase in the hippocampus of TPP2 knockout female mouse is significantly lower, whereas the mRNA level of aromatase is significantly higher, as compared with WT controls, it seems that aromatase may be more quickly degraded rather than less transcribed and translated under TPP2 depletion as compared with WT control. Thus, we compared aromatase and COMT in hippocampi of WT and TPP2 knockout female mice in different age periods by IHC staining. The data showed that, at postnatal day 180 (p180), p380, and p480, aromatase is significantly reduced in a time dependent manner in the hippocampi of TPP2 knockout female mouse as compared with WT controls (Figure 8C). By contrast, COMT is significantly increased at p180 and decreased at p380 and p480 in the hippocampi of TPP2 knockout female mouse as compared with WT animals (Figure 8C). As known, aromatase catalyzes androgen to estrogen and is widely expressed in the brain of all mammals 116, 117. Diverse cognitive functions including learning and memory are influenced by locally produced estrogen in brain 118-120. As such, we further investigated the learning and memory ability of TPP2 knockout female mice, which have lower levels of aromatase and estrogen in their hippocampi as compared with WT controls as described above. Intriguingly, our MWM test result showed that more than 6 months old TPP2 knockout female mice completely lose their spatial learning and memory abilities (Figure 8E2, Figure 10A). Furthermore, the learning and memory deficit of these mice can significantly recover one month later after intravenous delivery of AAV harbouring aromatase expressing cassette (Figure 8E1-E2).

### TPP2 depletion causes autophagic degradation of aromatase via ATF6-SYVN1-UCHL1 axis

**Figure 9:**
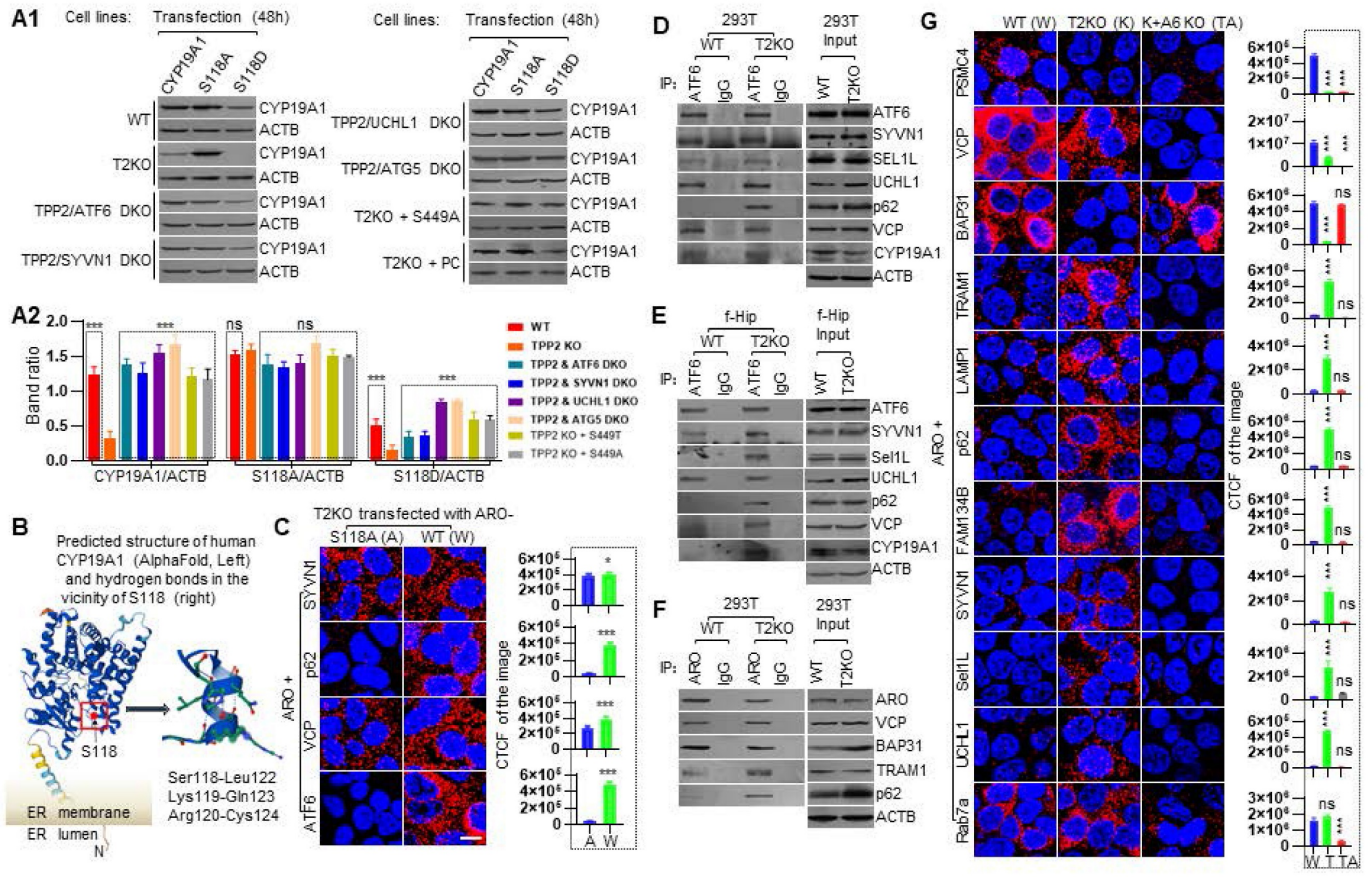
TPP2 depletion results in autophagic degradation of CYP19A1 via ATF6-SYVN1-UCHL1 axis, and under TPP2 depletion ATF6 redirects CYP19A1 from its interaction with BAP31 to TRAM1, thereby altering its degradation pathway from ubiquitin-proteasome system (UPS) to autophagy. (**A**) Immunoblotting analysis of the stability of WT, S118A, and S118D mutants of CYP19A1 ectopically expressed in WT 293T cells and 293T cells with different genetic modifications. (**A1**) One representative western blot result from 3 times repeated experiments is shown. (**A2**) Quantitative analysis of western blot results from 3 times repeated experiments showed that WT and S118D mutated CYP19A1 are significantly reduced in TPP2 knockout cells as compared with WT cells, TPP2/ATF6 double knockout (DKO) cells, TPP2/SYVN1 DKO cells, TPP2/UCHL1 DKO cells, TPP2/ATF5 DKO cells, and TPP2 KO cells ectopically expressing TPP2 S449A mutant or preincubated with PC. By comparison, the phosphomimetic S118D mutation of CYP19A1 decreases its stability, while the phospho-inhibiting S118A mutation of CYP19A1 increases its stability. Taken together, TPP2 depletion causes autophagic degradation of CYP19A1 in a manner dependent on ATF6, SYVN1, UCHL1, ATF5, and phosphorylation of S118. And this autophagic process can be prevented by TPP2 S448A and S449T mutants. (**B**) Predicted structure of human aromatase by AlphaFold and the hydrogen bonds in the vicinity of S118. (**Left**) The signal sequence and its following signal anchors (SAs) of aromatase mediates translocation of a short N-terminal hydrophillic domain to ER lumen and integrates the protein in the membrane, with the remainder of the protein residing in the cytosol. (**Right**) The amino acid context surrounding S118 is highly conserved among diverse animal species and its phosphorylation by an AGC-like kinase may form more stable hydrogen bonds with surrounding Arg (R) or Lys (K), which is related to the stability of aromatase. (**C**) PLA for analysis of the interaction between aromatase and SYVN1, p62, VCP, and ATF6 in TPP2 knockout 293T cells 24h after transfection with CYP19A1-FLAG or CYP19A1-S118A-FLAG expressing vector. (**C1**) TPP2 depletion significantly augments the interactions between WT CYP19A1 and SYVN1, p62, VCP, and ATF6 as compared with the interactions between CYP19A1-S118A and these proteins. For each pair of PPI, one representative scope from more than 20 confocal microscopic images is shown. Scale bars, 10 μm. (**C2**) Statistical graph of the CTCF values of the selected scopes of PPI pairs from independent experiments. n =6, *** p ≤ 0.001, ** p ≤ 0.01, * p ≤ 0.05. (**D**) Co-IP combined with immunoblot for analysis of the interaction between ATF6 and SYVN1, SEL1L, UCHL1, p62, VCP, and CYP19A1 in WT and TPP2 knockout 293T cells 48h after transfection with CYP19A1-FLAG expressing vector. (**Left**) TPP2 depletion causes Co-IP of p62 with ATF6, SEL1L, UCHL1, VCP and CYP19A1. By contrast, at presence of TPP2, p62 is missing in the co-immunoprecipitates. (**Right**) Western blot results of homogenates showed that there is no clear change in the expression level of these proteins except p62 and CYP19A1, which is significantly decreased under TPP2 depletion. By note, CYP19A1 is enriched in co-immunoprecipitates under TPP2 depletion, though it is significantly decreased in homogenate of TPP2 knockout cells. (**E**) Co-IP combined with immunoblot for analysis of the interaction between ATF6 and SYVN1, SEL1L, UCHL1, p62, VCP, and CYP19A1 in female mouse hippocampus. (**Left**) TPP2 depletion causes co-immunoprecipitation of p62 with ATF6, SEL1L, UCHL1, VCP, and aromatase. By contrast, at presence of TPP2, p62, SEL1L, and aromatase are missing in the co-immunoprecipitates. (**Right**) Western blot results of homogenates showed that there is no clear change in the expression level of these proteins except p62 and aromatase, which is significantly decreased under TPP2 depletion in female mouse hippocampus. By note, CYP19A1 is enriched in co-immunoprecipitates under TPP2 depletion, though it is significantly decreased in homogenate of TPP2 knockout hippocampal neurons. (**F**) Co-IP combined with immunoblot for analysis of the interactions between aromatase and VCP, BAP31, TRAM1, and p62 in 293T cells. (**Left**) TPP2 depletion causes more Co-IP of aromatase with TRAM and p62 and less Co-IP of BAP31 as compared with WT controls. (**Right**) Western blot results of homogenates showed that the expression levels of BAP31 and p62 significantly increased under TPP2 depletion. One of the representative results of three times repeated experiments is shown. (**G**) PLA to examine the interactions between aromatase and UPS-related or autophagy-related proteins in WT 293T cells and 293T cells with different genetic modifications. The degradation of aromatase in WT and TPP2 knockout cells occurs in different pathways. (**Left**) The interactions between aromatase and PSMC4, VCP, and BAP31 (closely related to the ERAD pathway) in TPP2 knockout 293T cells is very significantly reduced or eliminated as compared with WT cells. However, ATF6 depletion, in addition to TPP2 depletion, can reverse the interaction between aromatase and BAP31, but cannot reverse the interaction between aromatase and PSMC4 and VCP. On the contrary, ATF6 depletion, in addition to TPP2 depletion, eliminates the interaction between aromatase and VCP. The interaction between aromatase and LAMP1, p62, FAM134B, TRAM1, SYVN1, and UCHL1 (closely related to autophagy pathway) in TPP2 knockout 293T cells is very significantly increased as compared with WT cells, and further knockout of ATF6 under TPP2 depletion can eliminate all the interactions between aromatase and abovementioned proteins. For each pair of PPI, one representative scope from more than 30 confocal microscopic images is shown. Scale bars, 10 μm. (**Right**) Statistical graph of the CTCF values of the selected scopes of PPI pairs from independent experiments. n =6, *** p ≤ 0.001, ^ns^ p > 0.05.

Disruption of aromatase homeostasis causes a multiplicity of ailments 121. However, although there is no objection that phosphorylation/dephosphorylation at S118 regulates both catalytic activity and stability of aromatase, it is still unclear how the enzyme is degraded, and available studies have even been conflicting 114, 115, 122. We found that aromatase and its phosphomimetic S118D mutated version are significantly reduced in TPP2 knockout 293T cells as compared with WT cells (Figure 9A). Given that TPP2 depletion causes ER stress, and the latter is closely associated with ERAD 123, 124, and that aromatase is located in ER membrane 125, we prepared TPP2 knockout 293T cell lines with additional depletion of ER stress sensors to disclose which branch of them is involved in aromatase degradation. It turned out that, additional ATF6 depletion significantly prevents degradation of aromatase and its phosphomimetic S118D mutated version (Figure 9A), whereas additional IRE1 or PERK depletion doesn't (Data not shown). Furthermore, we revealed that additional depletion of synoviolin (SYVN1/Hrd1), ubiquitin carboxy-terminal hydrolase l1 (UCHL1/PGP 9.5), and autophagy-related gene 5 (ATG5), and ectopic expression of TPP2 S449A/T mutants (only data from S449A is shown) or preincubation with PC significantly prevent degradation of aromatase and its S118D mutated version (Figure 9A). By comparison, the phospho-inhibiting S118A mutant of aromatase remain stable in all the cases (Figure 9A). Taken together, TPP2 depletion causes autophagic degradation of aromatase and its S118D mutated version in a manner dependent on ATF6, SYVN1, UCHL1, ATG5, and phosphorylation of S118. And this autophagic process can be prevented by ectopic expression of TPP2 non-enzymatic mutants and preincubation with PC.

In order to elucidate why the phospho-inhibiting S118A mutation of aromatase make the enzyme more stable, the predicted structure of human aromatase and the hydrogen bonds in the vicinity of S118 are displayed (Figure 9B). Aromatase is a type I transmembrane protein, with a short N-terminal hydrophillic domain in ER lumen, a transmembrane domain integrated in ER membrane, and a long C-terminal cytoplasmic domain (Figure 9B). Remarkably, the amino acid context surrounding S118 is highly conserved among diverse animal species and S118 can be phosphorylated by an AGC-like kinase 114. We assume that the S118 phosphorylation may form more stable hydrogen bonds with surrounding Arg (R) or Lys (K) and thereby make aromatase more unstable (Figure 9B). Consistent with this assumption, our PLA result showed that TPP2 depletion significantly augments the interactions between aromatase and SYVN1, p62, valosin-containing protein (VCP/p97), and ATF6 as compared with the interactions between aromatase-S118A and these proteins (Figure 9C). In particular, the interaction between aromatase and p62 or ATF6 under TPP2 depletion is almost completely disrupted by phospho-inhibiting S118A mutation (Figure 9C). As known, ATF6 is an ER stress sensor, while p62 is a selective cargo receptor and signaling adaptor, which serves as a platform for autophagosome formation 126, 127. Thus, we believe that ER stress-induced phosphorylation at S118 of aromatase is critical for its uptake by p62 in autophagy, and the phospho-inhibiting S118A mutation makes the enzyme more stable by blocking its conjugation with p62. In agreement with this notion, TPP2 depletion in either 293T cells or hippocampal neurons causes Co-IP of p62 with aromatase, ATF6, suppressor/enhancer of Lin-12-like (SEL1L), UCHL1, VCP as compared with WT controls (Figure 9D-E). Considering the critical role of ATF6 in aromatase degradation under TPP2 depletion, we further exploited the established ATF6 and TPP2 double knockout cell lines to investigate its detailed role in aromatase degradation by PLA. Our data showed that the interactions between aromatase and proteasome 26S subunit, ATPase 4 (PSMC4), VCP, and B-cell receptor-associated protein 31 (BAP31/BCAP31) are very significantly reduced or eliminated in TPP2 knockout 293T cells as compared with WT cells, whereas additional ATF6 depletion can reverse the interaction between aromatase and BAP31, but not the interaction between aromatase and PSMC4 and VCP (Figure 9G). Interestingly, additional ATF6 depletion even completely eliminates the interaction between aromatase and VCP (Figure 9G). Moreover, the interactions between aromatase and lysosomal associated membrane protein 1 (LAMP1), p62, family with sequence similarity 134, member B (FAM134B/JK-1/RETREG1), translocating chain-associated membrane protein 1 (TRAM1), SYVN1, and UCHL1 in TPP2 knockout 293T cells are very significantly increased as compared with WT cells, and further knockout of ATF6 under TPP2 depletion can eliminate all the interactions between aromatase and abovementioned proteins (Figure 9G). Taken together, TPP2 depletion causes autophagic degradation of aromatase via ATF6-SYVN1-UCHL1 axis. Notably, although both BAP31 and TRAM1 are involved in degradation of ER transmembrane proteins, BAP31 is assumed to export these proteins for ERAD 128, 129. Given that ATF6 redirects aromatase from its interaction with BAP31 to TRAM1 under TPP2 depletion, we speculate that TRAM1 plays a splitter role in TPP2 depletion-caused autophagic degradation of aromatase via ATF6-SYVN1-UCHL1 axis.

### Ubiquitous and excitatory neuron-specific depletion of TPP2 impair learning and memory, attenuate LTP, and lead to significant decrease in local field potentials (LFPs) and Gamma-Theta rhythms and their coupling in hippocampal CA1 region of female mice

**Figure 10:**
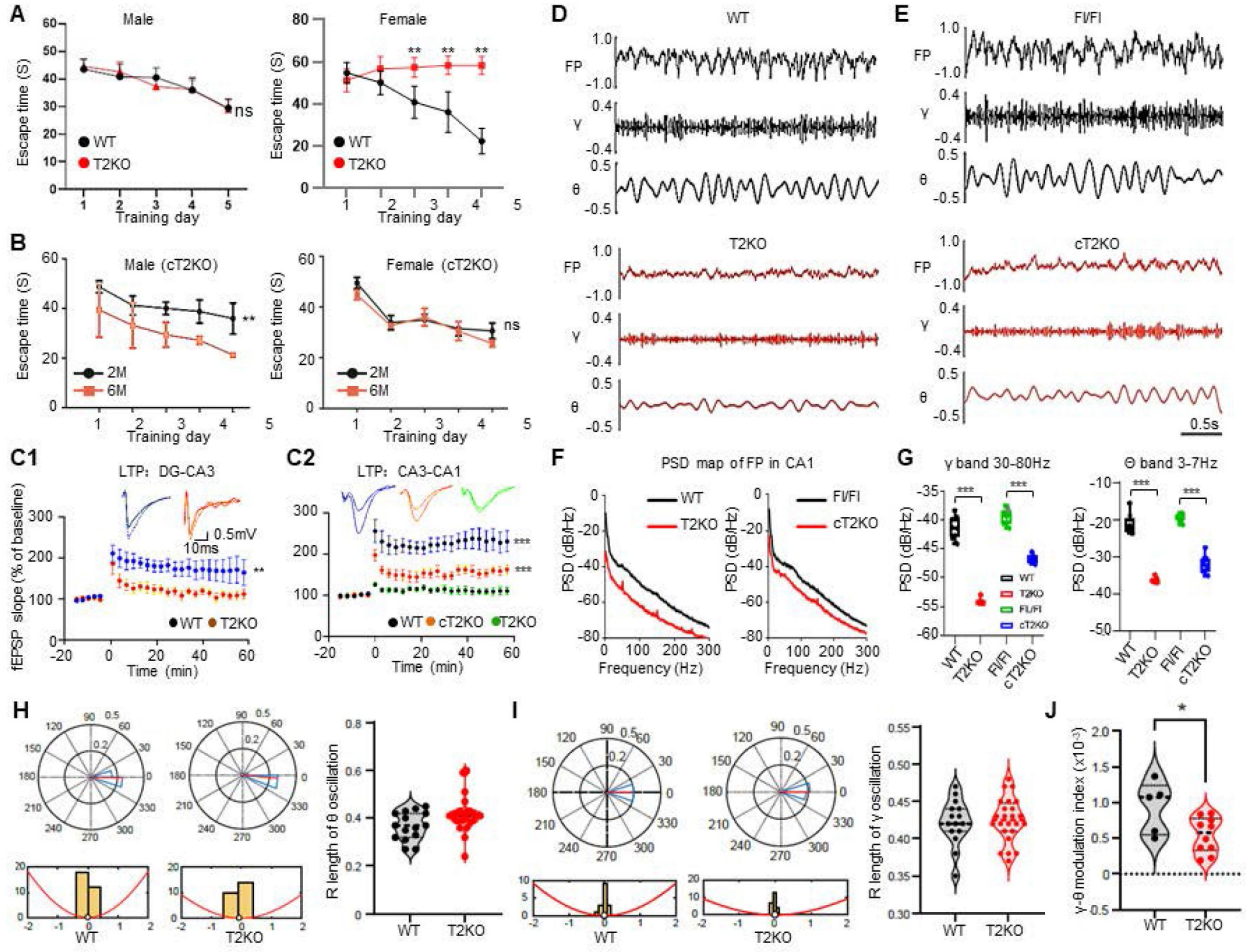
Both ubiquitous and neuron-specific depletion of TPP2 impair learning and memory, attenuate LTP, and cause significant decrease in LFPs, gamma-theta rhythms, and gamma-theta coupling in hippocampal CA1 region of 6 month-old female mice. (**A**) MWM test for assessing the learning ability and memory retention of 6 months old WT and TPP2 knockout mice. The mean escape latency is given for different trial days, data are represented as the mean ± SEM. (**Left**) Escape latency of male mice over 5 days was significantly decreased in both WT and TPP2 knockout groups. **^ns^** p > 0.05; WT, n = 14; TPP2 KO, n = 16. (**Right**) Escape latency of female mice over 5 days becomes gradually shorter only in the WT group, it even gradually becomes a little bit longer in TPP2 knockout group. ** p ≤ 0.01; WT, n = 21; TPP2 KO, n = 21. (**B**) MWM test for assessing the learning and memory functions of 2 and 6 months old neuron-specific TPP2 knockout mice. (**left**) Comparison of escape latency over 5 days of 2 and 6 months old male TPP2 knockout mice. The data showing that the escape latency of 6 months old group (6M) is significantly decreased as compared with 2 months old group (2M). ** p ≤ 0.01; 2M, n = 24; 6M, n = 11. (**Right**) Comparison of escape latency of 2 and 6 months old female TPP2 knockout mice. The data showing that there is no statistical significant difference between 2M and 6M group over the 5 days period. **^ns^** p > 0.05; ** p ≤ 0.01; 2M, n = 28; 6M, n = 15. (**C**) Brain slice physiology for analysis of LTP in the DG-CA3 or CA3-CA1 connection of female WT, neuron-specific TPP2 knockout, and ubiquitous TPP2 knockout mice (p180). (**C1**) Diagram of DG-CA3 LTP showing the normalized slopes of field excitatory postsynaptic potentials (fEPSP) in WT (blue) and TPP2 knockout (orange) mice before, during and after high frequency stimulation (HFS). Stimulation was delivered every 20 s. The short-term potentiation (STP, 1-2 min) and LTP (50-60 min) of the fEPSP was detected. The mean slope of the fEPSPs before HFS is defined as 100%. The insets give the examples of fEPSP before induction (solid trace) and 30 min after HFS (dashed trace). For this experiment, 16 slices from 8 WT and TPP2 knockout mice were tested, respectively. This data showed that the LTP is significantly attenuated under ubiquitous TPP2 depletion. (**C2**) Diagram of CA3-CA1 LTP illustrating the normalized slopes of fEPSP in WT (blue), neuron-specific TPP2 knockout (orange), and ubiquitous TPP2 knockout (green) mice before and after HFS. The insets give the examples of fEPSP before induction (solid trace) and 30 min after HFS (dashed trace). For this experiment, 16 slices from 6 WT mice, 17 slices from 6 neuron conditional TPP2 knockout mice, and 12 slices from 3 ubiquitous TPP2 knockout mice were tested. This data showed that the LTP is also significantly attenuated under neuron-specific TPP2 depletion, although the degree of attenuation is not as large as that of ubiquitous TPP2 knockout. (**D**) & (**E**) In-vivo electrophysiology recording with microwire array electrode combined with OmniPlex® neural recording data acquisition system for analysis of LFPs (≤ 300Hz) in hippocampal CA1 region at p180 showing that LFPs and gamma-theta rhythms in hippocampal CA1 region of (**D**) WT/T2KO mice (WT: Black; T2KO: Red) and (**E**) Floxed (Fl/Fl)/Neuron-specific T2KO mice (Fl/Fl: Black; Neuron-specific T2KO: Red). These data showed that both ubiquitous and neuron conditional TPP2 depletion cause markedly reduced amplitude of LFPs and gamma-theta rhythms as compared with that of WT mice, although the reduction under neuron-specific TPP2 depletion is not as much as that in ubiquitous TPP2 knockout mice. For each case, one representative recording of 8 independent experiments conducted on mice of the same age from different litters is shown. (**F**) The typical LFP PSD in hippocampal CA1 region of (**left**) WT/T2KO mice (WT: Black; T2KO: Red) or (**Right**) Floxed/Neuron-specific TPP2 knockout mice (Fl/Fl: Black; Neuron-specific TPP2 knockout: Red). These data showed that the amplitude of the PSD in both ubiquitous and neuron-specific TPP2 knockout mice becomes markedly decreased, although the reduction under neuron-specific TPP2 depletion is not as much as that in ubiquitous TPP2 knockout mice. (**G**) Statistical graph of gamma (30-80 Hz, left) and theta (3-7 Hz, right) rhythms PSD in hippocampal CA1 region of WT/T2KO mice and Floxed/neuron-specific T2KO mice. n = 8, *** p ≤ 0.001. (**H**) and (**I**) TPP2 depletion doesn't cause significant difference of the mean resultant vector length (MRL) of theta and gamma oscillations in the hippocampus of 6 month-old female mice. The upper panel showing the circular distribution of the mean theta- or gamma-phase event spike values computed as angles recorded from CA1 area of WT (**Left**) and T2KO (**Middle**) mice (15° bin width), and the red bar in the circle displaying the direction and magnitude of the population. The lower panel showing the distribution of mean-spike theta- or gamma-phase angles (45° bin width), and the red curve displaying one schematic theta- or gamma-cycle. The small circle position representing the mean phase angle and the bars on the small circle showing the cell numbers. WT: n = 15 neurons from 5 mice; T2KO: n = 24 neurons from 8 mice, **^ns^** p > 0.05. (**J**) TPP2 depletion impairs phase-amplitude cross-frequency coupling between theta and gamma oscillations in the hippocampus of 6 month-old female mice. Modulation index comparison between WT (n = 5) and T2KO (n = 10) female mice. *p< 0.05.

Based on above results, we further tested the learning and memory abilities of 6 month-old TPP2 knockout mice and excitatory neuron-specific TPP2 knockout mice. Our data showed that the escape latency of male mice over 5 days is significantly decreased in both WT and TPP2 knockout groups, whereas the escape latency of female mice over 5 days becomes gradually shorter only in the WT group, it even gradually becomes longer in TPP2 knockout group over the time (Figure 10A). Interestingly, the conditional knockout mice also exhibit similar learning and memory phenotypes, namely, compared to 2-month-old female mice, 6-month-old female mice do not show any improvement in learning and memory abilities, while male mice do show clear learning and memory ability (Figure 10B). In consistence with these findings, our electrophysiological analysis of brain slices showed that the LTP in the dentate gyrus (DG)-cornu ammonis 3 (CA3) or CA3-CA1 connection of female mice is significantly attenuated not only under ubiquitous TPP2 depletion but also under neuron-specific TPP2 depletion, although the degree of attenuation of the conditional depletion is not as large as that of ubiquitous TPP2 knockout (Figure 10C). Furthermore, our in-vivo electrophysiology recording with microwire array electrode for analysis of LFPs (≤ 300Hz) in hippocampal CA1 region of 6 month-old mice showed that both ubiquitous and neuron-specific TPP2 depletion cause markedly reduced amplitude of LFPs and gamma-theta rhythms as compared with that of WT mice, although the reduction under neuron-specific TPP2 depletion is not as much as that in ubiquitous TPP2 knockout mice (Figure 10D-G). LFPs reflect neural mass action across brain regions and numerous studies have characterized the properties of LFP signals in the cortex to study cognitive processes like memory, attention, and perception as well as sensory and motor computations 130. In particular, both Gamma and Theta oscillations are crucial for cognitive processing, especially for learning and memory 131, 132. In response to above findings the following phase locking assays showed that, although TPP2 depletion doesn't cause significant difference of the mean resultant vector length (MRL) of theta and gamma oscillations in the hippocampus (Figure 10 H-I), it indeed impairs phase-amplitude cross-frequency coupling between theta and gamma oscillations in the hippocampus (Figure 10J).

### TPP2 depletion impairs adult neurogenesis in the hippocampal DG of adult female mice, which can be rescued by injection of AAV harbouring aromatase expression cassette

**Figure 11:**
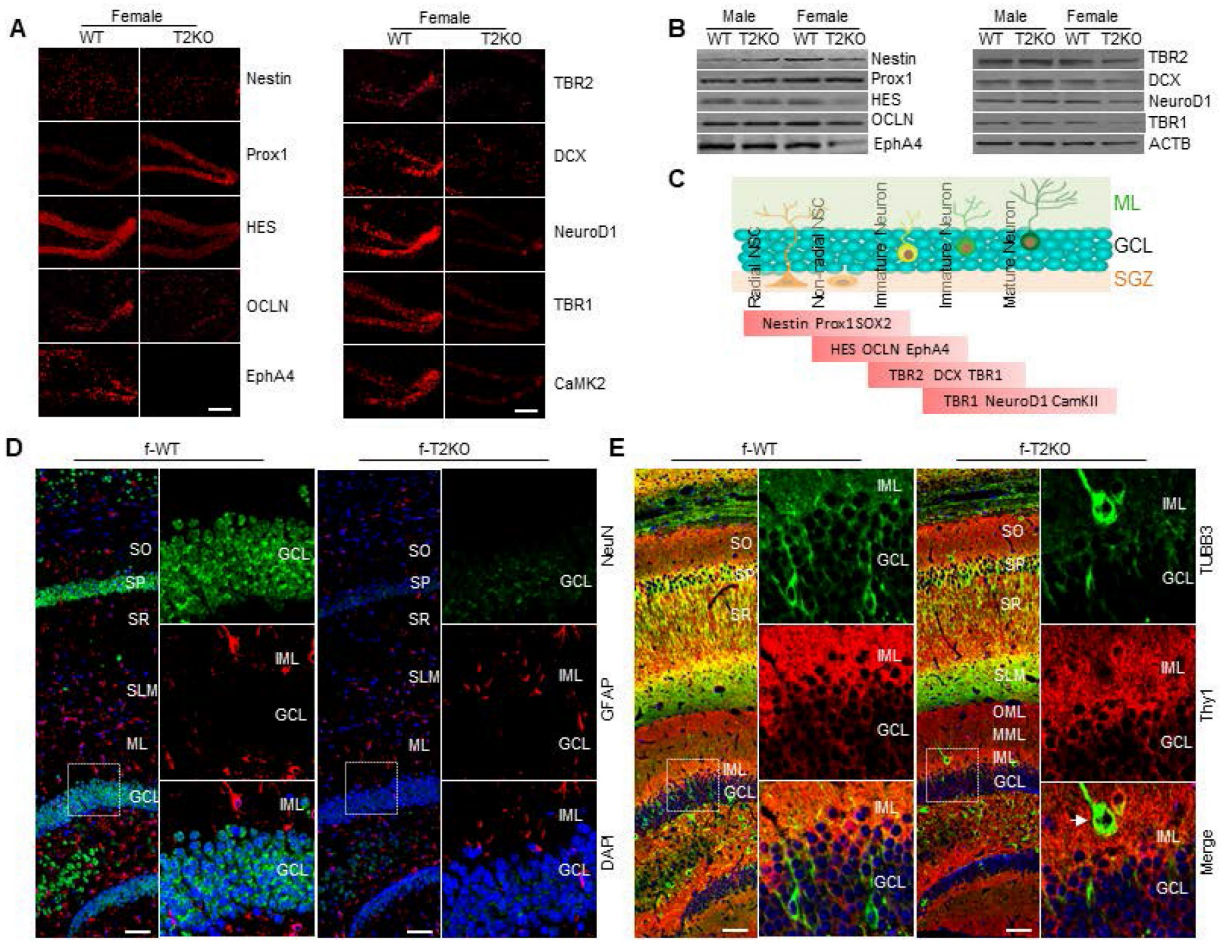
TPP2 depletion impairs adult neurogenesis in the hippocampal DG of female mice. (**A**) IHC analysis of the stage markers of adult neurogenesis in hippocampal DG of WT and TPP2 knockout female mice (p300). The immunoreactivities of all the other markers except Prox1 are significantly reduced in hippocampi of female TPP2 knockout mouse when compared with WT counterpart. One representative holographic scanning of the hippocampus is shown. Brain slice from the same brain area of 6 different mice at the same age were used for staining in each case. Scale bars, 100 μm. (**B**) Immunoblot to examine the stage markers of adult neurogenesis in hippocampal DG of WT and TPP2 knockout mouse (p300). The data showed that, being consistent with the abovementioned immunostaining result, the levels of all the other markers except Prox1 are significantly reduced in hippocampi of female TPP2 knockout mouse when compared with WT counterpart, whereas that of male mice has no significant difference between WT and TPP2 knockout mice. One representative blot result from 3 repeated experiments is shown. (**C**) Diagram illustrating the stages of adult neurogenesis in the rodent hippocampal DG. The quiescent radial glial-like neural stem cells (NSCs) upon activation proliferate in the SGZ at the border of the GCL and form non-radial daughter NSC cells, thereby initiating adult neurogenesis. The non-radial NSCs proliferate to expand themselves and differentiate to neuroblasts (immature neuron). A fraction of these neuroblasts migrate into the GCL where they go on to mature granule neurons and send out dendrites into the molecular layer (ML) and axons to the CA3 region via the mossy fiber pathway. Listed are the notable stage markers of adult neurogenesis. (**D**) IHC analysis of the neuronal marker NeuN and astrocytic marker GFAP in hippocampal DG of WT and TPP2 knockout female mice (p300). The immunoactivity of NeuN is significantly reduced in hippocampus of female TPP2 knockout mouse when compared with WT counterpart, while that of GFAP has no clear difference between TPP2 knockout and WT counterpart. One representative slice of the hippocampus is shown. The square region in the image is enlarged showing the expression of NeuN (Alexa Fluor 488, Green), GFAP (Alexa Fluor 594, Red) and merged with DAPI (Blue). Brain slice from the same brain area of 6 different mice at the same age were used for staining in each case. Scale bars, 50 μm. GCL: granule cell layer; ML: molecular layer of dentate gyrus; SLM: stratum lacunosum; SR: stratum radiatum; SP: stratum pyramidale; SO: stratum oriens; IML: inner molecular layer; MML: middle molecular layer; OML: outer molecular layer. (**E**) IHC analysis of the earliest neuronal marker TUBB3 and the intermediate marker of NSC and neuron Thy1 in hippocampal DG of WT and TPP2 knockout female mice (p300). The overall immunoactivity of TUBB3 is significantly reduced in hippocampi of female TPP2 knockout mouse when compared with WT counterpart, whereas some mistargeted neurons have extreme high level of TUBB3 in hippocampus of TPP2 knockout mouse. By contrast, the overall immunoactivity of Thy1 has no clear difference in hippocampal DG of WT and TPP2 knockout mice. One representative slice of the hippocampus is shown. The square region in the image is enlarged showing the expression of TUBB3 (Alexa Fluor 488, Green), Thy1 (Alexa Fluor 594, Red) and Merged with DAPI (Blue). Brain slice from the same brain area of 6 different mice at the same age were used for staining in each case. Scale bars, 50 μm. GCL: granule cell layer; ML: molecular layer of dentate gyrus; SLM: stratum lacunosum; SR: stratum radiatum; SP: stratum pyramidale; SO: stratum oriens; IML: inner molecular layer; MML: Middle molecular layer; OML: outer molecular layer.

The regulation of brain function and behavior by steroid hormones is usually related to the secretion of peripheral endocrine glands. However, for women, there is a significant decrease in estrogen secretion after menopause 133, 134. Perhaps, due to compensation for this deficiency, the brain has evolved a de novo steroidgenesis pathway to synthesize steroid hormones, which not only control reproductive behavior but also participate in various non reproductive functions. These include the regulation of neurogenesis, neuronal development, synaptic transmission and plasticity, regulation of reactive astrocyte and microglia activation, and anti-inflammatory effects 135, 136. Considering that TPP2 depletion can lead to abnormal degradation of aromatase and insufficient estrogen production, we examined the stage markers of adult neurogenesis in hippocampal DG of 10 month-old TPP2 knockout female mice and found that all the other markers except prox1 are significantly reduced in TPP2 knockout female mouse hippocampus as compared with WT control (Figure 11A-C). In line with these findings, IHC analysis of the neuronal marker Neuronal nuclear antigen (NeuN) and astrocytic marker Glial fibrillary acidic protein (GFAP) in hippocampal DG of TPP2 knockout female mice showed that the immunoactivity of NeuN is significantly reduced in the hippocampus of TPP2 knockout female mouse as compared with WT animal, while that of GFAP has no clear difference between TPP2 knockout and WT counterpart (Figure 11D). Meanwhile, IHC analysis of the earliest neuronal marker tubulin beta 3 class III (TUBB3) and the intermediate marker of NSC and neuron Thy1 in hippocampal DG of TPP2 knockout female mice showed that the overall immunoactivity of TUBB3 is significantly reduced in TPP2 knockout female mouse hippocampus as compared with WT counterpart, whereas some mistargeted neurons have extreme high level of TUBB3 in TPP2 knockout hippocampus. By contrast, the overall immunoactivity of Thy1 has no clear difference in hippocampal DG of WT and TPP2 knockout mice (Figure 11E). Taken together, these data showed that TPP2 depletion causes severe impairment of adult neurogenesis in female mice, which is in consistence with the attenuated LTP and reduced learning and memory abilities of TPP2 knockout female mice.

### TPP2 depletion impairs synaptogenesis in the hippocampal DG of adult female mice

**Figure 12:**
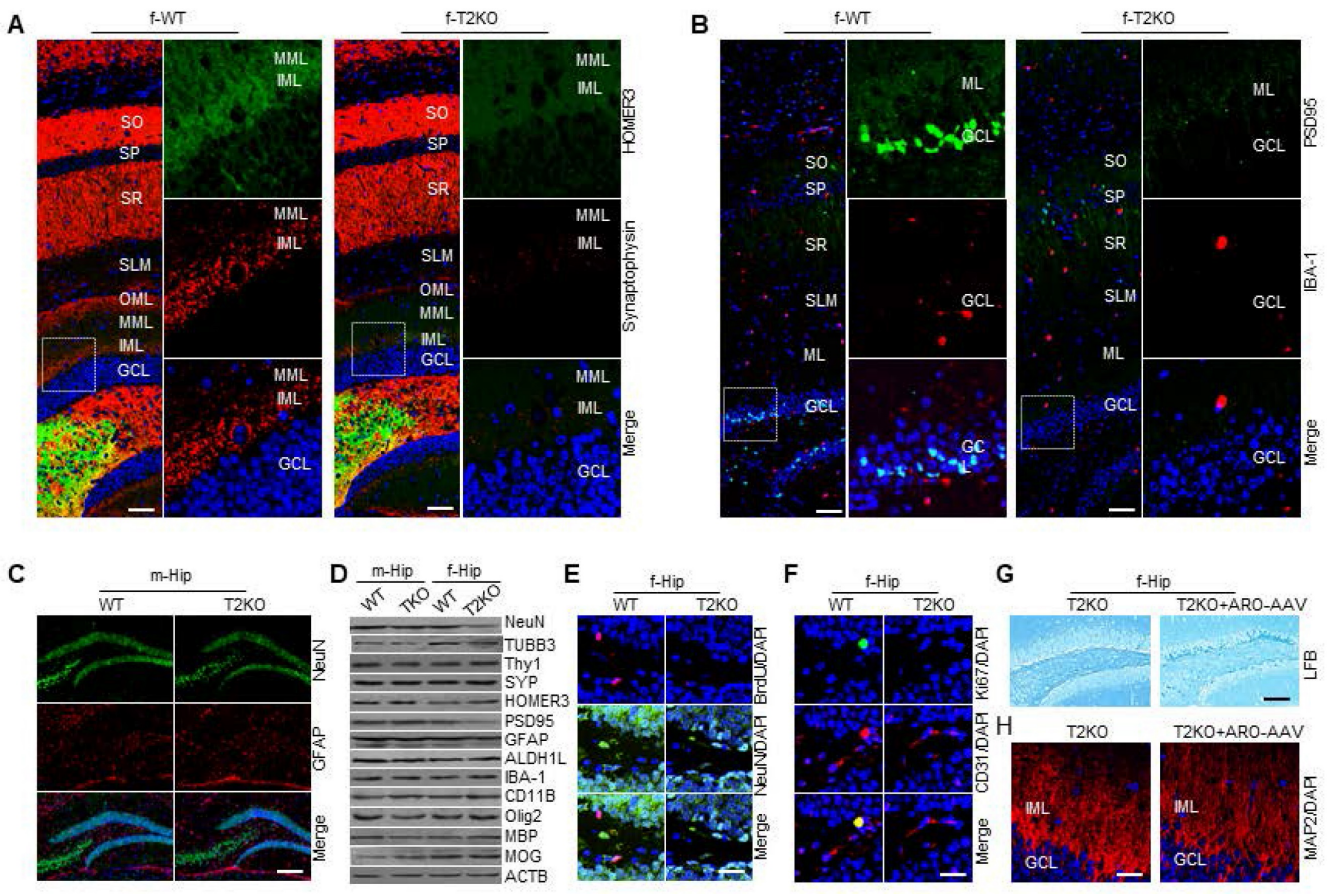
TPP2 depletion impairs synaptogenesis in the hippocampal DG of adult female mice. (**A**) IHC analysis of the presynaptic marker SYP and postsynaptic marker homer in hippocampal DG of female WT and TPP2 knockout mice (p300). These results showed that the immunoreactivities of SYP and homer in hippocampal molecular layer (ML) of TPP2 knockout mouse are significantly reduced when compared with WT counterparts, although that of homer in CA3 is not significantly changed. The square region in the image is enlarged showing the expression of homer (Alexa Fluor 488, Green), SYP (Alexa Fluor 594, Red) and merged with DAPI (Blue). One representative holographic scanning of the immunostained hippocampus is shown. Brain slice from the same brain area of 6 different mice at the same age were used for staining in each case. Scale bars, 50 μm. GCL: Granule cell layer; ML: molecular layer of dentate gyrus; SLM: stratum lacunosum; SR: stratum radiatum; SP: stratum pyramidale; SO: stratum oriens; IML: inner molecular layer; MML: middle molecular layer; OML: outer molecular layer. (**B**) IHC analysis of the postsynaptic marker PSD95 and microglial marker IBA-1 in hippocampal DG of female WT and TPP2 knockout mice (p300). These data showed that the immunoreactivity of PSD95 is significantly reduced in hippocampal GCL and ML of TPP2 knockout mouse when compared with WT counterpart. By contrast, that of IBA-1 has no significant change between TPP2 knockout ant WT counterparts. The square region in the image is enlarged showing the expression of PSD95 (Alexa Fluor 488, Green), IBA-1 (Alexa Fluor 594, Red) and merged with DAPI (Blue). One representative holographic scanning of the immunostained hippocampus is shown. Brain slice from the same brain area of 6 different mice at the same age were used for staining in each case. Scale bars, 50 μm. GCL: granule cell layer; ML: molecular layer of dentate gyrus; SLM: stratum lacunosum; SR: stratum radiatum; SP: stratum pyramidale; SO: stratum oriens; IML: inner molecular layer; MML: middle molecular layer; OML: outer molecular layer. (**C**) IHC analysis of NeuN and GFAP in hippocampi of male WT and TPP2 knockout mice (p300). These data showed that the immunoactivities of NeuN and GFAP in the hippocampi of WT and TPP2 knockout mouse present no difference. One representative staining result is shown with NeuN (Alexa Fluor 488, Green), GFAP (Alexa Fluor 594, Red) and merged with DAPI (Blue). Brain slice from the same brain area of 6 different mice at the same age were used for staining in each case. Scale bars, 100 μm. (**D**) Immunoblot for analysis of the markers of neural cells in hippocampi of WT and TPP2 knockout mice (p300). The results showed that there is no difference in the levels of all the tested markers between male WT and TPP2 knockout mice. However, there are significant differences in NeuN, TUB3, and PSD95 levels between female WT and TPP2 knockout mice. These observations combined with immunostaining results showed that TPP2 depletion causes clear impairment of adult neurogenesis in females. One representative blot result from 3 repeated experiments is shown. (**E**) BrdU assay to examine adult neurogenesis in hippocampal DG of female WT and TPP2 knockout mice (p300). These data showed that BrdU incorporation is more pronounced in WT than in TPP2 knockout hippocampus, and correspondingly the immunoreactivity of endogenous neuronal marker NeuN is also more pronounced in WT than in TPP2 knockout hippocampus. These observations indicate that TPP2 depletion causes neurogenesis deficiency in adult female mice. One representative immunostaining result from 6 independent experiments is shown. Scale bars, 25 μm. (**F**) IHC staining of Ki67 and CD31 for analysis of adult neurogenesis in hippocampal DG of female WT and TPP2 knockout mice (p300). These data showed that the immunoreactivities of Ki67 and CD31 in TPP2 knockout hippocampus become significantly decreased as compared with WT counterparts. These data confirm that TPP2 causes neurogenesis deficiency in adult female mice. One representative immunostaining result from 6 independent experiments is shown. Scale bars, 25 μm. (**G**) Histological LFB staining to examine axon myelination in hippocampal DG of female TPP2 knockout mice (p300). The data showed that the cells in the SGZ of TPP2 knockout mice, especially those in the dorsal hippocampal DG, are myelination deficient, although immunoblot does not show clear decrease of MBP and MOG (shown in D), and this can be rescued by injection of AAV2/BBB vector harbouring aromatase expression cassette. Scale bars, 100 μm. (**H**) IHC staining of MAP2 to examine dendrite morphogenesis in hippocampal DG of female TPP2 knockout mice (p300). This data showed that dendrites in IML of TPP2 knockout mice are not a collection of highly branched, tapering processes. Instead, they don't have clear contours and generally look messy, which can be rescued by injection of AAV2/BBB vector harbouring aromatase expression cassette. Scale bars, 20 μm.

Neurogenesis is the process of producing neurons from neural stem cells and progenitor cells, while synaptogenesis is the formation of synapses between neurons in the nervous system. Given that TPP2 depletion causes severe impairment of adult neurogenesis in female mice, we further examined the synaptogenesis in these female mice. We have analyzed the presynaptic marker synaptophysin (SYP) and postsynaptic marker homer in hippocampal DG of 10 month-old TPP2 knockout female mice. The data showed that the immunoreactivities of SYP and homer in hippocampal molecular layer (ML) of TPP2 knockout mouse are significantly reduced as compared with WT counterparts, although that of homer in CA3 is not significantly changed (Figure 12A). Meanwhile, we also examined the postsynaptic marker Postsynaptic density protein 95 (PSD95) and microglial marker Ionized Ca^2+^-binding adapter molecule 1 (IBA-1) in hippocampal DG of TPP2 knockout female mice. These data showed that the immunoreactivity of PSD95 is significantly reduced in hippocampal GCL and ML of TPP2 knockout mouse as compared with WT counterpart. By contrast, that of IBA-1 has no significant change between TPP2 knockout ant WT counterparts (Figure 12B).

Our data clearly showed that TPP2 causes impairment of adult neurogenesis and synaptogenesis in female mice. To validate these findings, we examined the adult neurogenesis in 10 month-old TPP2 knockout male mice. Our data showed that the immunoactivities of NeuN and GFAP in the hippocampi of TPP2 knockout male mouse have no significant difference as compared with WT control (Figure 12C). We compared the levels of these markers in male and female mouse hippocampi by immunoblot, and the results showed that TPP2 depletion doesn't cause clear changes of all the tested markers in male mice, whereas it does cause great changes of NeuN, TUBB3 and PSD95 in female mice as compared with WT controls (Figure 12D). In consistence with these findings, our BrdU assay showed that BrdU incorporation is significantly less pronounced in the hippocampi of TPP2 knockout female mice as compared with WT mice (Figure 12E), and similarly the immunoreactivities of Ki67 and CD31 in TPP2 knockout hippocampus also become significantly decreased as compared with WT counterparts (Figure 12F). In addition, we examined axon myelination of hippocampal DG neurons in TPP2 knockout female mice by LFB staining. The data showed that cells in the subgranular zone (SGZ) of TPP2 knockout mice, especially those in the dorsal hippocampal DG, are demyelinated (Figure 12G), although immunoblot does not show clear decrease of myelin basic protein (MBP) and myelin oligodendrocyte glycoprotein (MOG) (Figure 12D). In particular, this can be rescued by injection of AAV2/BBB vector harbouring aromatase expression cassette (Figure 12G). The dendrites in IML of TPP2 knockout female mice are not a collection of highly branched, tapering processes. Instead, they don't have clear contours and generally look messy, which can also be rescued by injection of AAV2/BBB vector harbouring aromatase expression cassette (Figure 12H).

## Discussion

TPP2 is generally considered as an ubiquitously expressed cytosolic serine peptidase, which forms the largest known protease complex in eukaryotic cells to operate mostly downstream of proteasomes for extralysosomal peptide degradation 1-4. However, this canonical function of TPP2 cannot explain its role in a large number of important biological processes, and also the mechanical interrelationships and hierarchical order of these processes still need to be clarified. In this study, we found an enzymatic activity independent function of TPP2 using TPP2 knockout 293T cells and ubiquitous/excitatory neuron-specific TPP2 knockout mice. We showed that TPP2 gene ablation leads to sexually dimorphic intracellular Ca^2+^ dyshomeostasis, PC deficit, and ER stress. These abnormalities cause ATF6-SYVN1-UCHL1 axis-mediated autophagic aromatase degradation and estrogen deficit, culminating in presenile dementia of both ubiquitous and excitatory neuron-specific TPP2 knockout female mice. This study therefore discloses a new function of TPP2 which not only makes great sense for elucidating the pathogenesis and future treatment of dementia, but also for interpreting the role of TPP2 in other system and treatment of the related disorders.

Ca^2+^ is a highly versatile intracellular messenger that impacts, from fertilization to cell death, nearly every aspect of cellular life 137, 138. It is of particular importance to neurons as it participates in and regulates a wide variety of important neurobiological processes such as transmission of the action potential, neuronal energy metabolism and dendrite development, synaptogenesis and plasticity etc. 139, 140. As such, Ca^2+^ homeostasis is of pivotal interest for neural cells and its dysregulation is related to a series of neurodegenerative diseases including Alzheimer's, Parkinson's, and Huntington's diseases 141, 142. In general, cellular Ca^2+^ homeostasis is maintained through the integrated and coordinated function of Ca^2+^ transporters, buffers, and sensors, which are distributed in differerent cellular compartments such as ER, mitochondria, cytoplasm, and plasma membrane 143, 144. Here, we show that TPP2 is an important Ca^2+^ regulator independent of its peptidase activity. TPP2 depletion leads to an increase of Ca^2+^ concentration in ER along with a decrease in cytoplasm of 293T cells (originating from a female fetus) and hippocampal neurons of female mice, and both of which can be reversed through ectopic expression not only of TPP2 but also of the enzymatic activity deficient mutant TPP2 S449A or S449T (Figure 1).

As for the underlying mechanism, we show that TPP2 interacts with IP3R1, VDAC2, SERCA1, ATF6, IRE1 and PERK, and TPP2 depletion disrupts the interaction between IP3R1 and VDAC2 in MAMs and the inhibitory interaction between SERCA1 and PLN, while enhance the inhibitory interaction between IP3R1 and CALR (Figure 2-3). As disruption of the interaction between IP3R1 and VDAC2 blocks Ca^2+^ transfer from ER to mitochondria, disruption of the interaction between SERCA1 and PLN promote ER Ca^2+^ refilling from cytoplasm via SERCA1, and enhancement of the interaction between IP3R1 and CALR prevents ER Ca^2+^ release to cytoplasm via IP3R1, it is conceivable that these combined effects will lead to a decrease in cytosolic Ca^2+^, an increase in ER Ca^2+^, and a decrease in mitochondrial Ca^2+^ under TPP2 depletion. Although ATF6, IRE1, and PERK regulate intracellular Ca^2+^ homeostasis too 35, 145, 146, and our data also showed that knockout of each of them enhances the interactions between TPP2 and IP3R1, SERCA1 and VDAC2 (Figure 2C), for some reason, additional depletion of any of them neither aggravates nor alleviates the Ca^2+^ dysregulation caused by TPP2 depletion alone. Considering that ER is the major store for Ca^2+^ in the cell, and under physiological conditions, a steep concentration gradient between the ER (100 μM up to 1 mM [Ca^2+^]) and the cytosol (about 100 nM [Ca^2+^]) is mainly achieved by SERCA for ER Ca^2+^ refilling and IP3Rs for ER Ca^2+^ release 144, 147, 148, it appears that TPP2 depletion causes Ca^2+^ dysregulation mainly through interfering with the funtions of SERCA1, IP3R1 and VDAC2. It is noteworthy that all of IP3R, SERCA, and VDAC have different isoforms 30, 149, 150. Interestingly, up to now, we haven't found interactions between TPP2 and other isoforms except IP3R1, SERCA1, and VDAC2 (data not shown).

As reported, Ca^2+^ and lipid metabolism mutually regulate each other 48, 49. However, the detailed mechanisms are not yet clear. In this study, we show that TPP2 controls the biosynthesis of PC via regulation of Ca^2+^. TPP2 depletion causes a decrease of Ca^2+^ in cytosol and an increase inside ER, these in turn prevent the assembly of catalytic enzyme complex for PC synthesis on the ER membrane, and transient exposure of TPP2 knockout cells or neurons to TG to prevent ER Ca^2+^ refilling can restore the assembly of catalytic enzyme complex for PC synthesis on the ER membrane of TPP2 knockout cells. Although it has been shown that incubation of rat heptocytes with millimolar scale of Ca^2+^ in the medium can stimulate CCT to translocate from cytosol to microsomes 50, our findings revealed a novel mechanism of CCT translocation in CNS, which not only depends on the level of Ca^2+^ level in the niche but also on downstream enzymes in the target membrane. Notably, the current prevailing perception is that CCTα can sense the compositional PC defects in membrane and then become activated for biosynthesis of PC after selectively binding to certain lipid membranes 53, 65, 151. However, biochemical and structural studies on purified CCTα have revealed that CCTα doesn't form specific complexes with the surface lipids, but rather its C-terminal amphipathic helix (M domain) is adsorbed on permissive lipid surface to relieve its auto-inhibition of enzymatic activity 65, 151. It was shown that many features, including not only membrane curvature strain but also membrane electrostatics, affect M domain adsorption 53, 152. As abovementioned, we found that CCTα distribution on ER membrane is significantly decreased under TPP2 depletion and transient exposure to TG can completely restore its distribution on ER membrane (Figure 4A-B). In particular, we discovered that CEPT1 plays a key role in recruiting CCTα to ER membrane (Figure 4C-E). It appears that Ca^2+^ improves CCTα adsorption to ER membrane via CEPT1 through as yet unknown mechanism. In consistence with this notion, the levels of 13 PC species with saturated fatty acid chains decrease in 6 month-old TPP2 knockout mouse brains (Figure 5A). However, the levels of 3 PC species with longer and unsaturated fatty acid chains increase under TPP2 depletion (Figure 5A). As for this, we revealed that, in contrast to decrease of the interaction between CCTα and CEPT1, the interaction between CCTα and CPT1 increases under TPP2 depletion. As CEPT1 is ER-localized, while CPT1 is TGN-localized and is more inclined to synthesize PC with unsaturated chains, different lipid compositions and biophysical properties of TGN and ER membrane may contribute to the membrane binding of CCTα for biosynthesis of different species of PC 19, 71-73, 153. Nonetheless, according to our findings, it is clear that Ca^2+^ plays an important role in recruiting CCTα to CEPT1 or CPT1 in target organelle membrane to promote or prevent CCTα activation in context dependent manner, though the detailed mechanism is not completely understood. As for the decrease of other lipids under TPP2 depletion, they most probably stem from PC deficit 20, 74, 75. In agreement with this notion, while PE promotes CCTα membrane binding, LPC, ceramide, and PC itself prevent CCTα membrane binding 154-157. Taken together, our data clearly indicate that TPP2 maintains lipidomics homeostasis in the CNS mainly through adjusting the assembly of enzymatic complexes for PC synthesis in organelle membrane in a Ca^2+^ and niche dependent manner, though the mechanism is up to now not entirely unveiled, which deserves further profound exploring.

Both Ca^2+^ dyshomeostasis and PC dysregulation lead to ER stress 21-23, 76. In this study we showed that TPP2 depletion causes Ca^2+^ dyshomeostasis, PC dysregulation, and ER stress (Figure 1, 5-6). In addition, we found that Ca^2+^ and PC regulate each other in a positive feedback manner (Figure 4). In particular, we discovered that, when CCTα is depleted, transient treatment with TG to upregulate cytosolic Ca^2+^ concentration fails to rescue TPP2 depletion-induced ER stress (Figure 6). These observations suggest that TPP2 depletion-induced Ca^2+^ dyshomeostasis and PC dysregulation are very tightly conjugated, and their synergistic effect causes ER stress. As known, ER is the main Ca^2+^ storage organelle, and ER Ca^2+^ not only provides a favorable environment for protein folding but also a source of Ca^2+^ for cell signaling 147, 158. As such, it is conceivable that the ER Ca^2+^ content must be tightly controlled and altered ER homeostasis can impact a myriad of cellular processes and therefore lead to ER stress. However, our above findings that TPP2 depletion-induced Ca^2+^ dyshomeostasis leads to ER stress through mainly causing CCTα to malfunction suggest TPP2 depletion-induced ER stress is ultimately caused by PC deficit. It has been shown that IRE1 and PERK sense generalised LBS at the ER, while ATF6 is activated by altered specific sphingolipids 23. In agreement with this notion, TPP2 depletion causes dyshomeostasis of PCs and ceramides and activates all three branches of UPR (Figure 5-6). To validate TPP2 depletion-induced ER stress, we have examined the expression levels of ER stress-related proteins and the levels of free amino acids in TPP2 knockout 293T cells by immunoblot and LC-MS/MS, respectively. Our data clearly showed that TPP2 depletion leads to ER stress, which is characterized by cleavage of p90ATF6, IRE1 autophosphorylation, ATF4 overexpression, upregulation of GRP78, PDI, ERO1L, and UGGT1, and enrichment of ATF4-dependent UPR amino acid signatures (Figure 6) 99, 101. Of note, it was reported that the overall intracellular free amino acids are normal to higher in TPP2 knockout MEF cells or when MEF cells were exposed to Butabindide 6 to 10 h as compared with controls, whereas transient exposure to Butabindide caused amino acid depletion 159. By comparison, our data showed that the ATF4-dependent UPR signature amino acids are selectively increased and other amino acids become decreased in TPP2 knockout 293T cells (Figure 6D). The discrepancy between our data and the published data may be due to different cells used for experiments. As known, 293T cell line is originally from an aborted human female embryo and has been widely used in the fields of research and industry 160, 161. In consistence with the data acquired from 293T cells, TPP2 depletion leads to sexually dimorphism in both Ca^2+^ dyshomeostasis and PC dysregulation in hippocampal neurons. By the way, according to reports, ER stress upregulates expression of genes that carry out glycolysis while downregulates expression of genes that participate in mitochondrial respiration through ATF4 77, 162. Consistently, we found that the top 10 enriched GO BPs of the upregulated proteins in TPP2 knockout 293T cells are related to glycolysis and NADH/NAD metabolism (Figure S1B). Thus, we believe that upregulation of glycolysis is secondary effect of TPP2 depletion-induced ER stress.

In line with the findings that TPP2 depletion causes sexual dimorphism in Ca^2+^ dyshomeostasis, PC dysregulation, and ER stress, and that all three branches of UPR signaling pathways are activated, we further found that TPP2 depletion causes in situ apoptosis and autophagy of hippocampal neural cells accompanied by testosterone accumulation and estrogen diminution in the brains of 6 month-old female mice. Particularly, 6 month-old TPP2 knockout female mice showed significantly increased apoptotic staining and autophagy marker staining in GCL of hippocampal DG region as compared to control animals. Interestingly, neither 6 month-old nor 10 month-old TPP2 knockout female mice showed premature senescent staining in GCL of hippocampal DG region as compared to control animals. Of note, premature senescence in other systems has been reported in TPP2 knockout mice 3, 5. That there is no premature senescence in hippocampal SGZ of TPP2 knockout mice may reflect the tissue-specific roles of TPP2. Generally, ER stress stimulates autophagy via such as ATF4 to orchestrate the recuperation of ER function besides UPR 163-166. However, chronic ER stress can overwhelm these adaptive changes and may eventually commit cells to degeneration or death such as apoptosis 167, 168. In contrast to above notion, it has also been reported that apoptosis induces autophagy, and autophagy in turn blocks apoptosis 104, 105. Clearly, either autophagy or apoptosis is one of the responses the cells choose during ER stress. As for which response is the initial one or the second one, we believe it depends on the context. Given that TPP2 depletion causes both apoptosis and autophagy of hippocampal DG neural cells of female mice and that sexual hormones, especially estrogen, have been shown to have very important protective effects on the nervous system 106-108, we postulated that TPP2 knockout female mice may have insufficient estrogen. As expected, our LC-MS/MS-based metabolomics analysis of sex hormones showed that both estrone and estradiol significantly decrease in the brains of TPP2 knockout adult female mice as compared with control animals, while its precursors and metabolites increase (Figure 7). These results indicate that both production and degradation of estrogens are defective in TPP2 knockout adult female mice. Added to that, we found that aromatase for estrogen production is significantly downregulated, while COMT for estrogen decomposition is significantly upregulated in TPP2 knockout hippocampi of adult female mice (Figure 8). These observations suggest that TPP2 depletion-induced apoptosis and autophagy of hippocampal DG neural cells of female mice are attributed to insufficient local estrogen synthesis and aberrant decomposition. Consistent with this viewpoint, we also found that in the case of TPP2 depletion, aromatase is abnormally degraded by the autophagy pathway, rather than its transcriptional reduction. Most interestingly, the autophagic degradation of aromatase can be rescued by ectopic expression of non-enzymatic TPP2 mutants or preincubation with PC, and single intravenous injection of AAV expressing aromatase significantly rescues the learning and memory impairment of female TPP2 knockout mice and prevents their hippocampal neural cell death. To sum up, our data implicate that ER stress-induced autophagic degradation of aromatase and subsequent decrease of estrogen production precede apoptosis of hippocampal neural cells in adult TPP2 knockout female mouse brains. It is noteworthy that increased level of COMT under TPP2 depletion can accelerate estrogen degradation, which may contribute to estrogen depletion and subsequent neural cell death 169, 170. However, our AAV-aromatase rescue experimental result showed that COMT plays an inferior role in TPP2 depletion-induced neural cell death as compared with aromatase. Nevertheless, it is conceivable that upregulation of COMT and abnormal aromatase degradation may play a synergistic role in inducing neural cell death. Due to limited space in this article, there is no further research on this topic. However, so far, the data we have obtained showed that aromatase, rather than COMT, is physically associated with ATF6 under TPP2 depletion, although both aromatase and COMT are mainly located in ER membrane 125, 171. As aforementioned, ATF6 is one of the interaction partners of TPP2, and it plays an organizer's role in autophagic degradation of aromatase under TPP2 depletion. However, the mechanism underlying this non-canonical function of ATF6 is not completely understood. It seems that Ca^2+^ dyshomeostasis-induced PC deficit provides the conditions for or directly promotes the binding of ATF6 with aromatase and other autophagy related proteins for autophagosome formation, because the autophagic degradation of aromatase can be prevented by adding PC (Figure 9A). Considering that adding PC also restores the interaction between ATF6 and GRP78 and the latter has been shown to be an autophagy activator 164, it is reasonable to believe that the autophagic degradation of aromatase is one of the ATF6-related ER stress responses, whose canonical role is as a transcription factor after cleavage in Golgi body. In particular, the interaction between ATF6 and aromatase under TPP2 depletion is almost completely abolished by phospho-inhibiting S118A mutation (Figure 9C). Taken together, these observations suggest that, under TPP2 depletion, phosphorylation of S118 of aromatase and decoupling of ATF6 from GRP78 synergistically enhance the interaction between aromatase and ATF6 and thereforth its interaction with p62, and ultimately lead to aberrant autophagic aromatase degradation. This non-canonical function of ATF6 is discovered first time by us, and as part of an important branch of UPR deserves further investigation.

Given that TPP2 depletion causes sexual dimorphism in Ca^2+^ dyshomeostasis, PC dysregulation, ER stress, and autophagic degradation of aromatase, we compared the learning and memory abilities of 6 month-old male and female TPP2 knockout mice and excitatory neuron-specific TPP2 knockout mice. We found that both ubiquitous and excitatory neuron-specific depletion of TPP2 cause learning and memory deficiency only in female mice. Furthermore, ubiquitous TPP2 depletion causes more severe memory loss than excitatory neuron-specific depletion of TPP2. Consistently, the LTP in DG-CA3 or CA3-CA1 connection of female mice is significantly attenuated under both ubiquitous and neuron-specific TPP2 depletion, but the degree of attenuation of the conditional depletion is not as large as that of ubiquitous TPP2 knockout (Figure 10C). In addition, the amplitudes of LFP and gamma-theta rhythm of female mice are markedly reduced under both ubiquitous and neuron-specific TPP2 depletion, but the reduction under neuron-specific TPP2 depletion is not as much as that in ubiquitous TPP2 knockout mice (Figure 10D-F). As known, LFPs, especially Gamma and Theta oscillations in the cortex, are crucial for learning and memory 131, 132. We have measured LFP in hippocampal CA1 region combined with LTP of CA3-CA1 and DG-CA3. The dataset of LTP and LFP strongly supports loss of learning and memory abilities of adult TPP2 knockout female mice. Notably, although both LTP and LFP under excitatory neuron-specific TPP2 depletion are severe enough to cause impairment of learning and memory in adult female mice, they two are less attenuated as compared with ubiquitous TPP2 depletion. These data implicate that there exist other factors in ubiquitous TPP2 knockout female mice, which can exacerbate conditional TPP2 depletion-induced impairment of learning and memory. For example, CA1 parvalbumin-expressing inhibitory neurons (PV-INs) contribute to brain estradiol synthesis, and in the female brain, aromatase modulates PV-INs activity, the dynamics of network oscillations and hippocampal-dependent memory 172. Indeed, induced expression of TPP2 in excitatory neurons of our TRE-TPP2 knockin mouse model (Figure S4) could only partially rescue the learning and memory abilities of ubiquitous TPP2 knockout mice (data nor shown). In addition, astrocytes in brain were reported to produce estrogen 135. Thus, we have prepared astrocyte and also neural stem/progenitor cell (NSPC) specific TPP2 knockout mice and found that the 6 month-old adult female mice of both conditional knockout lines have clear impairment in learning and memory, though not as severe as ubiquitous TPP2 knockout mice (Data not shown). This NSPC specifc TPP2 depletion-induced dimorphic phenotype in learning and memory indicate NSC may be another important source of estrogen in adult female mice. In fact, our data showed that aromatase is highly expressed in hippocampal SGZ of adult female mice (Figure 8C). Patkar et al also showed that aromatase is expressed in NSC and knockdown of Dax-1 can induce its overexpression 173. As such, it is understandable that ubiquitous TPP2 depletion causes more severe impairment in learning and memory abilities than conditional TPP2 depletion, because all sources of estrogen may be damaged under ubiquitous TPP2 depletion.

Mammalian brains are equipped with all the enzymes required for neurosteroidogenesis, and aromatase is widely expressed in the brains of female mammals 174-176. In particular, its expression in hippocampus has been intensively studied 172, 177, 178, because it catalyzes the local production of estrogen, that has been shown to play an essential role in adult neurogenesis, neuroprotection, synaptic plasticity, and anti-inflammation etc. through its function as anti-oxidants, promoting DNA repair, inducing the expression of growth factors, and modulating cerebral blood flow 135, 179-183. In agreement with these observations, in this study, we found that TPP2 gradually decreases with age in mouse CNS (Figure S1D) and its depletion causes aberrant degradation of aromatase and subsequent estrogen deficit in adult female mouse hippocampi (Figure 8 and 9). Additionally, TPP2 knockout adult female mice showed deficient neurogenesis and synaptogenesis, namely, TPP2 depletion leads to impaired proliferation and inefficient differentiation of NSPCs, and downregulation of synaptic protein levels (Figure 11-12). Furthermore, the myelination deficiency of the neurons in the dorsal hippocampal DG and abnormal dendrites in DG IML of TPP2 knockout female mice can be rescued by injection of AAV2/BBB vector harbouring aromatase expression cassette (Figure 12G and H), suggesting that injection of AAV2/BBB can be used to treat TPP2 mutation-induced human **i**ntellectual disability and sterile brain inflammation mimicking MS 6, 7. By contrast, the immunostaining of NeuN and GFAP indicates that the neurogenesis in the hippocampi of TPP2 knockout male mice are normal (Figure 12C). This is most probably due to the fact that both testosterone and dihydrotestosterone enhance survival of new hippocampal neurons in males 184. In fact, estradiol and testosterone have been shown to be effective in females and males, respectively 174, 184-186. This can not only well explain why TPP2 knockout adult female mice are deficient in neurogenesis and synaptogenesis under estrogen deficit despite an increase in testosterones, but also explain why the original Ca^2+^ dyshomeostasis in males is less severe than in females. As Ca^2+^ dyshomeostasis-resultant androgen scaling up can partially rescue its homeostatic state in males, but not in females 187-189. Taken together, TPP2 plays a crucial role in learning and memory of adult female mice by maintaining aromatase and therefore estrogen levels. This non-canonical function of TPP2 is independent of its enzymatic activity, but closely related to its conserved C domain, which keeps the intracellular Ca^2+^ and PC homeostasis through its physical interaction with SERCA1, IP3R1 and VDAC2.

## Materials and Methods

### Animals and antibodies

All experimental knockout and knockin mouse lines and commercial mouse model tools used in this project are on the C57BL/6 background. TPP2 knockout mice were generated using the CRISPR/cas9 genome editing system. For this purpose, two guide RNAs (gRNAs) were designed. While gRNA1 (5´-GCCCCCAATAAATGGTCATTAGG-3´) targets the intron on the 5´ end of Exon 3, gRNA2 (5´-ACATACAAACGTACGCAAGGAGG-3´) targets the intron on the 3´ end of Exon 5, resulting in deletion of a 5342bp long genomic fragment containing Exon 3 to Exon 5 (Figure S2). For preparing conditional TPP2 knockout mice, floxed mice were generated using the CRISPR/cas9 system combined with a donor vector harbouring 6616bp homologous arms and two loxP sites which flank the Exon 4 and Exon 5. In this connection, four gRNAs were designed. Among them, gRNA-A1 (5´-CCTTTTAATATCCATGAACCAGG-3´) and gRNA-B1 (5´-CCAGGACTTATTTTAATGCACTT-3´) target the intron on the 5´ end of Exon4, while gRNA-A2 (5´-ACATACAAACGTACGCAAGGAGG-3´) and gRNA-B2 (5´-TGCACATACAAACGTACGCAAGG-3´) target the intron on the 3´ end of Exon 5 (Figure S3). Mice with specific knockout of TPP2 in excitatory neuron population were obtained by crossing TPP2 floxed mice with CaMK2a-Cre mouse line. Mice with specific knockout of TPP2 in NSCs were obtained by crossing TPP2 floxed mice with Nestin-Cre mouse line. Mice with specific knockout of TPP2 in astrocyte were obtained by crossing TPP2 floxed mice with GFAP-Cre mouse. TRE-TPP2 knockin mice were generated by co-injection of a 18626bp donor vector containing the ´´TRE promoter-Kozak-Mouse TPP2 CDS-3xFLAG-SV40 polyA-anti [CaMKII_alpha_long promoter-tTS-T2A-rtTA-BGH polyA]´´ cassette, Cas9 mRNA and gRNA (GAACACTAGTGCACTTATCCTGG) into fertilized mouse eggs. This cassette was confirmed to be inserted in the Hipp11 (H11) locus between the Eif4enif1 and Drg1 genes on mouse chromosome 11 with southern blot. For southern blot, the primer pair for 5´ probes is 5´-GGCACAATGTTAATCCAGCCTGACTC-3´ and 5´-GTGACCAGTTTGTCCTCCTCCAGTAGA-3´; the primer pair for 3´ probes is 5´-GATGTGAACAAAGCACCCTATGGCTC-3´ and 5´-GTGTCGATCATCCATTAGCCTAGCC-3´ (Figure S4). The number of animals used for each experiment is indicated in the corresponding legend. All antibodies used in this study are listed in Table S1.

### LTP recording and analysis

LTP in the hippocampal CA1 or CA3 region was induced and recorded according the standard protocol. In brief, mice for test were deeply anesthetized with isoflurane and euthanized according to methods approved by the committee on animal care at Xinxiang Medical University, PR China. Mouse brain was removed and then hippocampus was separated from the rest of the brain in chilled artificial cerebrospinal fluid (ACSF). Then the acute transverse hippocampal slices at 400-μm intervals were prepared beginning at approximately 10 mm from the rostral ends using the leica biosystems vibratome (Leica Microsystems, Wetzlar, Germany). Next, the slices were transferred to an interface-type recording chamber where they were perfused at least 1 h for equilibration using oxygenated ACSF at 32°C with a volumetric flow rate of 4 mL/min and their surface exposed to warm and humidified carbogen (95% O2-5% CO2). Field potentials were recorded from SR of CA1 or CA3 using a glass pipette filled with ACSF (resistance was 2-3 MΩ, tip diameter 10 μm). Recordings were band-pass filtered online between 0.5 Hz and 2 kHz using an AxoProbe Amplifier (Digitimer Ltd, Welwyn Garden City, UK) and a NeuroLog system NL106 AC/DC Amplifier (Digitimer Ltd). The data were digitized at a sample rate of 10 kHz using a CED 1401 Plus ADC board (Digitimer Ltd). Electrical interference from the main supply was online eliminated from extracellular recordings using Hum Bug Noise Eliminator (Digitimer Ltd). Postsynaptic potentials (PSPs) were evoked by orthodromic stimulation of the Schaffer collateral/commissural (Sch/com) fibers of the CA3 pyramidal cells to the CA1 pyramidal cells or mossy fiber (MF) of the granule cells of dentate gyrus (DG) to the CA3 pyramidal cells using twisted 50 μm nickel/chromium wires (A-M Systems) or a concentric bipolar microelectrode. Pulses of 0.1 ms duration were delivered every 20s. Pulses were given at varying stimulus intensity to establish a stimulus intensity-response relationship. The downward PSP slope was calculated between 10% and 50% of the maximum amplitude of the PSP. The standard stimulus intensity was set at the intensity that evoked a PSP amplitude 50% of the maximum PSP amplitude. After a stable baseline (15-30 min) was established, LTP was induced by a single HFS train (100 Hz, 1s at standard intensity), after which the PSP slope were recorded for 60 min.

### In vivo electrophysiological recording and analysis

In vivo wideband signals and local field potential (LFP) recordings were carried out as following. The Microwire Array Electrodes (Kedou Brain-Computer Technology, Suzhou, PR China) were implanted in hippocampal CA1 region of anesthetized mouse (1.25 mm lateral to midline, 1.82 mm anterior to posterior and 1.5 mm dorsal to ventral) by help of the Standard Stereotaxic Instrument (RWD Life Science, San Diego, USA). Both wideband signals and LFPs were acquired by OmniPlex Neural Recording Data Acquisition System (Plexon Inc., Dallas, USA) 6 days later after mouse recovery from operation. The recordings were band-pass filtered at 8 kHz and between 200 Hz and 1 kHz using low-pass filters for wideband signals and LFPs, respectively. For analysis of the in vivo electrophysiological recordings, the power spectral density (PSD) and spectrogram of continuous FP were computed using NeuroExplorer (NexTechnologies, Colorado Springs, USA) with 1024 frequency values and 25% window overlap. Before this process, LFP signal values were multiplied by the coefficients of the Hann window. Theta/gamma waves were filtered by band-pass filtering of FP data using NeuroExplorer software with digital filtering of continuous variables function.

### Spike sorting and phase locking analysis

Spikes were sorted with the Offline Sorter (Plexon Inc., Dallas, USA) to classify the electric activity of individual neurons according to the first to third principal components 190. Spike units were excluded using Remove Short ISI Waveform Tools when the absolute refractory period of single unit autocorrelation was < 1 ms. Cross-channel artifacts identified by their time-coincidence across channels were also invalidated. To measure the intensity of theta-gamma phase-amplitude coupling, we used a modulation index (MI) as described 191. To evaluate the wave phase locking, waves were furtherly processed through Hilbert transformation by built-in or custom-built MATLAB (R2016a, MathWorks, USA) scripts. Only neurons with ≥ 50 spikes during the period analysed were used for phase locking estimation. We tested the significance of spike-FP phase locking using circular statistics (CirStat toolbox for Matlab). Rayleigh's test was used to assess the circular distribution of the mean phase angle and to test the non-uniformity of each neuron's spike phase distribution to theta or gamma. The neurons were considered significantly phase-locked if p< 0.05. The mean phase angle was computed as the circular direction of the mean resultant vector. Watson-Williams F-test was performed to compare the mean phase angles of neurons recorded from multi groups. Phase locking of neurons was evaluated by calculating the mean resultant vector (MRL, range 0-1) length of the spike phase angle distribution. The MRL value of 1 indicates exact phase synchrony, whereas a value of 0 indicates no phase synchrony. The non-parametric Kruskal-Wallis test was performed to test MRL value differences between multi groups. p< 0.05 was considered as statistically significant. Moreover, the valley of wave timestamp was identified as a reference event using Find Oscillation function for plotting peri-event raster. The data and MATLAB scripts that support the findings of this study are available from the corresponding authors on request. Phase locking analyses were done with built-in and custom-written routines in Neuroexplorer and MATLAB.

### Two-photon imaging of Ca^2+^ in brain slices

Mouse brain was removed and then the acute transverse brain slices at 200-μm intervals were prepared beginning at approximately 10 mm from the rostral ends using the leica biosystems vibratome (Leica Microsystems, Wetzlar, Germany) in oxygenated mildly modified ACSF (95 mM NaCl, 1.8 mM KCl, 1.2 mM NaH2PO4, 7mM MgSO4, 26 mM NaHCO3, 15 M Glucose, 50.5 mM Sucrose). Next, the slices were transferred to a chamber where they were incubated in continually oxygenated ACSF containing 2 uM Fluo-4 (Beyotime Biotechnology, Shanghai, PR China) at room temperature for 30 min. Two-photon imaging of Ca^2+^ in hippocampal neurons was conducted using Femto3D Atlas (Femtonics, Budapest, Hungary) equipped with a 25 X water immersion objective (MRD77220, numerical aperture: 1.10, working distance: 2 mm) (Nikon, Tokyo, Japan), and a broadly tunable laser which was set at a wavelength of 920 nm and a repletion rate of 1.7 Hz.

### Cell lines and plasmids

293T cell line is from ATCC, Gaithersburg, USA. TPP2-depleted 293T cell clones were established according to standard protocol. In brief, cells were co-transfected with the genome engineering plasmids TPPII CRISPR/Cas9 KO and TPPII HDR (Santa Cruz, Dallas, USA) and were selected for about 2 weeks in complete medium (DMEM medium supplemented with 10% fetal calf serum and antibiotics) containing 1 µg/mL puromycin (Solarbio, Beijing, PR China). Then TPP2-depleted individual clones were isolated, expanded and identified by western blot and antibody staining. Other 293T cell clones which are depleted of ATF6, IRE1, PERK, SYVN1, UCHL1, ATG5, CEPT1, or CCTα, and 293T clones stably expressing TPP2, TPP2 S449A or TPP2 S449T, and CCT with KDEL tag in TPP2 knockout cells were generated by a procedure similar to above. The genome engineering CRIPR/Cas9 plasmids used to delete the expression of these genes and the vectors for ectopic gene expression were constructed by help of Ribobio (Wuhan, PR China) and SinoBiological (Beijing, PR China), respectively. And, the cell clones were selected in complete medium containing 250 µg/mL hygromycin. Plasmids for transient expression of CYP19A1 and its S118A or D mutant in 293T cells were constructed by help of Loyalbio (Zhengzhou, PR China).

### SPLICS to measure MAMs

There are two SPLICS versions efficiently measuring narrow (8-10 nm) and wide (40-50 nm) juxtapositions between ER and mitochondria 192. We used both versions. Plasmids Sac I ML GFP Strand 11 Long (or short) and OMMGFP 1-10 (Addgene, Watertown, USA) were co-transfected using Lipofectamine 3000^TM^ (Thermo Fisher Scientific, Waltham, USA) into TPP2 knockout 293T cells with or without a plasmid for ectopic expression of TPP2, TPP2 S449T or TPP2 S449A fused to an orange fluorescent protein (OFP) reporter. The MAM integrity was detected by confocal microscope Leica TCS SP8 X using 100 X oil objective in green channel (PMT2) with red fluorescence channel (PMT3) against TPP2 or its mutants and blue channel (PMT1) against DAPI (Leica Microsystems, Wetzlar, Germany).

### Transmission electron microscopic imaging

Mice for test were sacrificed and the hippocampi were dissected and stored in 2.5% glutaraldehyde in 0.01 M phosphate buffer. The samples were then further fixed with OsO4 (Ted Pella, Redding USA) in 0.1 M phosphate buffer, dehydrated in ethanol, embedded in SPI-Pon 812 (SPI, West Chester, USA), and polymerized at 65°C. The sections (60-80 nm) were prepared with Leica EM UC7 ultramicrotome (Leica Microsystems, Wetzlar, Germany), and the tissues were fished out onto the 150 meshes cuprum grids with formvar film (Servicebio, Wuhan, PR China). The sections were then sequentially stained with 2% uranyl acetate in ethanol and 2.6% lead citrate. The cuprum grids were observed under transmission electron microscope (Hitachi, Tokyo, Japan).

### Primary hippocampal neuron culture

The protocol for culture of hippocampal neurons was based on a standard procedure with minor modifications 193, 194. In brief, isolated hippocampus was chopped into small pieces in MEM medium (Gibco Life Technologies, Grand Island, USA) and then digested with 5 mL 0.25% trypsin-EDTA (biosharp, Hefei, PR China) at 37°C for 30 min. The digested tissue was further ground in 70 µm cell strainer to obtain the single cell suspension. After centrifugation at 2000 rpm for 2 min, the cell pellet was washed once with phosphate buffered saline (PBS) buffer. Cells were then resuspended in appropriate volume of plating medium (MEM supplemented with 10% horse serum, 100 U/mL penicillin and 100 µg/mL streptomycin) and seeded in 12 well plate containing poly-L-lysine (PLL, Merck, Darmstadt, Germany) coated coverslips. After incubation at 37°C and 5% CO2 for several h, when the cells having attached to the coverslips, the plating medium was replaced with complete neurobasal medium (Neurobasal medium (Gibco) supplemented with 2% B27 (Gibco), 0.5 mM L-glutamine and antibiotics), and the cells were further incubated at 37°C and 5% CO2 for 1 or 2 weeks for Ca^2+^ detection and PLA.

### Intracellular Ca^2+^ determination

To detect the cytoplasmic Ca^2+^ levels in 293T cell clones, cells with or without depletion of TPP2 gene and their counterparts expressing TPP2 mutants were cultured in complete DMEM medium until they were around 60% confluent. After washing 3 times with PBS buffer, cells were incubated with 2 µM Fluo-4 AM (Beyotime Biotechnology, Shanghai, PR China) in PBS buffer at 37°C for 30 min. Then the cells were washed 3 times with PBS buffer and thereafter incubated for another 30 min to make sure that Fluo-4 AM is fully converted to Fluo-4. Finally, the cells were collected and detected by Attune NxT flow cytometer (Thermo Fisher Scientific, Waltham, USA) at Ex 488/ Em 516 nm. For primary hippocampal neurons, neurons cultured on PLL-coated coverslips were stained with Fluo-4 AM as abovementioned and detected by confocal microscope Leica TCS SP8 X (Leica Microsystems, Wetzlar, Germany). To detect Ca^2+^ levels in ER and mitochondria, 293T cell line and modified clones or primary hippocampal neurons were transfected with pCMV G-CEPIA1er, pCAGGS-GCAMP2 and pCMV CEPIA3mt (Addgene, Watertown, USA) using Lipofectamine^TM^ 3000 (Thermo Fisher Scientific, Waltham, USA) according to manufacturer´s instruction. After 48 h 293T cells and neurons were detected by Attune™ NxT Flow Cytometer (Thermo Fisher Scientific, Waltham, USA) or confocal microscopy respectively. To detect Ca^2+^ levels in cytoplasm, 293T cell line and modified clones were stained with Cal-520 (AAT Bioquest, Pleasanton, USA) or transfected with pCAGGS-GCAMP2 (Addgene, Watertown, USA) and then cytosol was fractionated and analysed using plate reader infinite 200 Pro (Tecan, Männedorf, Switzerland) at Ex/Em = 490/525 nm cutoff 515 nm.

### In situ hybridization and semi-quantitative RT-PCR

To detect mRNA levels of CYP19A1 and COMT in brain slice, FISH combined with Tyramide signal amplification (TSA) has been used 195. In brief, after transcardiac perfusion with saline followed by 4% paraformaldehyde (PFA) for 4 h, the brains were dissected and fixed in 4% PFA prepared from DEPC-treated water for about 12 h. After dehydration through increasing gradient alcohol (70%-96%-100%, each exchange 2 X 30 min) and 100% Xylene (3 X 20 min), the tissue was embedded in paraffin and 3 µm-thick slices were prepared with leica biosystems microtome (Leica Microsystems, Wetzlar, Germany). The prepared slices were then sequentially dewaxed with xylene, dehydrated with ethanol, digested with 20 µg/mL proteinase K, blocked with 0.3% H_2_O_2_ in methanol and pre-hydridized in pre-hybridization solution according to standard protocol. The sample slices were then hybridized with 1 µM digoxigenin-labelled CYP19A1 or COMT probe (Servicebio, Wuhan, PR China) in hybridization solution overnight at 42°C. After washing with differently diluted SSC buffers, the slices were blocked at room temperature for 30 min in rabbit serum-containing blocking solution. Then the slices were incubated with anti-digoxigenin-labelled peroxidase (anti-DIG-HRP) (Jackson, Lansing, USA) at 37°C for 40 min. Then, the slices were incubated with Cy3-TSA (Servicebio, Wuhan, PR China) reagent (1/500 in 3% BSA PBST freshly adding 0.003% H_2_O_2_ before use) for 5 min at room temperature after several washing with PBS. Finally, the slices were stained with DAPI and mounted for examination. The images were acquired using the Pannoramic 250 Flash III (3DHISTECH, Budapest, Hungary). The acquired images were viewed with CaseViewer 2.4 (3DHISTECH, Budapest, Hungary) and analysed with ImageJ software. The sequence of CYP19A1 probe is 5´-CGAATTGTTCTCCAAAGGCTCGGGTTGTTGTTAA-DIG-3´; the sequence of COMT probe is 5´-CGTCACCCACGTTCATGGCCCACTCCTTCTCTG-DIG-3´. Both are synthesized by Generalbiol (Chuzhou, PR China). To verify mRNA levels of CYP19A1 and COMT in mouse hippocampus detected by FISH, total RNA was extracted from freshly dissected hippocampus according to manufacturer´s instruction of RNeasy kit (Qiagen, Hilden, Germany), and then was used as template for RT-PCR carried out using the SuperRT one step RT-PCR kit (CWBIO, Taizhou, PR China). The amplified products were separated by agarose electrophoresis and photographied, and are then quantified by ImageJ software. The primer pairs for RT-PCR of CYP19A1 mRNA are: 5´-ATGTTCTTGGAAATGCTGAACCCC-3´ and 5´-GTTAGAGGTGTCCAGCATGATGTG-3´; the primer pairs for RT-PCR of COMT mRNA are: 5´-ATGCTGTTGGCTGCTGTCTCATTGG-3´ and 5´-CTGGGGGATAAGGTCCTGGGATGCC-3´.

### BrdU assay to examine adult neurogenesis

Mice more than 6 months old were given daily 2 mg BrdU (Abcam, Cambridge, UK) in 100 µl of sterile saline via i.p. injection for up to 1 week. After which animals were euthanized and the freshly dissected brains were fixed, dehydrated, embedded and sliced as aforementioned. The prepared slices were then deparaffinized through sequential treatment with xylene (3 X 2 min), 100% ethanol (3 X 2 min) and 70% ethanol (1 X 2 min). After which the endogenous peroxidase activity was quenched in methanol (1 X 2 min) and 3% H_2_O_2_ in methanol (1 X 20 min). The slices were then washed in PBS (3 X 5 min) and digested in 1 mg/mL trypsin PBS for 10 min. The following denaturation of the slices was performed by incubation in 2 M HCL for 30 min at 37°C and neutralization in 0.1 M sodium borate (pH 8.5) for 10 min at room temperature. The following immunostaining was carried out according to standard protocol with three times washing in PBS before each step. In brief, the slices were blocked with horse serum (1/80 in PBS) for 30 min, incubated with anti-BrdU mouse mAb (1/200 in 3% BSA PBST) for 1 h, with HRP-conjugated goat anti-mouse IgG (1/200 in 3% BSA PBST) for 1 h and with Cy3-TSA (1/500 in 3% BSA PBST freshly adding 0.003% H_2_O_2_ before use) for 5 min at room temperature. Finally, the slices were counterstained with DAPI and mounted for microscopic examination and photography. Both antibodies and Cy3-TSA for staining are from Servicebio (Wuhan, PR China).

### Mass spectrometry-based proteomics

For proteomics to find the altered proteins in TPP2 knockout 293T cells, cells were lysed in urea lysis buffer composed of 8M urea, 30 mM HEPES, 1 mM PMSF, 2 mM EDTA. After pulse sonication (2 s ON and 3 s OFF) at 180 W for 3 min, the lysates were centrifuged at 20000 g 4°C for 30 min and the supernatants were collected, which were incubated at 56°C for 1 h after adding 10 mM Dithiothreitol (DTT) and in dark for another hour after adding 55 mM iodoacetamide (IAM). After centrifugation as abovementioned, the supernatants were collected and protein concentrations were determined using Bradford method. Then, about 100 µg protein of each sample or control was added to ultrafiltration spin column (0.5 mL, 10kDa MWCO, PES, Sartorius) and was centrifuged at 14000 g 4°C for 40 min. Then, the column was washed twice by 200 µl 50 mM tetraethylammonium bromide (TEAB). The following trypsin digestion was performed in 50 mM TEAB supplemented with 3.3 µg of trypsin at 37°C for 24 h. The trypsin digestion-produced peptides were then lyophilized and resuspended in 30 µl 0.5 M TEAB, and then were labelled using iTRAQ Reagent-8Plex Multiplex Kit (SCIEX, Framingham, USA) according to manufacturer´s instruction. The labelled samples were further preseparated with Luna SCX 250*4.60mm 100Å column (Phenomenex, Torrance, USA) and desalted with Strata X C18 SPE Column (Phenomenex, Torrance, USA). Desalted peptide mixture was loaded onto an Acclaim PePmap C18 reversed phase column (75μm×2cm, 3 μm, 100Ǻ, thermo scientific) and separated with reversed phase C18 column (75μm×10cm, 5 μm, 300Ǻ, Agela Technologies) mounted on a Dionex ultimate 3000 nano LC system (Spectralab Scientific, Ontario, Canada). Peptides were eluted using a gradient of 5-80% (v/v) acetonitrile in 0.1% formic acid over 45 min at a flow rate of 300 nL/min combined with a Q-Exactive mass spectrometer (Thermo Fisher Scientific, Waltham, USA). The eluates were directly entered Q-Exactive mass spectrometer, setting in positive ion mode and data-dependent manner with full MS scan from 350-2000 m/z, full scan resolution at 70,000, MS/MS scan resolution at 17,500. MS/MS scan with minimum signal threshold 1E+5, isolation width at 2 Da. To evaluate the performance of this mass spectrometry on the iTRAQ labelled samples, two MS/MS acquisition modes, higher collision energy dissociation (HCD) were employed. And to optimize the MS/MS acquisition efficiency of HCD, normalized collision energy (NCE) was systemically examined at 28, steped 20%. The original mass spectrum files were inputted into PD software (Proteome Discoverer 1.4, thermos Fisher Scientific, Waltham, USA) and screened. Then the PD extracted spectrograms were searched with Mascot server (v2.3.01). The filtered spectrograms were then analysed using the ropls R package for Principal Component Analysis (PCA), Partial Least Squares-Discriminant Analysis (PLS-DA), and Orthogonal Partial Least Squares Discrinant Analysis (OPLS-DA). To evaluate the differential proteins ANOVA was used. The screening conditions for differential proteins are p value ≤ 0.05, change ratio ≥ 1.2 or ratio ≤ 0.83.

### Mass spectrometry-based metabolomics

For metabolomics to find the metabolic dyshomeostasis of sex hormones in TPP2 knockout brains and free amino acids in TPP2 knockout 293T cell lines , 100 mg of each sample was suspended in 1 mL of acetonitrile: methanol: ddH2O mixed solution (2:2:1, v/v/v) and vortexed for 30 s. After liquid nitrogen freezing for 5 min and thawing at room temperature, samples were put into a high-throughput tissue grinder and ground at 70 Hz twice, each for 2 min. The samples were then centrifuged at 4°C for 10 min at 13 000 rpm, and the supernatants were transferred to another 2 mL centrifuge tube and concentrated to dry in vacuum. After that, the samples were dissolved with 300 μL of 2-chlorobenzalanine solution (4 ppm) prepared with acetonitrile: 0.1% formic acid (1:9 v/v), and the supernatants were filtered through 0.22 µm membrane to obtain the prepared samples for LC-MS (20 µL from each sample was aliquoted for the quality control). Chromatographic separation was accomplished in Thermo Ultimate 3000 system equipped with an ACQUITY UPLC® HSS T3 column (150×2.1 mm, 1.8 µm, Waters) maintained at 40°C, with the autosampler maintained at 8°C. After column equilibration, 2μL of each sample was injected and the analytes were eluted with an increasing linear gradient (2-98% v/v) of acetonitrile in 0.1% formic acid over 20 min at a flow rate of 0.25 mL/min 196, 197. The ESI-MS experiments were executed on the Thermo Q Exactive mass spectrometer with the spray voltage of 3.8 kV and -2.5 kV in positive and negative modes, respectively. Sheath gas and auxiliary gas were set at 30 and 10 arbitrary units, respectively. The capillary temperature was 325°C. The analyzer scanned over a mass range of 81-1000 m/z for full scan at a mass resolution of 70,000. Data dependent acquisition (DDA) MS/MS experiments were performed with HCD scan. The normalized collision energy was 30 eV. Dynamic exclusion was implemented to remove some unnecessary information in MS/MS spectra. For data analysis, the obtained raw data was firstly converted into mzXML format through ProteoWizard software (v3.0.8789) and then XCMS R package (v3.3.2) was used for peak identification, peak filtering, and peak alignment. The main parameters are bw=2, ppm=15, peak width = c(5,30), mzwid = 0.015, mzdiff = 0.01, and method = centWave. The ropls R package was used for multivariate statistical analysis (PCA, PLS-DA, and OPLS-DA) as abovementioned. To screen for differential metabolites ANOVA was used with the conditions as follows: p value ≤ 0.05, change ratio ≥ 1.5 or ratio ≤ 0.667.

### Mass spectrometry-based lipidomics

For lipidomics to find the lipid dyshomeostasis in TPP2 knockout mouse brains, 100 mg of each sample was transferred into 750 µl of precooled chloroform/methanol (2:1) solution in 2 mL centrifuge tube containing several steel balls. After grinding at 60 Hz for 1 min in a high flux organization grinding apparatus, the samples were incubated on ice for 40 min. Then, each sample was supplemented with 190 µl ddH2O and incubated on ice for 10 more min after vortex for 30 s. After centrifugation at 12000 rpm for 5 min at room temperature, 300 μL lower layer fluid was transferred into a new centrifuge tube and 500 µl precooled chloroform/methanol (2:1) solution was supplemented and vortexed for 30 s. The samples were centrifuged once again as above and 400 µl lower layer fluid for each sample was transferred into a new centrifuge tube and was concentrated to dry in vacuum. The sample was finally dissolved with 200 μL isopropanol and the supernatant was filtered through 0.22 µm membrane to obtain the prepared sample 198. Chromatographic separation was accomplished in an Thermo Ultimate 3000 system equipped with an ACQUITY UPLC BEH C18 column (100 × 2.1 mm, 1.7 µm, Waters) which was maintained at 50°C, with the autosampler maintained at 8°C. After column equilibration, 2μL of each sample was injected and the analytes were eluted with an increasing linear gradient (50-100%) of acetonitrile/isopropanol (5/2 v/v) prepared in 0.1% formic acid and 10 mM ammonium formate at a flow rate of 0.25 mL/min over 40 min 199. The ESI-MS experiments were executed on the Thermo Q Exactive Focus mass spectrometer with the spray voltage of 3.5 kV and -2.5 kV in positive and negative modes, respectively. Sheath gas and auxiliary gas were set at 30 and 10 arbitrary units, respectively. The capillary temperature was 325°C. The Orbitrap analyzer scanned over a mass range of m/z 150-2 000 for full scan at a mass resolution of 35 000. DDA MS/MS experiments were performed with HCD scan. The normalized collision energy was 30 eV. Dynamic exclusion was implemented to remove some unnecessary information in MS/MS spectra. For data analysis, the obtained raw data were firstly preprocessed through LipidSearch software (v4.0) to get the data matrix composed of mass to charge ratio (m/z), retention time (rt) and peak response value (intensity), which was further used for peak filtering and alignment. The main parameters are R.T. Tolerance=0.25, m Score threshold=5. The ropls R package was used for multivariate statistical analysis (PCA, PLS-DA, and OPLS-DA) as abovementioned. To screen for differential lipids ANOVA was used with the conditions as follows: p value ≤ 0.05, change ratio ≥ 1.5 or ratio ≤ 0.667.

### LD staining of 293T cell clones and primary hippocampal cells

To analyze LD content of different 293T cell clones and primary hippocampal cells with or without gene modification, 60% confluent 293T cell clones or freshly dissociated hippocampal cells were fixed in 4% PFA at room temperature for 15 min (10^6^ cells/mL). After washing twice with PBS and permeabilization in 0.1% Triton X-100 PBS for 2 min, cells were incubated with 1 X lipidSpot^TM^ LD Stains (Biotium, Fremont, USA) diluted with PBS at room temperature for 10 min in darkness. Then, the samples were detected directly by Attune™ NxT Flow Cytometer (Thermo Fisher Scientific, Waltham, USA) at Ex 488 /Em 585 nm.

### PLA to detect PPI

PPIs in 293T cells and primary hippocampal neurons with or without gene modification were detected by Duolink^R^ In Situ Fluorescence PLA kits (Olink Bioscience, Uppsala, Sweden). The experimental procedure was according to manufacturer´s instruction with mild modification. In general, before starting PLA, unless otherwise specified, 293T cells or neurons were not pretreated with any chemical reagents, except for studying on PPIs between ER stress sensors (ATF6, IRE1 and PERK) and GRP78, TPP2 knockout 293T cells or neurons were preincubated with 5 µM Thapsigargin (TG) or 1 µM (-)-Xestospongin C (XeC) at 37°C for 5 h, or 3 µM Phosphatidylcholone (PC) at 37°C for 48 h. The pretreated or untreated samples were sequentially fixed with 4% PFA at room temperature for 15 min, permeabilized in 0.1% Triton X-100 PBS at room temperature for 5 min after washing twice (5 min X 2), and blocked in blocking solutiuon provided by the kit at 37°C for 30 min. The following incubation of primary antibodies and PLA probes, ligation and amplification, and mounting the slides for imaging were as described in product manual.

### IF staining and STED imaging

Cells were permeabilized with phosphate-buffered saline (PBS) containing 0.1% Triton X-100 and 1% donkey serum for 20 min, and blocked with PBS containing 0.1% Triton X-100 and 10% donkey serum or 3% BSA for 1 h. Afterwards, the cells were incubated with first antibody at room temperature for 1 h in a wet box, and after washing thrice incubated with appropriate fluorescent secondary antibodies at room temperature in dark for 1 h. The following sealing and mounting of the slides for imaging are as described in standard protocol. The images were captured using confocal microscope Leica TCS SP8 X using 488 nm and 647 nm excitation lasers coupled with STED 592 nm and 660 nm depletion lasers (Leica Microsystems, Wetzlar, Germany). All images acquired with XY/Z_stack_ model were deconvolved using Huygens software (Scientific Volume Imaging, Hilversum, Holland) and analysed using LAS X software (Leica Microsystems, Wetzlar, Germany).

### IHC staining

Mice for test were anesthesized and transcardially perfused with saline followed with 4% PFA for 4 h. The brains were then dissected and stored in 4% PFA at 4°C overnight. Then, paraffin sections or frozen sections were prepared as follows. For preparation of paraffin sections, the brains were dehydrated in increasing gradients of alcohol (70%-96%-100%, each exchange 2 X 30 min) and 100% Xylene (3 X 20 min). Next, the brains were put in freshly melted paraffin wax (58°C) for embedding and allowed to cool down overnight under room temperature, and 3 µm-thick slices were prepared with leica biosystems microtome (Leica Microsystems, Wetzlar, Germany). For preparation of frozen sections, the 4% PFA fixed brain tissues were cryoprotected in 30% sucrose until they sink. Then samples were embedded in OCT in cryomolds and snap frozen in prechilled isopentane or on dry ice. Frozen tissues can be stored in a -80 freezer and/or 10 µm-thick slices were prepared with leica biosystems cryostat (Leica Microsystems, Wetzlar, Germany). Before incubation with the first antibody, paraffin slices were deparaffinized and rehydrated by incubations with graded xylene and ethanol in water (Xylene 2 X 5 min, 1:1 Xylene:100% ethanol 5min, 100% ethanol 2 X 5min, 95% ethanol 5min, 70% ethanol 5 min, 50% ethanol 5 min, Deionized water 2 X 5 min). Then, the following steps were used for both paraffin and frozen sections. Slices were sequentially baked in a 37°C oven for 10-20 min, fixed in 4% PFA for 30 min, incubated with 1mM EDTA antigen repair buffer (pH 8.0) for appropriate time, permeabilized with phosphate-buffered saline (PBS) containing 0.4% Triton X-100 and 1% donkey serum for 20 min, and blocked with PBS containing 0.1% Triton X-100 and 10% donkey serum or 3% BSA for 1 h. Afterwards, the tissue slices were incubated with first antibody overnight at 4 ° C in a wet box, and after washing thrice incubated with appropriate fluorescent secondary antibodies at room temperature in dark for 1 h. The following sealing and mounting of the slides for imaging are as described in standard protocol. The images were captured using Pannoramic 250 Flash III or Pannoramic Midi and viewed with CaseViewer 2.4 (3DHISTECH, Budapest, Hungary) and analysed with ImageJ software.

### Co-IP

5x10^6^ cells or 10 mg homogenized brain tissue were suspended in 600 μL of lysis buffer (50 mM Tris-HCl pH 7.4, 1% Triton X-100, 1% n-Octyl β-D-glucopyranoside, 0.1% SDS, 1mM EDTA pH 7.0, 150 mM NaCl) supplemented with 1 X protease inhibitor cocktail (MERCK, Darmstadt, Germany) and incubated for 1 h on ice. Then, the lysates were centrifuged at 12,000 rpm for 10 min at 4°C to get rid of cellular nuclei and debris. The supernatant was transferred to fresh tube and protein concentration was measured using Pierce BCA Protein Assay Kit (Thermo Fisher Scientific, Waltham, USA). According to acquired protein concentration, appropriate amount of protein G agarose beads (MERCK, Darmstadt, Germany) resuspended in lysis buffer was added to preclear the non-specific bindings at 4°C for 30 min in rotating state. Meanwhile, target protein antibody conjugated beads or isotype antibody conjugated control beads were prepared by routine incubation and centrifugation protocol. Finally, the precleared supernatants and target protein antibody conjugated beads or isotype antibody conjugated control beads were combined and incubated for 2 h at 4°C in mild agitation state to pull down interested proteins and its interaction partners. After thrice centrifugation and washing with lysis buffer, the beads were resuspended in 100 μL lysis buffer and for each immunoblot 20 μL was used.

### Immunoblot

Cells or homogenized brain tissue were suspended in appropriate volume of RIPA lysis buffer (50 mM Tris-HCl pH 7.4, 1% Triton X-100, 1% sodium deoxycholate, 0.1% SDS, 1mM EDTA pH 7.0, 150 mM NaCl) supplemented with 1 X protease inhibitor cocktail (MERCK, Darmstadt, Germany) and with or without 1 X phosphatase inhibitor cocktail (MERCK, Darmstadt, Germany) and incubated for 1 h on ice. Then, the lysates were centrifuged at 12,000 rpm for 10 min at 4°C to get rid of cellular debris. The supernatant was transferred to fresh tube and protein concentration was measured using Pierce BCA Protein Assay Kit (Thermo Fisher Scientific, Waltham, USA). Then, 30 μg cell or tissue lysate or 20μL immunoprecipitate for each sample was mixed with equal volume of 2 X loading buffer (4% SDS, 10% 2-Mercaptoethanol, 20% Glycerol, 0.004% Bromophenol blue, 0.125 M Tris-HCl, pH 6.8) and heated at 95°C for 10 min. The following Sodium Dodecyl Sulfate-Poly Acrylamide Gel Electrophoresis (SDS-PAGE) and Western blotting were according standard protocol. The blotted polyvinylidene fluoride (PVDF) or nitrocellulose (NC) membrane was blocked with 3% Bovine serum albumin (BSA) at room temperature (RT) for 1 h. Then, first antibody incubation (30 min to 1 h), Phosphate-buffered saline, 0.1% Tween 20 (PBST) or Tris-buffered saline, 0.1% Tween 20 (TBST) washing (3 X 10 min), and Horseradish peroxidase (HRP)-labelled secondary antibody incubation (1 h) were sequentially carried out at RT. At last, the membrane were incubated with enhanced chemiluminescence (ECL) chromogenic substrate solution for 1 min at RT, and the signals were recorded with film or charge-coupled device (CCD) cameras according to manufacturer's instruction and analysed with ImageJ software.

### TUNEL assay of mouse brain slices

TUNEL assay was carried out using the TMR (red) tunel cell apoptosis detection kit (Servicebio, Wuhan, PR China) according to product manual. In brief, paraffin brain sections of tested mice were prepared, deparaffinized, and rehydrated as described in IHC staining. The assay on the prepared sections was carried out according to manufacturer's instruction. In brief, the rehydrated sections were firstly incubated with protease K working solution in PBS at 37°C for 20min. After washing in PBS 3 X 5 min, the sections were permeabilized in PBS containing 0.4% Triton X-100 at RT for 20 min. Then, the sections were washed in PBS 3 X 5 min and equilibrated in equilibration buffer at RT for 10 min. Finally, appropriate volume of tunel reaction solution (TdT enzyme : TMR-5-dUTP labeling mix : Equilibration buffer: 1 : 5 : 50) was applied and the reaction was kept at 37°C for 1 h in a wet box. The following sealing and mounting of the slides for imaging are as described in standard protocol. TMR (Tetramethyl-Rhodamine) glows red by Ex 555/Em 580 nm. The images were captured using Pannoramic 250 Flash III and viewed with CaseViewer 2.4 (3DHISTECH, Budapest, Hungary) and analysed with ImageJ software.

### Senescence assay of mouse brain slices

Senescence assay was conducted using the β-Galactosidase staining kit (Servicebio, Wuhan, PR China) according to product manual. In brief, mice for test were anesthesized and transcardially perfused with saline for 4 h. The brains were then dissected and cryoprotected in 30% sucrose until they sink. Then samples were embedded in OCT in cryomolds and snap frozen on dry ice. 10 µm-thick slices were prepared with leica biosystems cryostat (Leica Microsystems, Wetzlar, Germany). The staining was conducted as described in product manual. In brief, frozen sections were firstly reheated to RT and rinsed in PBS. Then, the tissues were incubated in fixative solution at RT for 20 min. After washing (3 X 5 min) in PBS, the tissues were incubated in appropriate volume of freshly prepared β- Galactosidase staining working solution at 37°C in a wet box. Color development was observed under a microscope every 2 h until the next day. Afterwards, the staining solution was removed and the slices were soaked in PBS twice, followed by soaking in ddH2O twice. Then, the slices were incubated with nuclear fast red (NFR) staining solution at RT for 3 min, washed thrice with ddH2O. The slices were then dehydrated in anhydrous ethanol (2 X 5 min), made transparent with xylene for 5 min, and mounted with neutral balata. The images were captured using Pannoramic 250 Flash III and viewed with CaseViewer 2.4 (3DHISTECH, Budapest, Hungary).

### LFB staining of mouse brain slices

Paraffin brain sections of tested mice were prepared, deparaffinized, and rehydrated as described in IHC staining. The slices were then incubated in LFB solution (1% LFB in 95% ethanol and 0.05% acetic acid) at RT overnight. After rinsing in ddH2O, the slices were differentiated in 0.05% lithium carbonate solution up to 20 s, then in 70% ethanol until gray matter and white matter are clearly differentiated. The slices were continually incubated in 0.25% cresyl echt violet acetate in ddH2O at RT for 5 min. At last, the slices were dehydrated in anhydrous ethanol (2 X 5 min), made transparent with xylene for 5 min, and mounted with neutral balata. The images were captured using Pannoramic 250 Flash III and viewed with CaseViewer 2.4 (3DHISTECH, Budapest, Hungary).

### Cellular fractionation

Cellular fractionation was performed using the ER enrichment kit (Novus Biologicals, Centennial, USA) according to product manual. In brief, 0.5 g cells were harvested and washed in PBS, then resuspended in 2 mL 1 X isosmotic homogenization buffer supplemented with protease inhibitor cocktail and homogenized thoroughly. The homogenate was centrifuged at 1000 g for 10 min at 4°C to get rid of nuclear, mitochondria, and cell debris. The supernatant was transferred to a new tube and centrifuged at 12,000 g for 15 min at 4°C. Then, the supernatant was transferred to a microcentrifuge tube and centrifuged at 90,000 g (Beckman Avanti J30I centrifuge with JS24 rotor) for 60 min at 4°C. The supernatant was kept as cytosol and the pellet (which contains the total ER fraction) was suspended in 1 X suspension buffer supplemented with protease inhibitor cocktail. Prepared cytosol and ER were kept at -80°C for further immunoblot.

### TPP2 enzymatic activity assay

Cells cultured to 70-80% confluent were harvested and washed thrice in PBS. Then, cells were resuspended in 4 volumes of freshly prepared homogenization buffer (50 mM Tris-HCl, pH7.5, 250 mM Sucrose, 5 mM MgCl2, 0.5 mM EDTA, supplemented with 2 mM ATP, 1 mM DTT and 0.025% Digitonin before use) and incubated on ice for 5 min to allow permeabilization by digitonin. Cytosol was squeezed out by centrifugation at 20,000 g for 15 min at 4°C and transferred to a new tube. Protein concentration was measured using Pierce BCA Protein Assay Kit (Thermo Fisher Scientific, Waltham, USA). In between, 200 μM (2 X) AAF-AMC (MERCK, Darmstadt, Germany) substrate solution with or without 100 μM (2 X) AAF-CMK (MCE, Monmouth Junction, USA) was prepared by diluting 100 X stock solutions (100 mM AAF-AMC in anhydrous ethanol, 50 mM AAF-CMK in ddH2O) in the assay buffer (50 mM Tris-HCl, pH7.5, 40 mM KCl, 5 mM MgCl2, supplemented with 0.5 mM ATP, 1 mM DTT and 0.5 mg/mL BSA before use). Then, the 2 X substrate solution was transferred into a well of 96 well plate and incubated at 37°C for 5 min. At last, cytosol equal to 5 μg proteins was added to the well and assay buffer was supplemented to 200µL total volume. The fluorescent signal was recorded with Infinite 200 PRO (Tecan, Männedorf, Switzerland) at Ex 380/Em 460 nm.

### MWM test

MWM test has proven to be a robust and reliable test for spatial learning and memory potentials that are strongly correlated with hippocampal synaptic plasticity and NMDA receptor function 200. In this study, the MWM test was carried out according to a nature protocol report 200 with mild modifications. In brief, a 120 cm diameter circular pool with a 10 cm^2^ platform in one quadrant was filled with tap water until the platform was submerged 1 cm beneath the water surface. The platform was then camouflaged by placing opacifying titanium dioxide in the water and the water temperature was maintained at 23°C. During the 5 days training, each mouse received 4 trials per day. The sequences of trial start position were designed such that the escape platform was to the right or left of an animal during 4 training trials and one trial occured from each of the four start positions each day. During the training trials, if a mouse failed to find the platform within 70 s, it was gently guided to the platform and allowed to stay on it for 30 s. The time to find the platform was recorded as the escape latency. On day 6, the probe trials were performed at the absence of platform to record the time, speed, the swimming distance, and the number of crossing the former platform location within 70 s with vedio recorder connected to the EthoVision XT software (Noldus, Leesburg, USA) to evaluate the spatial memory.

### Preparation and application of virus particles for aromatase rescue experiment

The mouse cyp19a1 cDNA was amplified by PCR using C57BL/6 mouse genomic DNA as template, phanta super-fidelity DNA polymerase (Vazyme, Nanjing, PR China), and the following forward and reverse primers: 5'-CTGAGAGCGCAGTCGAGAAGGTACCGCCACCATGTTCTTGGAAATG-3' and 5'-TCCTCTGCCCTCACTAGTGCTAGCTTTGTCGTCATCATCCTTATAGTCC-3'. The acquired cDNA after sequencing validation was cloned into the adeno-associated virus type 2 (AAV2) derived vector pHBAAV-hsyn-MCS-T2A-ZsGreen (HANBIO, Shanghai, PR China) between KpnI and BamHI cutting sites to get the final plasmid pHBAAV2/BBB-m-cyp19a1-3 X Flag-T2A-ZsGreen. After high purity endotoxin free extraction, the final plasmid was co-transfected with pAAV-RC and pHelper into 293T cells using Lipofiter^TM^ transfection reagent (HANBIO, Shanghai, PR China). Cell precipitates were harvested 72 h after transfection and high titer virus preservation solution was obtained after column purification, and various indicators of virus were determined according to strict quality standards (HANBIO, Shanghai, PR China). Then, 100 μL (1.4 X 10 ^11^ vector genome (vg)) recombinant virus or control virus particles per 9 month-old mouse were delivered through injection into the tail vein. One month later, the learning and memory abilities of the experimental mice were measured using the MWM test. Expression of the recombinant aromatase was validated by whole brain imaging using IVIS Lumina III in vivo imaging system (PerkinElmer, Watham, USA) and by immunoplat.

### Statistical analysis

All the experimental results were analysed using GraphPad Prism 8.0.1 (La Jolla, USA). Data were presented, unless otherwise noted, as mean ± SD from at least 6 independent experiments except WB results which were from 3 independent experiments. In general, to compare two groups of data either two-tailed unpaired student's t-test or Mann-Whitney U-test was implemented for parametric data or nonparametric data, respectively. To compare more than two groups of data, either one-way ANOVA followed by Bonferroni post hoc test or Kruskal-Wallis test was implemented for parametric data or nonparametric data, respectively. To compare the escape latency and LTP of different groups of mice (slices) two-way repeated measures ANOVA followed by Tukey's test was used. The normal distribution of the variances were assessed using the Kolmogorov-Smirnov Goodness of Fit Test.

## Supplementary Material

Supplementary figures and table.
