# Supplementary material for: Tripeptidyl peptidase II coordinates the homeostasis of calcium and lipids in the central nervous system and its depletion causes presenile dementia in female mice through calcium/lipid dyshomeostasis-induced autophagic degradation of CYP19A1
Source: Theranostics. 2024 Jan 27;14(4):1390–429. doi: 10.7150/thno.92571 (PMC10879859; doi:10.7150/thno.92571)
Supplement: Supplementary file 1 — Supplementary figures and table. [file thnov14p1390s1.pdf]

Table S1 Antibodies used in this study

Antibodys	Source	Identifier
Acetyl-p53 (Lys319) Ab	Affinity	AF1024
ACLY Monoclonal antibody	Proteintech	67166-1-IG
AHR Polyclonal Antibody	Proteintech	17840-1-AP
AIF1/IBA1 Rabbit mAb	Abclonal	A19776
AIP/ARA9 Polyclonal Antibody	Proteintech	18176-1-AP
ALDH1L1 Rabbit mAb	Abclonal	A7707
Anti -CD31 Rabbit pAb	Servicebio	GB11063-2
Anti -Ki67 Rabbit pAb	Servicebio	GB111141
Anti-beta III Tubulin Rabbit pAb	Servicebio	GB11139
Anti-CD90/Thy1 Rabbit pAb	Servicebio	GB11182-1
Anti-DERL1/Derlin-1 antibody	Abcam	AB176732
Anti-HOMER3 Rabbit pAb	Servicebio	GB111825
Anti-IRE1 (phospho S724)	Abcam	AB48187
Anti-Myelin Basic Protein antibody[EPR21188]	Abcam	AB218011
Anti-NR2F2 Antibody	Boster	PB0770
Anti-SEL1L antibody	Abcam	AB78298
Anti-Synaptophysin Rabbit pAb	Servicebio	GB11553
Anti-TRAM1	Abcam	AB96106
Aromatase (D5Q2Y) Rabbit mAb	Cell Signaling	14528
Aromatase Mouse Polyclonal antibody	Bioss	bs-0114M
ATF4 Antibody	Affinity	AF5416
ATF6 monoclonal antibody	Proteintech	66563-1-IG
ATF6 Polyclonal antibody	Proteintech	24169-1-AP
ATP2A1 Polyclonal antibody	Proteintech	22361-1-AP
ATP2A1/SERCA1 (L24) Antibody	Cell Signaling	4219
BAD Antibody	Affinity	AF7927
Bak Antibody	Cell Signaling	3814
BAP31 Polyclonal antibody	Proteintech	11200-1-AP
Bax Antibody	Cell Signaling	2772
BRD7 Polyclonal Antibody	Proteintech	51009-2-AP
Calreticulin Antibody	Affinity	BF0641

CAMK2 Polyclonal Antibody	Proteintech	13730-1-AP
CaMK2 alpha/delta (pT286) Antibody	Abmart	PA3812S
Caspase 3 antibody	Affinity	AF6311
CCTα (D18B6) Rabbit mAb	Cell Signaling	6931
CD11B Rabbit pAb	Abclonal	A1581-50
Choline Kinase Alpha Polyclonal antibody	Proteintech	13520-1-AP
Choline Kinase α (D5X9W) Rabbit mAb	Cell Signaling	13422
CHPT1 Polyclonal Antibody	Invitrogen	PA5-23695
Cleaved-Caspase 3 (Asp175), p17 Antibody	Affinity	AF7022
COMT Rabbit Polyclonal Antibody	Proteintech	14754-1-AP
CYP11A1 Rabbit Polyclonal Antibody	Proteintech	13363-1-AP
CYP17A1 Rabbit Polyclonal Antibody	Proteintech	14447-1-AP
CYP1A1 Polyclonal antibody	Proteintech	13241-1-AP
CYP1A2-Specific Polyclonal antibody	Proteintech	19936-1-AP
CYP1B1 Polyclonal antibody	Proteintech	18505-1-AP
CYP3A4 Polyclonal antibody	Proteintech	18227-1-AP
DCX Polyclonal antibody	Proteintech	13925-1-AP
DGAT1 Polyclonal antibody	Proteintech	11561-1-AP
ELOVL4 Polyclonal Antibody	Proteintech	55023-1-AP
EOMES/TBR2 Polyclonal Antibody	Proteintech	28316-1-AP
EphA4 Polyclonal antibody	Proteintech	21875-1-AP
ER Polyclonal antibody	Proteintech	21244-1-AP
Ero1-Lα Antibody	Cell Signaling	3264
ESR2 Polyclonal antibody	Proteintech	14007-1-AP
ESRRG Rabbit Polyclonal Antibody	Proteintech	14017-1-AP
FAM134B Polyclonal antibody	Proteintech	21537-1-AP
FASN Polyclonal Antibody	Proteintech	10624-2-AP
Flag-Tag Mouse Monoclonal Antibody	Abways	AB0008
GFAP Rabbit Polyclonal Antibody	Proteintech	16825-1-AP
GFP tag polyclonal antibody	Proteintech	50430-2-AP
GRP78 Antibody	Affinity	AF5366
GRP78/BIP Monoclonal antibody	Proteintech	66574-1-IG
GRP94 Rabbit Polyclonal Antibody	Proteintech	14700-1-AP
HES1 Rabbit mAb	Abclonal	A0925

HMGCR Rabbit mAb	Abclonal	A19063
HSD17B2 Rabbit Polyclonal Antibody	Proteintech	10978-1-AP
HSD17B4 Rabbit Polyclonal Antibody	Proteintech	15116-1-AP
HSD3B1 Rabbit mAb	Abclonal	A19266
HSD3B2 Rabbit pAb	Abclonal	A1823
IBA1 Polyclonal antibody	Proteintech	10904-1-AP
iPLA2 Polyclonal antibody	Proteintech	22030-1-AP
IRE1; ERN1 Polyclonal antibody	Proteintech	27528-1-AP
ITPR1-specific Polyclonal antibody	Proteintech	19962-1-AP
LAMP1 Polyclonal antibody	Proteintech	21997-1-AP
LHX2 antibody	GeneTex	GTX129241
LPCAT1 Polyclonal antibody	Proteintech	16112-1-AP
MAP2 Rabbit Polyclonal Antibody	Proteintech	17490-1-AP
MOG Rabbit Polyclonal Antibody	Proteintech	12690-1-AP
MOGAT2 Rabbit Polyclonal Antibody	Proteintech	19514-1-AP
Myelin Basic Protein Rabbit Polyclonal Antibody	Proteintech	10458-1-AP
Nestin Monoclonal antibody	Proteintech	66259-1-Ig
NeuN Rabbit Polyclonal Antibody	Proteintech	26975-1-AP
NEUROD1 Polyclonal antibody	Proteintech	12081-1-AP
Occludin Polyclonal antibody	Proteintech	27260-1-AP
OLIG2 Monoclonal Antibody	Proteintech	66513-1-IG
OS9 Antibody	Affinity	DF8249
OSBP Rabbit Polyclonal Antibody	Proteintech	11096-1-AP
p53 (1C12) Mouse mAb	Cell Signaling	2524
P62/SQSTMQ rabbit polyclonal antibody	Proteintech	18420-1-AP
PCYT1A Antibody (monoclonal) (M02)	Abcepta	AT3243a
PDI (C81H6) Rabbit mAb	Cell Signaling	3501
PEMT Antibody	Novus	NBP1-59580
Perilipin 5 Polyclonal antibody	Proteintech	26951-1-AP
PERK Monoclonal Antibody (5G5)	Thermo Fisher	MA5-15705
PERK/EIF2AK3 Rabbit Polyclonal Antibody	Proteintech	24390-1-AP
Phospholamban Monoclonal Antibody (2D12)	Thermo Fisher	MA3-922
PLB1 Polyclonal antibody	Proteintech	26835-1-AP
PLIN4 Polyclonal antibody	Proteintech	55404-1-AP

PNPLA6 Polyclonal antibody	Proteintech	14261-1-AP
PPP3CA Polyclonal Antibody	Proteintech	13422-1-AP
PROX1 Polyclonal antibody	Proteintech	11067-2-AP
PSD95-Specific,DLG4 Polyclonal antibody	Proteintech	20665-1-AP
PSMC4 Polyclonal Antibody	Proteintech	11389-1-AP
PUMA Rabbit Polyclonal Antibody	Proteintech	55120-1-AP
RAB7A Polyclonal antibody	Proteintech	55469-1-AP
Rabbit Anti-CEPT1 Polyclonal antibody	Bioss	bs-12284R
Rabbit monoclonal [EP7459] to SYVN1/HDR1	Abcam	AB170901
Rabbit monoclonal [EPR22023] to Nestin	Abcam	AB221660
Rabbit Polyclonal to IRE1	Abcam	AB48187
SERCA2,ATP2A2 Monoclonal antibody	Proteintech	67248-1-IG
SIGMAR1 Polyclonal antibody	Proteintech	15168-1-AP
SLC25A1 Polyclonal antibody	Proteintech	15235-1-AP
TBR1 Polyclonal Antibody	Proteintech	20932-1-AP
TIP47 Rabbit Polyclonal Antibody	Proteintech	10694-1-AP
TPP2 Monoclonal antibody	Proteintech	66017-1-IG
TPP2 Polyclonal antibody	Proteintech	14120-1-AP
UCHL1 (D3T2E) XP® Rabbit mAb	Cell Signaling	13179
UGGT1 Antibody	Affinity	DF8205
VAPB Monoclonal antibody	Proteintech	66191-1-IG
VAPB Rabbit Polyclonal Antibody	Proteintech	14477-1-AP
VCP Rabbit Polyclonal Antibody	Proteintech	10736-1-AP
VDAC (D73D12) Rabbit mAb	Cell Signaling	4661
VDAC2 Mouse Monoclonal Antibody	Proteintech	66388-1-IG
XBP-1s (E9V3E) Rabbit mAb	Cell Signaling	40435
β-Actin Rabbit mAb (High Dilution)	Abclonal	AC026

Figure S1

A Co-IP showing that TPP2 mediates SERCA1/PLN and prevents IP3R1/CALR interactions when cytosolic calcium is decreased by BAPTA treatment (25 μM BAPTA X 30 min)

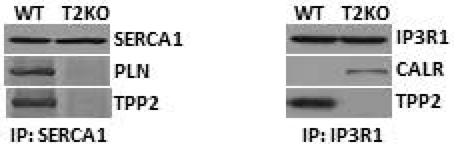

B PLA showing increased PPI between CCTα and CPT1 in TPP2 KO 293T cells as compared with WT cells

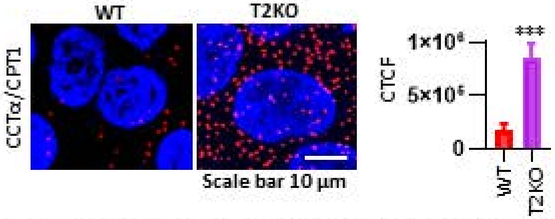

C Dot plot displaying the top 10 enriched GO BPs of upregulated proteins in TPP2 knockout 293T cells.

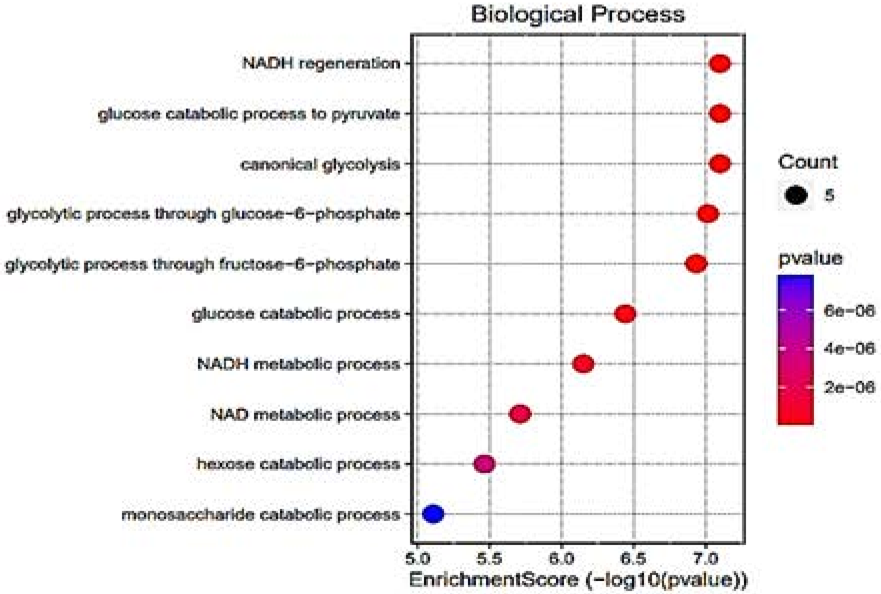

D TPP2 gradually decreases with age in mouse CNS

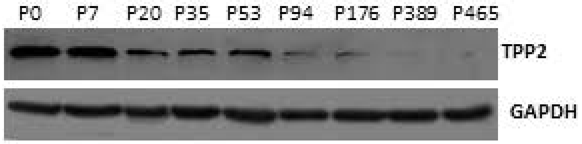

Figure S2

Preparation of non-conditional tpp2 KO mice with CRISPR/Cas9 tool (genomic DNA sequencing shows a 5370bp fragment containing exon2 is successfully deleted)

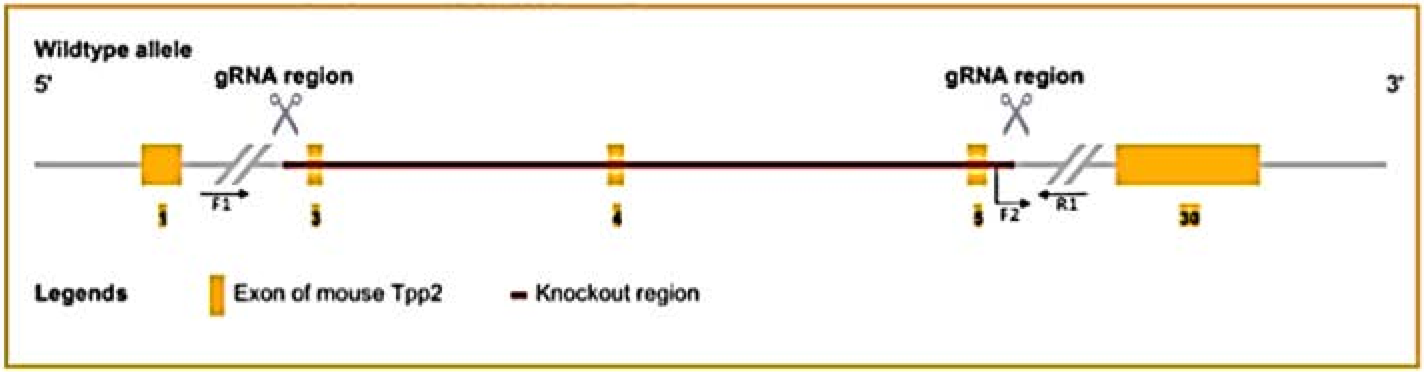

Positive animals

Mouse ID: 10, 12, 15 (Deleted 5370 bp)

TAGAGAGACCACATACATGCCCCCTGCCCCCAATAAATGG--del 5370 bp--AGGGTTGGTCTTTACGTAACATGCATTTCATCTGTGACCT

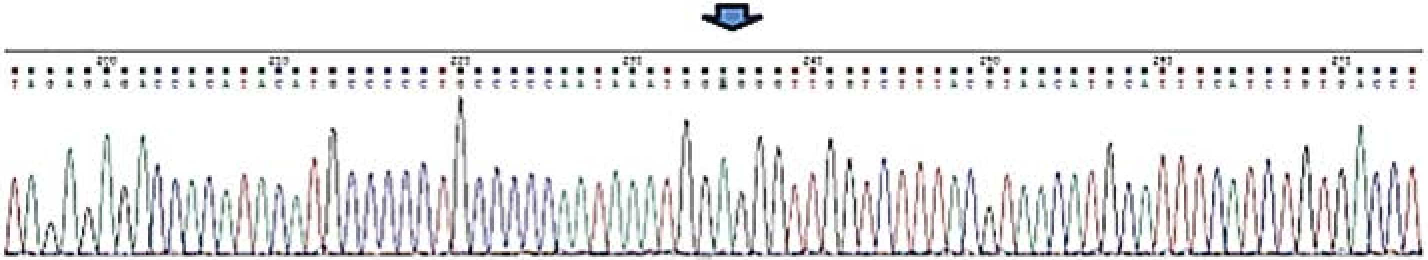

Figure S3

Preparation of conditional tpp2 KO mice with CRISPR/Cas9 tool

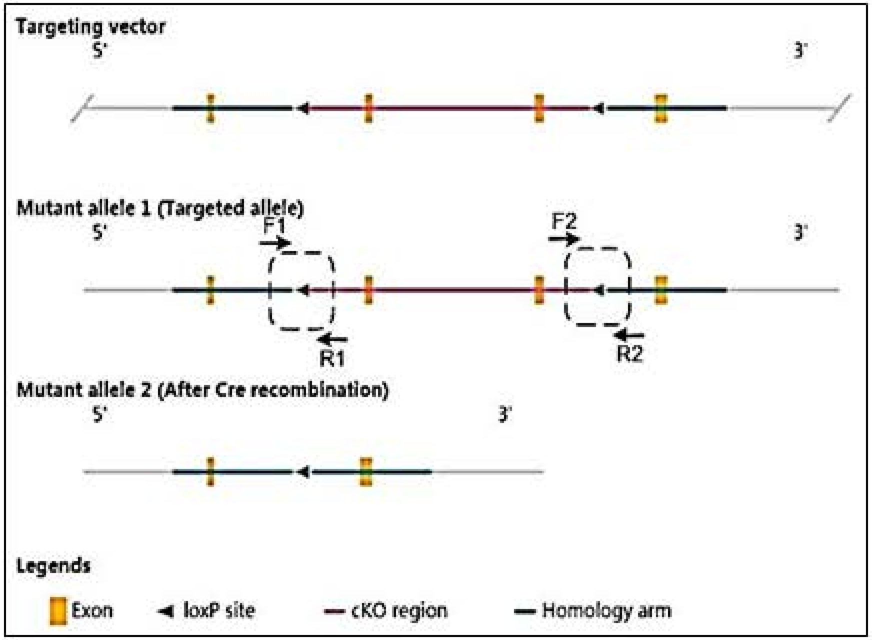

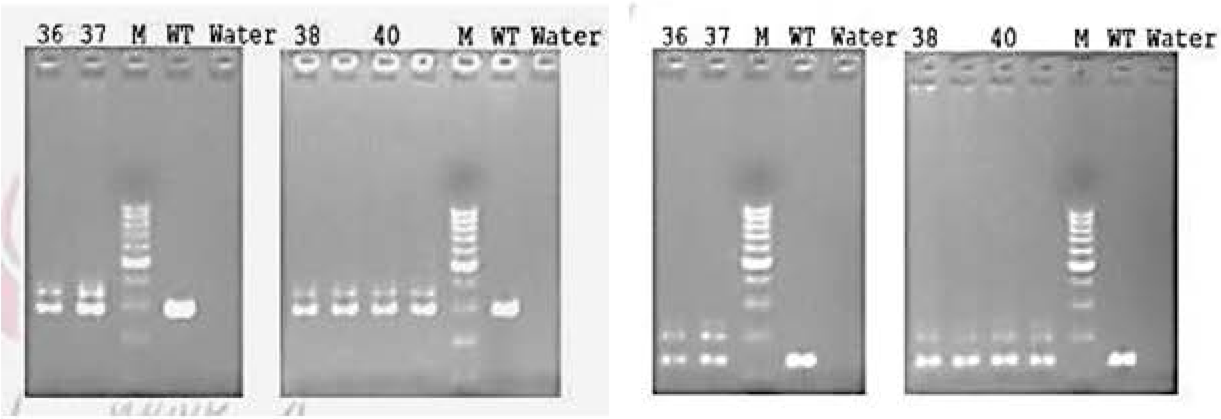

PCR using primer pair F1 & R1 to detect Floxed mice (WT 304bp; Floxed 366bp)

PCR using promer pair F2 & R2 to detect Floxed mice (WT 136bp; Floxed 200bp)

Figure S4

Targeting vector for preparation of TRE-TPP2 knockin mouse line

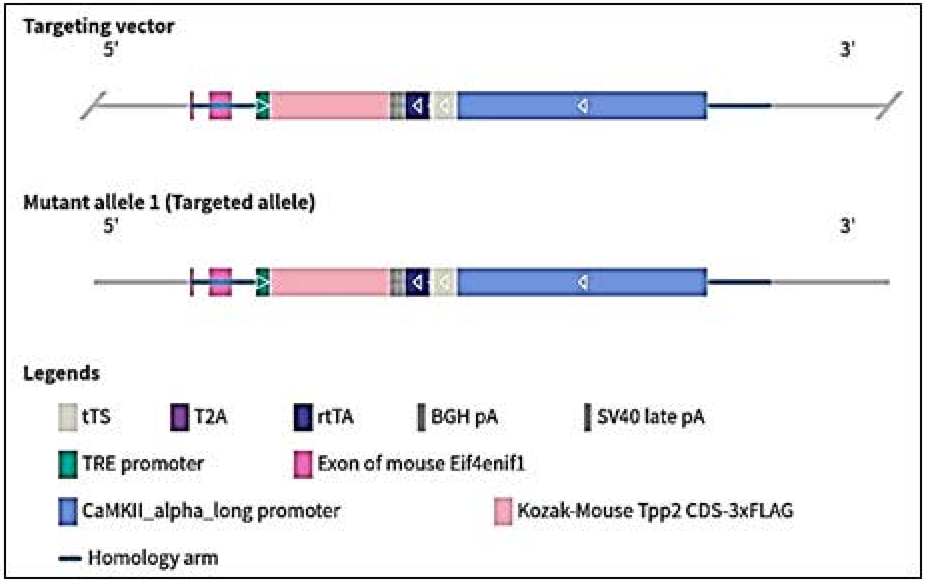

Genotyping strategy of TRE-TPP2 knockin mouse line

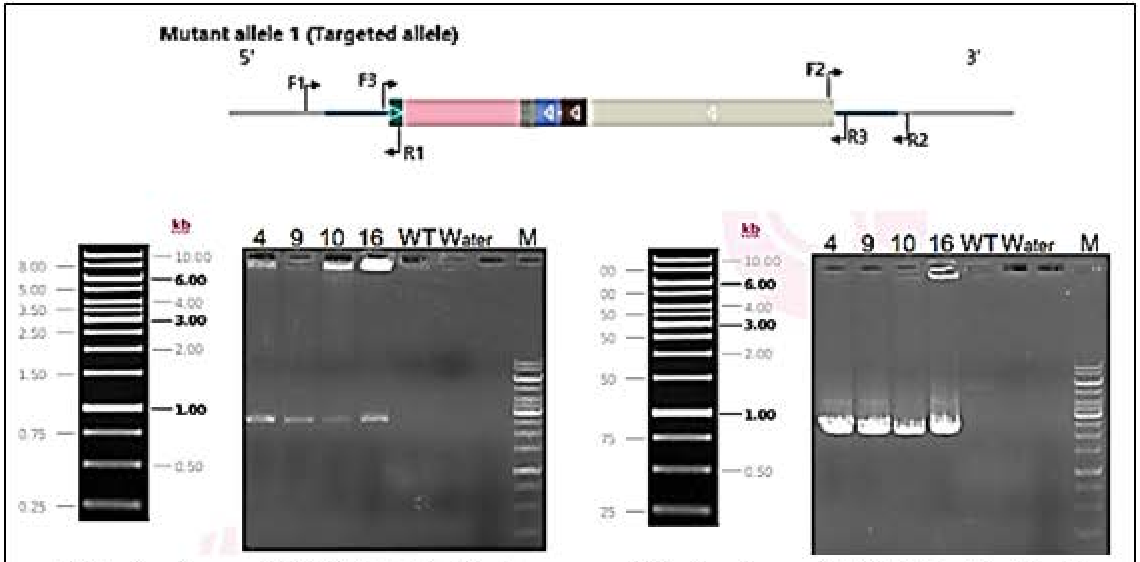

PCR using primer pair F1 & R1 to detect Tg mice
(Positive mice: 3.0 Kb)

PCR using primer pair F2 & R2 to detect Tg mice
(Positive mice: 2.4 Kb)
